# Valorization of *Moringa oleifera* pericarp via semi-synthetic sugar-based enone derivatives with anticancer potential: phytochemical isolation, cytotoxic evaluation, and dual EGFR/CAIX targeting

**DOI:** 10.1038/s41598-026-59686-2

**Published:** 2026-07-04

**Authors:** Mayye Majed, Amal A. Galala, Dina I. A. Othman, Mohamed M. Amer, Sara Abouzeid

**Affiliations:** 1 Pharmacognosy Department, Faculty of Pharmacy, Mansoura University, Mansoura, 35516 Egypt; 2 Pharmacognosy Department, Faculty of Pharmacy, Horus University in Egypt (HUE), New Damietta, 34517 Egypt; 3 Department of Pharmaceutical Organic Chemistry, Faculty of Pharmacy, Mansoura University, Mansoura, 35516 Egypt

**Keywords:** *Moringa oleifera*, agro-waste, semi-synthesis, anticancer activity, CAIX, EGFR-TK, Biochemistry, Biotechnology, Cancer, Chemical biology, Chemistry, Drug discovery

## Abstract

*Moringa oleifera* pericarp, an underutilized agro-waste, was investigated as a potential source of anticancer agents. In this study, bioassay-guided fractionation of the pericarp extract led to the identification of the ethyl acetate fraction as the most active fraction, showing notable antioxidant, antimicrobial, and cytotoxic activities. Subsequent purification afforded three compounds: 4-(*α*-L-rhamnopyranosyloxy)-benzaldehyde (**M**_**1**_), 4-(*α*-L-rhamnopyranosyl) benzyl alcohol (**M**_**2**_), and 4-(hydroxymethyl) phenol-1-*O*-*β*-D-glucopyranosyl-(1''→3')-*O*-*α*-L-rhamnopyranoside (**M**_**3**_). The major constituent, **M**_**2**_, was semi-synthetically modified by Steglich esterification with cinnamic acid and crotonic acid to yield two sugar-based enone derivatives, **S1**_**M2**_ and **S2**_**M2**_. Structural elucidation was performed using IR, MS, and NMR spectroscopy. The cytotoxic activity of the isolated and semi-synthesized compounds was evaluated against HepG2 and HCT116 cancer cell lines, and selectivity was assessed using normal WI-38 fibroblasts. Among the tested compounds, **S2**_**M2**_ exhibited the most potent cytotoxicity, with IC_50_ values of 5.97 ± 0.19 µM and 11.52 ± 0.37 µM against HepG2 and HCT116 cells, respectively, together with favorable selectivity. In addition, **S2**_**M2**_ showed strong inhibitory activity against epidermal growth factor receptor tyrosine kinase (EGFR-TK) and carbonic anhydrase IX (CAIX), with IC_50_values of 0.40 ± 0.008 µM and 0.27 ± 0.01 µM, respectively. Molecular docking and 100-ns molecular dynamics simulations supported the stable binding of **S2**_**M2**_ within the active sites of EGFR-TK and CAIX, and MM-GBSA calculations confirmed its favorable binding free energy. Structure–activity relationship analysis suggested that the *α*,*β*-unsaturated carbonyl moiety significantly contributed to the observed anticancer activity. These findings highlight *M. oleifera* pericarp as a promising source of bioactive glycosylated phenolic scaffolds and identify **S2**_**M2**_ as a potential dual-target anticancer lead for further development.

**Supplementary Information:**

The online version contains supplementary material available at 10.1038/s41598-026-59686-2.

## Introduction

**Fig. 1:**
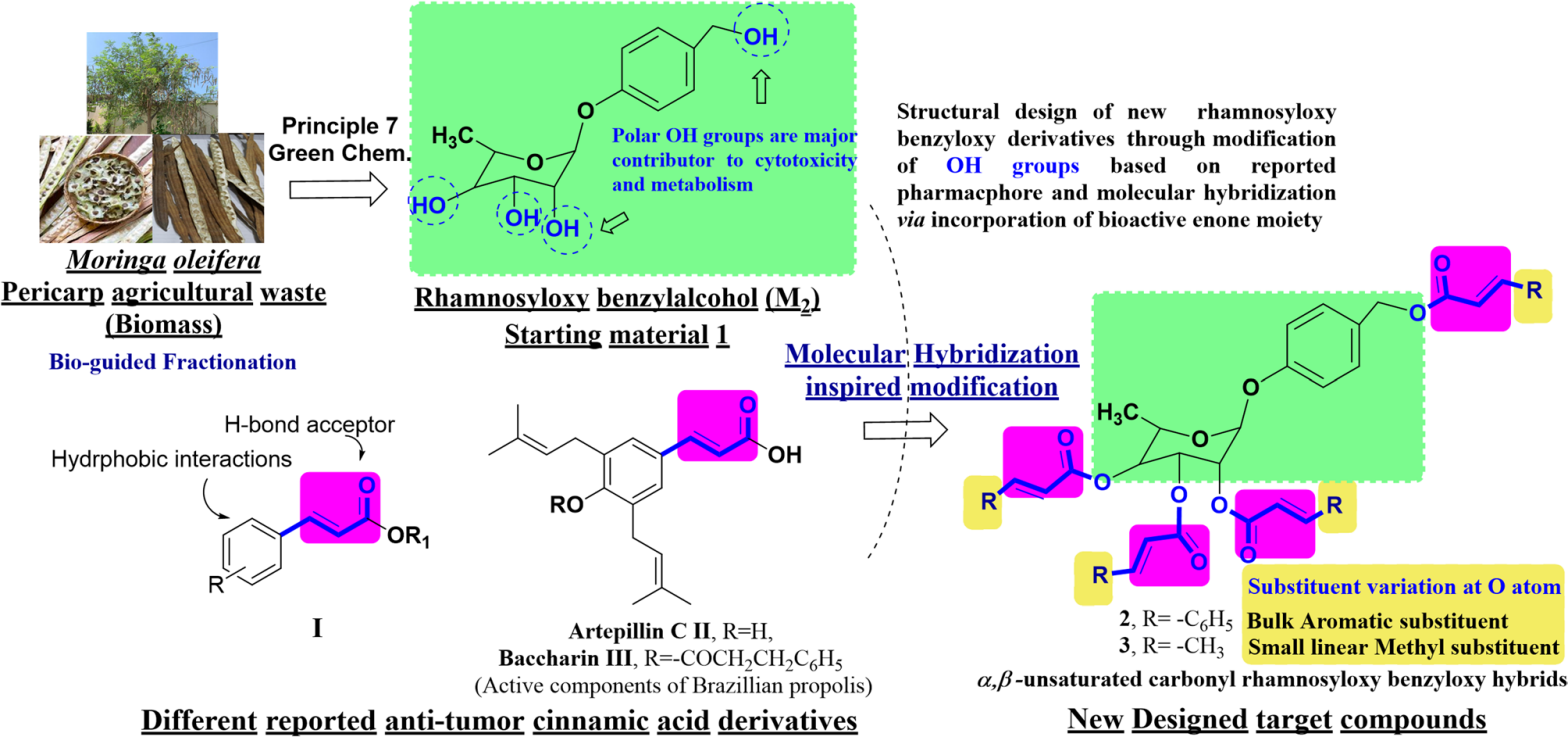
The green design strategy of new synthesized *α*, *β*-unsaturated carbonyl antitumor hybrids through modification of OH functionality of **1** depending on reported pharmacophore and hybridization techniques.

Cancer remains one of the leading causes of mortality worldwide, creating an urgent need for more effective and selective therapeutic agents^1^. Natural products have historically played a central role in drug discovery because of their structural diversity and broad biological activity^2^. In parallel, the use of plant-derived biomass as a renewable feedstock aligns with the principles of green chemistry and offers a sustainable route for generating value-added bioactive compounds^3^. Within this framework, valorization of agricultural waste is especially attractive because it combines environmental benefit with the discovery of new lead scaffolds for therapeutic development. As illustrated in Fig. 1, the present study adopts this concept by using *Moringa oleifera* pericarp, an underutilized agro-waste material, as a renewable source of bioactive metabolites and semi-synthetic anticancer candidates.

*M. oleifera* is a multipurpose tree widely recognized for its nutritional and medicinal properties^4^. Although its leaves, seeds, and pods have been extensively investigated^4–6^, the pericarp remains comparatively underexplored despite its potential as a reservoir of phytochemicals. Previous studies have shown that extracts from different parts of *M. oleifera* exhibit antioxidant, antimicrobial, and anticancer activities, and several classes of compounds, including glucosinolates, isothiocyanates, flavonoids, thiocarbamates, carbamates, and glycosides, have been implicated in these effects^7–12^. However, most available reports have focused on crude extracts or known natural products, leaving the pericarp insufficiently examined as a source of novel anticancer scaffolds. This represents an important gap that motivated the current work.

In the present study, bio-guided fractionation of the pericarp extract led to the isolation of 4-*O*-*α*-L-rhamnopyranosyl benzyl alcohol (**M**_**2**_), the major glycosylated constituent, which served as the key scaffold for further derivatization. Compound **M**_**2**_ was selected for semi-synthetic modification based on its abundance, structural accessibility, and the presence of multiple hydroxyl groups amenable to derivatization. However, these free hydroxyl functionalities present specific medicinal chemistry liabilities, as they confer high polarity and extensive hydrogen-bonding capacity, which limit membrane permeability and oral bioavailability^13^. Additionally, free hydroxyls are susceptible to rapid Phase II metabolic conjugation (e.g., by UGTs and SULTs), leading to poor metabolic stability, and are prone to oxidative degradation, which compromises chemical stability during formulation^14^. Therefore, **M**_**2**_ was modified through esterification of its hydroxyl groups to enhance lipophilicity, improve pharmacokinetic behavior, and mitigate potential toxicity concerns^15^. This strategy, which aligns with green chemistry principles, transforms underutilized plant waste into biologically active lead compounds^3^. Specifically, the isolated scaffold was conjugated with cinnamic and crotonic acids to afford two sugar-based enone derivatives designed to enhance its anticancer potential via the incorporation of a Michael acceptor motif^16–18^.

**Fig. 2:**
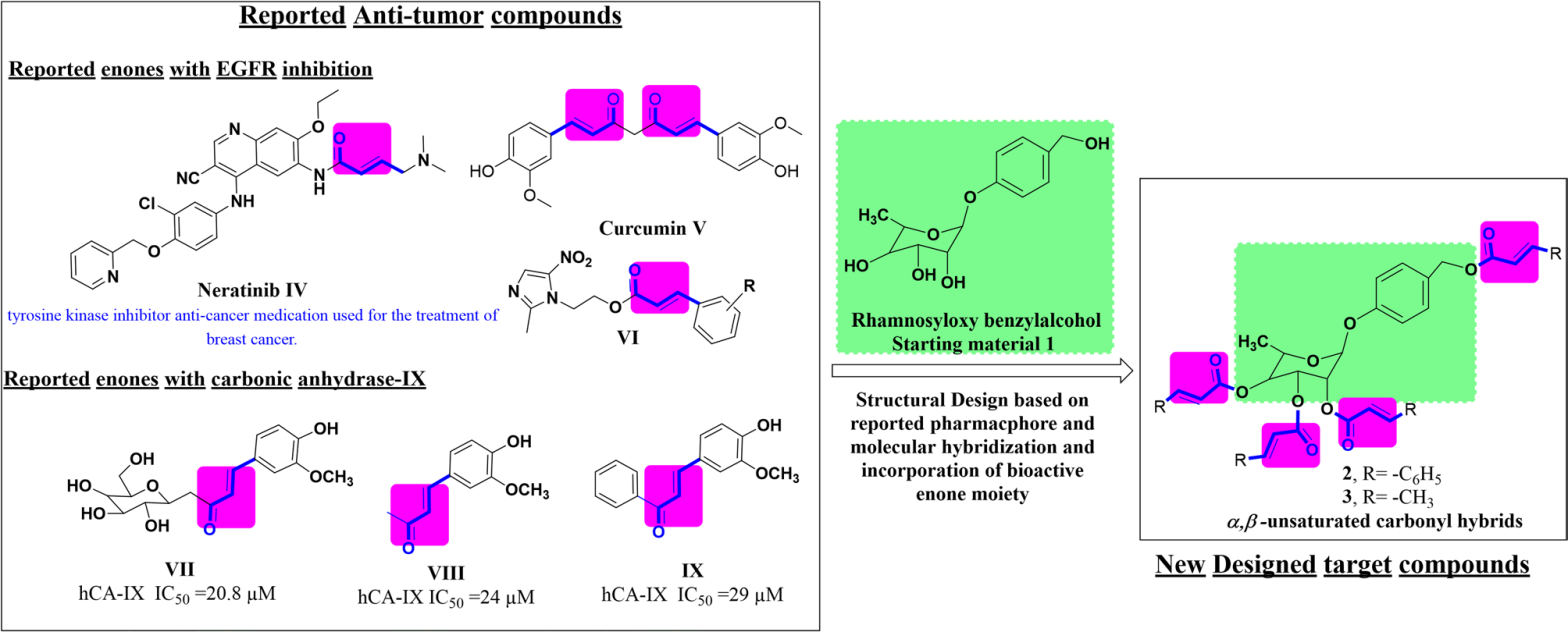
The current rational framework of our designed compounds based on the chemical structure of some reported antitumor lead EGFR/ hCA-IX inhibitors.

The rationale for this design is supported by the known biological relevance of cinnamic and enone-based pharmacophores^17–19^. As shown in Fig. 2, cinnamic acid derivatives and *α*,*β*-unsaturated carbonyl-containing compounds have attracted considerable attention in medicinal chemistry because of their broad-spectrum biological activities, including anticancer effects. The *α*,*β*-unsaturated carbonyl moiety is especially important because it can participate in interactions with diverse biological targets. For example, prenylated cinnamic acid derivatives such as artepillin C and baccharin have demonstrated notable antitumor activity^20^, while compounds such as neratinib and curcumin exemplify the therapeutic relevance of *α*,*β*-unsaturated ketone motifs^21,22^. In addition, cinnamyl-containing structures have been reported as potent inhibitors of cancer-related enzymes, further supporting their use in rational anticancer design^23^.

Another important consideration in this study was the selection of epidermal growth factor receptor tyrosine kinase (EGFR-TK) and carbonic anhydrase IX (CAIX) as molecular targets. EGFR-TK is a well-established driver of tumor cell proliferation and survival, while CAIX is strongly associated with tumor hypoxia and progression^24–26^. Both targets are clinically relevant, and dual inhibition has emerged as a promising strategy for improving anticancer efficacy. Because these enzymes contribute to complementary hallmarks of cancer, targeting both may provide advantages over single-target intervention. Therefore, the present work was designed to evaluate whether the semi-synthetic sugar-based enone derivatives could act as dual EGFR/CAIX inhibitors with improved biological performance (Figs. 1 and 2).

Accordingly, bio-guided fractionation of the pericarp extract identified the ethyl acetate fraction as the most active fraction, based on its antioxidant, antimicrobial, and cytotoxic activities, and led to the isolation of its major constituent compounds, including 4-*O*-*α-*L-rhamnopyranosyl benzyl alcohol (M_2_), which was selected for further derivatization. The isolated compounds and semi-synthetic derivatives were evaluated for cytotoxic activity and enzyme inhibition, and molecular docking studies were performed to explore their binding interactions with EGFR-TK and CAIX. Molecular dynamics simulation was subsequently applied to the most active derivative to assess the stability of its binding mode. To the best of our knowledge, this work uniquely combines valorization of *M. oleifera* pericarp, semi-synthesis of sugar-based enone derivatives, dual EGFR/CAIX targeting, and computational validation within an integrated anticancer research framework.

## Results and discussion

### Antioxidant, antimicrobial, and cytotoxic activities of different fractions of *Moringa oleifera* pericarp

Screening of different fractions of *M. oleifera* for their antioxidant activity revealed that the ethyl acetate fraction of the pericarp exhibited the highest antioxidant activity with inhibition percentages of 68.7 ± 0.04% compared to the standard ascorbic acid (88.1 ± 0.1%) (Table S1).

When assessed for antimicrobial activity, among all tested fractions, the ethyl acetate extract showed strong antibacterial effects against *E. coli*, with an inhibition zone of 20.2 ± 0.2 mm and an activity index of 83.3%, and against *S. aureus*, with a zone of inhibition of 18.17 ± 0.15 mm and an activity index of 78.3% (Table S2). Moderate antifungal activity was also observed against *C. albicans*, with an inhibition zone of 15.1 ± 0.1 mm and an activity index of 57.7% (Table S2). These findings suggest that the ethyl acetate fraction contains potent bioactive constituents responsible for broad-spectrum antimicrobial activity.

The literature highlights the diverse bioactivities of *M. oleifera* extracts, including antioxidant, anti-quorum sensing, and DNA damage-preventive properties in aqueous extracts derived from its leaves, fruits, and seeds^27^. Moreover, acetone extracts of *M. oleifera* pod husks have shown sustained post-antibiotic effects against both Gram-positive and Gram-negative bacteria, indicating their potential for extended dosing regimens^28^. In addition, when used in combination with conventional antibiotics, *M. oleifera* extracts have been reported to target bacterial triggers of autoimmune inflammatory disorders^29^.

**Fig. 3:**
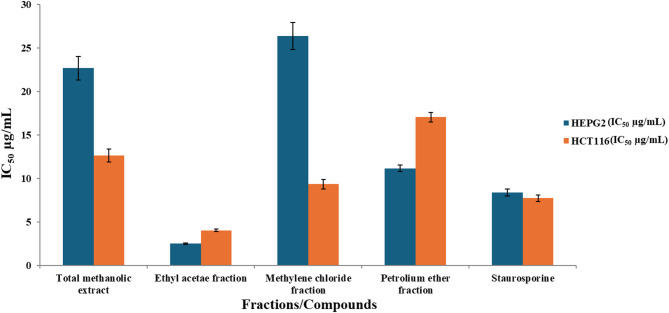
IC_50_ of cytotoxic activity of different fractions of *Moringa oleifera* Pericarp extract using MTT assay. All data are presented as mean value ± SD for three independent experiments.

Regarding cytotoxic activity, the ethyl acetate fraction exhibited potent activity among all tested fractions against both liver and colon cancer cell lines, with IC₅₀ values of 2.5 ± 0.08 µg/mL and 4.04 ± 0.13 µg/mL, respectively, as determined by the MTT assay (Tables S10-S13; Fig. 3). These results are more effective compared to the reference drug staurosporine, which showed IC₅₀ values of 8.4 ± 0.41 µg/mL and 7.75 ± 0.35 µg/mL, respectively (Tables S10-S13; Fig. 3). Accordingly, this fraction was selected for further chromatographic separation to isolate its active constituents. Previous studies have reported the anti-proliferative effects of *M. oleifera* fruit and leaf extracts, which may account for its well-known therapeutic potential as the “miracle tree”^30^. Specifically, *M. oleifera* fruit extract has been shown to induce anti-proliferative effects against HepG2 (human hepatocellular carcinoma cells) via ROS-mediated apoptosis and caspase-3 activation^11^.

### Bio-guided isolation of active constituents from ethyl acetate fraction of *Moringa oleifera* pericarp

**Fig. 4:**
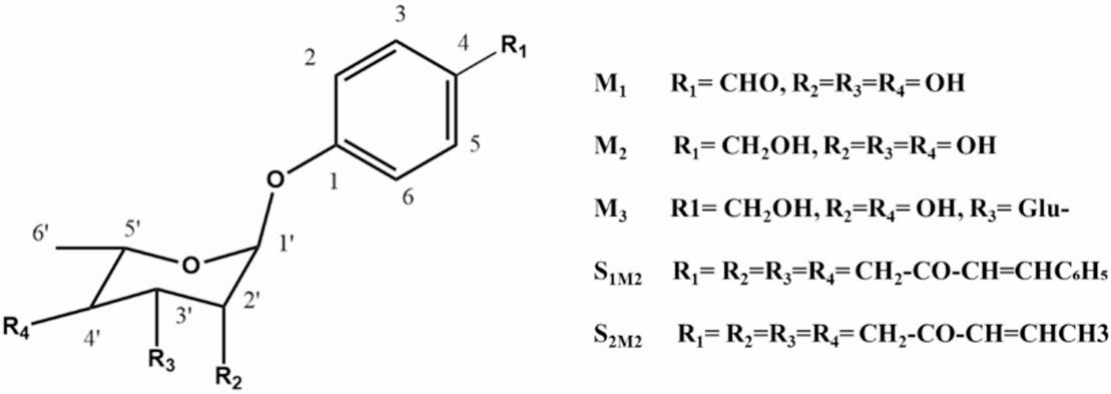
Structures of the isolated compounds from *the Pericarp of Moringa oleifera* active fractions and semi-synthetic compounds: 4-(*α*-L-rhamnopyranosyloxy)-benzaldehyde (**M**_**1**_), 4-(*α*-L-rhamnopyranosyl) benzyl alcohol (**M**_**2**_), 4-(hydroxymethyl) phenol-1-O-*β*-D-glucopyranosyl-(1''→3')-O-*α*-L-rhamnopyranoside (**M**_**3**_), 4-(*α*-L-rhamnosyloxy) benzyl tetracinnamate derivative (**S**_**1M2**_), 4-(*α*-L-rhamnosyl) benzyl tetra crotonate derivative (**S**_**2M2**_).

The ethyl acetate fraction of *M. oleifera* pericarp was subjected to normal-phase silica gel column chromatography, leading to the isolation of three compounds: **M**_**1**_, **M**_**2**_, and **M**_**3**_ (Fig. 4). Compound **M**_**1**_, previously reported from *M. oleifera* seeds, is identified here for the first time from the pericarp. Its NMR data, Table S3, matched previously published data for 4-(*α*-L-rhamnopyranosyloxy)-benzaldehyde^31^, which has also been synthetically prepared^32^. Compound **M**_**2**_ showed NMR data (Table S4), consistent with 4-(*α*-L-rhamnopyranosyl) benzyl alcohol, a compound previously isolated from both *M. stenopetala*^33^ and *M. oleifera* seeds^34^. This study represents the first report of its isolation from *M. oleifera* pericarp, and notably, in high yield. According to Lewerenz et al. (2021)^35^, this compound is likely a degradation product of 4-hydroxybenzyl isothiocyanate, produced via myrosinase-catalyzed hydrolysis of sinalbin^36^. Compound **M**_**3**_ is tentatively identified as 4-(hydroxymethyl) phenol- 1-*O*-*β*-D-glucopyranosyl-(1''→3')-*O*-*α*-L-rhamnopyranoside (Table S5). It was previously reported only once from *Moringa* seeds^34^; thus, this study marks its second report from nature and the first from the pericarp of *M. oleifera*. For more details of structure elucidation, see the supporting information (Tables S3-S5, Figures S1-S11). Overall, the isolation of these three glycosylated compounds confirms that the pericarp contains structurally diverse phenolic constituents with potential biological relevance. Among the isolated constituents, **M**_**2**_ was selected as the main scaffold for further semi-synthetic modification based on its abundance and suitable hydroxyl functionality.

### Semi-synthesis of enone derivatives from isolated compound M_2_

**Fig. 5:**
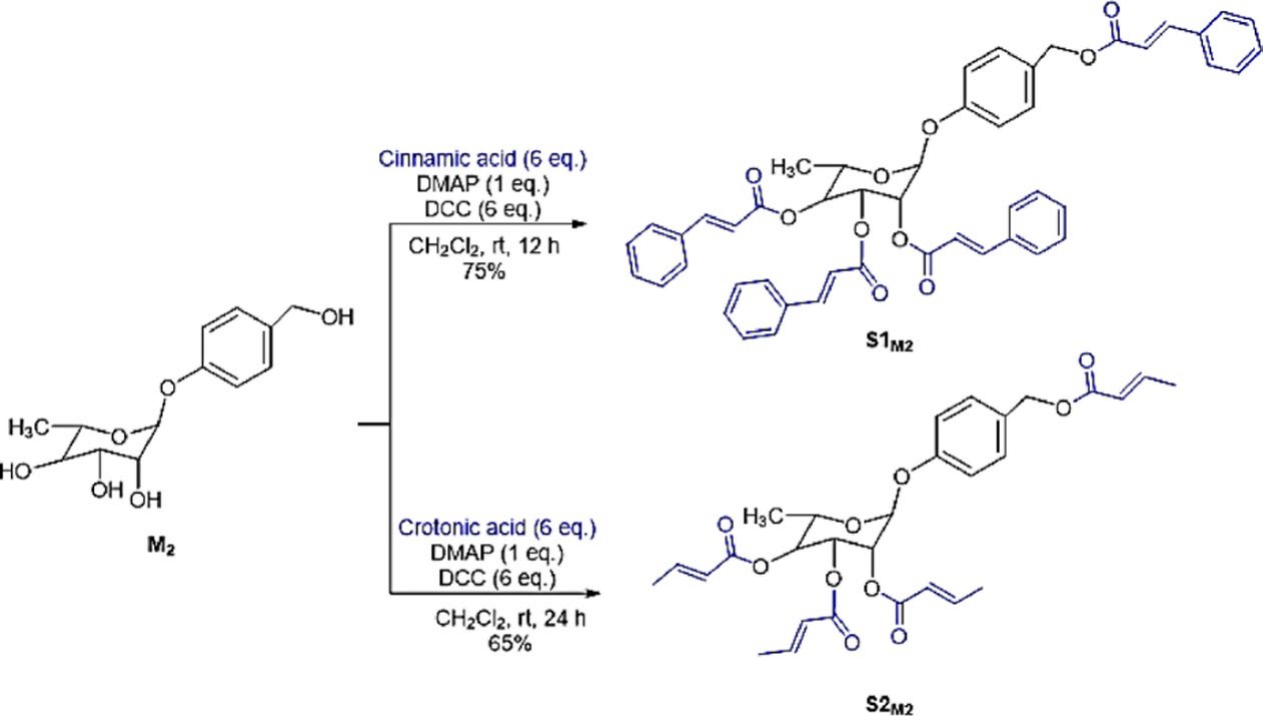
Scheme of semi-synthesis of target-designed sugar-based enone derivatives from **M**_**2**_.

The isolated compound **M**_**2**_ with the skeleton 1, 2-(4-(hydroxymethyl) phenoxy)-6-methyltetrahydro-*2 H*-pyran-3, 4, 5-triol is considered the main scaffold for our newly semi-synthesized target hybrids **S1**_**M2**_ and **S2**_**M2**_ (Fig. 5). Steglich esterification reaction of cinnamic acid or crotonic acid with alcohol derivative **M**_**2**_ in the presence of dicyclohexylcarbodiimide (DCC) and 4-dimethylaminopyridine (DMAP) afforded our designed target compounds **S1**_**M2**_ and **S2**_**M2**_, respectively, according to the previously reported procedure with some minor modifications^37^. The structures of the obtained derivatives **S1**_**M2**_ and **S2**_**M2**_ were characterized and confirmed based on the spectroscopic data (^1^H-NMR, APT, IR and MS) (Tables S6- S9, Figures S12-S20). The spectroscopic data obtained confirmed the incorporation of all free OH functionalities of our starting **M**_**2**_ under the reaction conditions illustrated in Fig. 5 as targeted in our research design rationale.

### Cytotoxic activity of the isolated and semi-synthetic compounds

The present study was designed to synthesize two novel potential sugar-based anticancer agents incorporating enone functionality **S1**_**M2**_ and **S2**_**M2**_, using a structure-based drug design approach Fig. 2. These newly synthesized enone hybrids, along with isolated compounds from the pericarp, were evaluated for their in vitro cytotoxicity using the MTT assay against liver (HepG2) and colon (HCT116) cancer cell lines. Among the tested compounds, **S2**_**M2**_ exhibited the most potent cytotoxic effect, with IC₅₀ values of 5.97 ± 0.19 µM and 11.52 ± 0.37 µM against HepG2 and HCT116 cells, respectively, surpassing the reference drug staurosporine. In contrast, the remaining compounds showed moderate cytotoxic activity (Table 1; Tables S14–S17).

**Table 1:** IC_50_ values of isolated and semi-synthesized compounds against HepG-2 and HCT116 cancer cell lines, EGFR-TK and CAIX enzyme inhibition. All data are presented as mean value ± SD for three independent experiments.

Compound	IC_50_ (µM)			
	MTT		EGFR-TK	CAIX
	**HepG2**	**HCT116**		
**M**_**1**_	54.13 ± 0.19	124.54 ± 0.44	1.28 ± 0.014	2.34 ± 0.03
**M**_**2**_	29.45 ± 0.47	55.75 ± 0.88	0.39 ± 0.004	1.01 ± 0.01
**M**_**3**_	12.21 ± 0.53	43.08 ± 1.69	0.12 ± 0.002	0.44 ± 0.01
**S1**_**M2**_	20.46 ± 0.95	31.45 ± 1.46	0.85 ± 0.026	1.21 ± 0.04
**S2**_**M2**_	5.97 ± 0.19	11.52 ± 0.37	0.40 ± 0.008	0.27 ± 0.01
**Staurosporine**	18.00 ± 0.41	16.62 ± 0.38	……….	…………
**Erlotinib**	…………….	…………	0.11 ± 0.01	………….
**Acetazolamide**	………….	…………	………….	0.49 ± 0.01

**Fig. 6:**
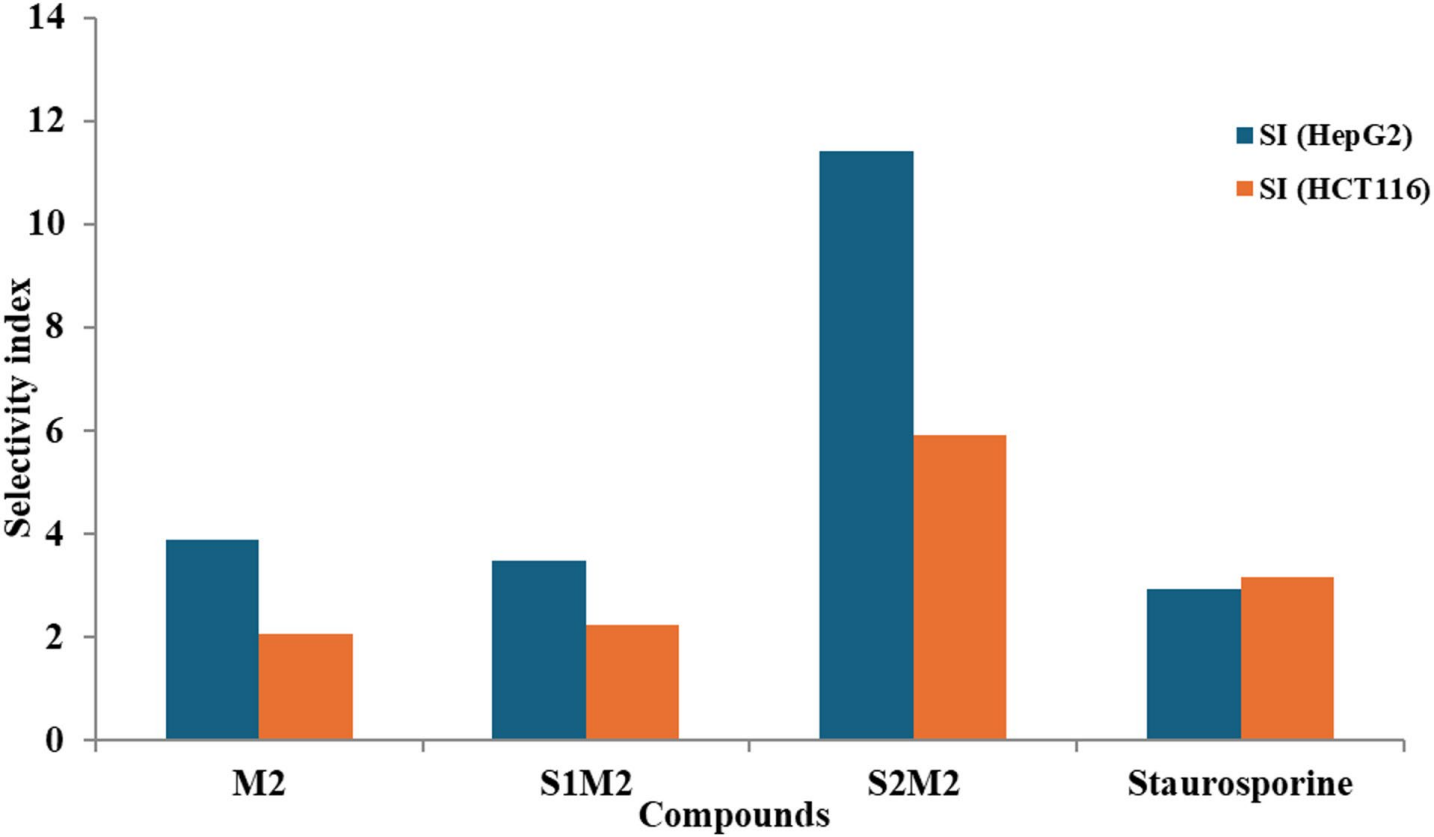
Selectivity index (SI) values of **M**_**2**_ and its semi-synthetic derivatives (**S1**_**M2**_ and **S2**_**M2**_) against HepG2 and HCT116 cancer cell lines in comparison with the reference compound staurosporine.

To evaluate the safety and selectivity of the isolated compound **M**_**2**_ and its semi-synthesized derivatives (**S1**_**M2**_ and **S2**_**M2**_), their cytotoxic effects were assessed against the normal lung fibroblast cell line WI-38 alongside cancer cell lines (Tables S18–S20). The IC₅₀ values against WI-38 were 114.36 ± 1.88, 70.92 **±** 3.04 and 68.30 ± 2.31 µM for **M**_**2**_, **S1**_**M2**_, and **S2**_**M2**_, respectively, indicating comparatively lower toxicity toward normal cells (Table S20). Notably, **S2**_**M2**_ exhibited the highest selectivity indices, with values of 11.43 (HepG2) and 5.93 (HCT116), compared to the reference compound, which showed selectivity indices of 2.90 and 3.16, respectively (Table S20, Fig. 6). These findings demonstrate that semi-synthetic modification of **M**_**2**_, particularly in the case of **S2**_**M2**_, enhances selective cytotoxicity toward cancer cells while maintaining a favorable safety profile. **S2**_**M2**_ showed potent activity, especially against HepG2 cells, with reduced toxicity toward WI-38 cells, highlighting its potential as a promising lead compound for drug development.

Previous studies have demonstrated the chemopreventive potential of *Moringa oleifera* Lam pods. Compound **M**_**1**_, previously isolated, was tested for its cytotoxicity against doxorubicin-resistant human breast cancer cell lines (MCF-7/Adr), where it demonstrated promising activity^38^. These findings align with our results, which highlight the cytotoxic activity of *M. oleifera* and its isolated compounds.

The anticancer potential was further supported by additional in vitro assays, including EGFR TK and CAIX inhibition, confirming the therapeutic promise of *M. oleifera* as a source of anticancer agents.

### EGFR-TK inhibition assay of the isolated and semi-synthetic compounds

The inhibitory activity of the tested compounds against EGFR tyrosine kinase was evaluated in comparison with erlotinib as the reference inhibitor^39^. Among the isolated and semi-synthetic compounds, **M**_**2**_, **M**_**3**_, and **S2**_**M2**_ showed the most notable inhibitory effects, with IC_50_ values of 0.39 ± 0.004, 0.12 ± 0.002, and 0.40 ± 0.008 µM, respectively, while the remaining compounds exhibited weaker activity (Table 1; Table S21). The potent activity of **M**_**3**_ suggests that glycosylated phenolic scaffolds from *Moringa oleifera* may interact effectively with the EGFR active site and may provide a useful framework for further optimization. In contrast, the comparable activity of **M**_**2**_ and **S2**_**M2**_ suggests that semi-synthetic modification of the parent scaffold does not compromise EGFR inhibitory potential and may, in addition, improve its drug-like properties.

### CAIX enzyme inhibition assay of the isolated and semi-synthetic compounds

The inhibitory activity against carbonic anhydrase IX (CAIX) was evaluated in comparison with acetazolamide as the reference inhibitor^40^. Among all tested compounds, **S2**_**M2**_ exhibited the highest inhibitory activity with an IC_50_ value of 0.27 ± 0.01 µM, exceeding that of the reference inhibitor acetazolamide (0.49 ± 0.01 µM) (Table 1; Table S22). This result indicates that crotonate derivatization of **M**_**2**_ significantly improved its affinity toward CAIX and supports the value of introducing an *α*, *β*-unsaturated carbonyl moiety into the parent scaffold^24,25^.

The superior CAIX inhibition shown by **S2**_**M2**_ may be attributed to its optimized fit within the enzyme active site and its ability to engage in multiple noncovalent interactions. In contrast, the parent compound **M**_**2**_ displayed lower activity, suggesting that free hydroxyl-rich glycosylated scaffolds are less effective than their semi-synthetic enone derivatives in this assay. The activity of **M**_**3**_ also suggests that naturally occurring glycosylated phenolics from *Moringa oleifera* can retain meaningful enzyme inhibitory potential. CAIX is a hypoxia-inducible enzyme that contributes to tumor pH regulation, progression, and resistance to therapy, making it a relevant anticancer target. In this context, dual targeting of EGFR and CAIX is particularly attractive because it may interfere with both tumor proliferation and the hypoxic microenvironment that supports malignant growth^24–26^.

Overall, these findings identify **S2**_**M2**_ as the most promising dual EGFR/CAIX inhibitor among the compounds investigated and provide a strong basis for the subsequent docking and molecular dynamics analyses.

### Molecular docking study of isolated and semi-synthetic compounds targeting EGFR-TK and CAIX

To further rationalize the observed biological activity, molecular docking was performed to examine the binding modes of the most active compounds within the active sites of EGFR-TK and CAIX. The binding modes of compounds **M**_**3**_, **S1**_**M2**_, and **S2**_**M2**_ against wild-type EGFR tyrosine kinase (EGFR^wt^ TK) revealed high binding affinities, with binding energies of − 7.15, − 7.17, and − 7.71 kcal/mol, respectively. Compound **M**_**3**_ formed four hydrophobic π-interactions with Leu694, Val702, Ala719, and Lys721. Additionally, it established two hydrogen bonds with Asp831 and Met769, with bond distances of approximately 2.59 and 2.95 Å, respectively (Table 2). In contrast, Compound **S2**_**M2**_ exhibited nine hydrophobic π-interactions involving His781, Leu820, Leu768, Met769, Val702, Leu694, and Cys773. It also formed three hydrogen bonds with Cys773 and Lys692, at distances of 2.13, 2.45, and 2.48 Å, respectively. For comparison, Erlotinib, displayed a binding affinity score of − 7.31 kcal/mol. Erlotinib formed eight hydrophobic π-interactions with Leu694, Ala719, Leu820, Lys721, and Val702, and a single hydrogen bond with Met769 at a distance of 1.97 Å (Table 2).

**Table 2:** Molecular docking scores and type of binding interaction for EGFR-TK enzymes.

Compound	Binding energy	RMSD value(Å)	2D pose	3D pose
**M**_**1**_	-5.89	1.14		
**M**_**2**_	-5.87	1.44		
**M**_**3**_	-7.15	1.39		
**S1**_**M2**_	-7.17	3.39		
**S2**_**M2**_	-7.71	1.55		
**Erlotinib**	-7.31	0.93		

The binding mode of compound **S2**_**M2**_ against carbonic anhydrase IX (CAIX) exhibited a binding energy of − 8.55 kcal/mol, indicating a strong interaction (Table 3). Compound **S2**_**M2**_ formed eight hydrophobic π-interactions with Pro201, Leu197, Val130, Leu91, His64, Leu140, Val121, and Val142. These interactions were further stabilized by two hydrogen bonds and one ion–metal interaction with Met1, Thr199, and the Zn²⁺ ion at position 301, with bond distances of 3.00 Å and 2.97 Å, respectively. In comparison, the co-crystallized ligand in the CAIX complex (PDB ID: 6S03) showed a binding affinity of − 7.45 kcal/mol. It established ten hydrophobic π-interactions involving Trp208, His96, Leu91, Val130, Pro201, Val134, and Leu197. Additionally, it formed two hydrogen bonds with Thr198, at distances of 2.09 Å and 1.84 Å.

**Table 3:** The molecular docking scores and type of binding interaction for Carbonic anhydrase IX inhibition.

Compound	Binding energy	RMSD value (Å)	2D pose	3D pose
**M**_**1**_	-6.95	1.61		
**M**_**2**_	-7.25	1.25		
**M**_**3**_	-7.32	1.55		
**S1**_**M2**_	-8.12	2.20		
**S2**_**M2**_	-8.55	1.11		
**Co-crystallised ligand** **(PDB ID: 6S03)**	-7.45	1.03		

Notably, the EGFR binding pattern of **S2**_**M2**_ is in line with the behavior of established EGFR inhibitors such as erlotinib, which exert activity through stable occupation of the kinase domain. Likewise, the CAIX binding profile supports the interpretation that the enone derivative is able to exploit the structural features of the CAIX active site more efficiently than the parent scaffold. Overall, the docking results provide a structural explanation for the superior dual inhibitory activity of **S2**_**M2**_ and strengthen the view that semi-synthetic modification of **M**_**2**_ enhanced its target engagement across both enzymes. These findings also justify the subsequent molecular dynamics analysis of **S2**_**M2**_, which was used to verify the stability of the docked complexes over time.

### Molecular dynamics (MD) simulation study of compound S2_M2_

MD simulation was processed for 100 ns to evaluate the molecular stability of compound **S2**_**M2**_ with the CAIX and EGFR-TK target pockets. The displacement of the ligand within the target pockets was measured by using root mean square deviation (RMSD). Finally, candidate **S2**_**M2**_ interactions were also analyzed and evaluated in detail.

#### Protein and ligand RMSD and RMSF analysis

**Fig. 7:**
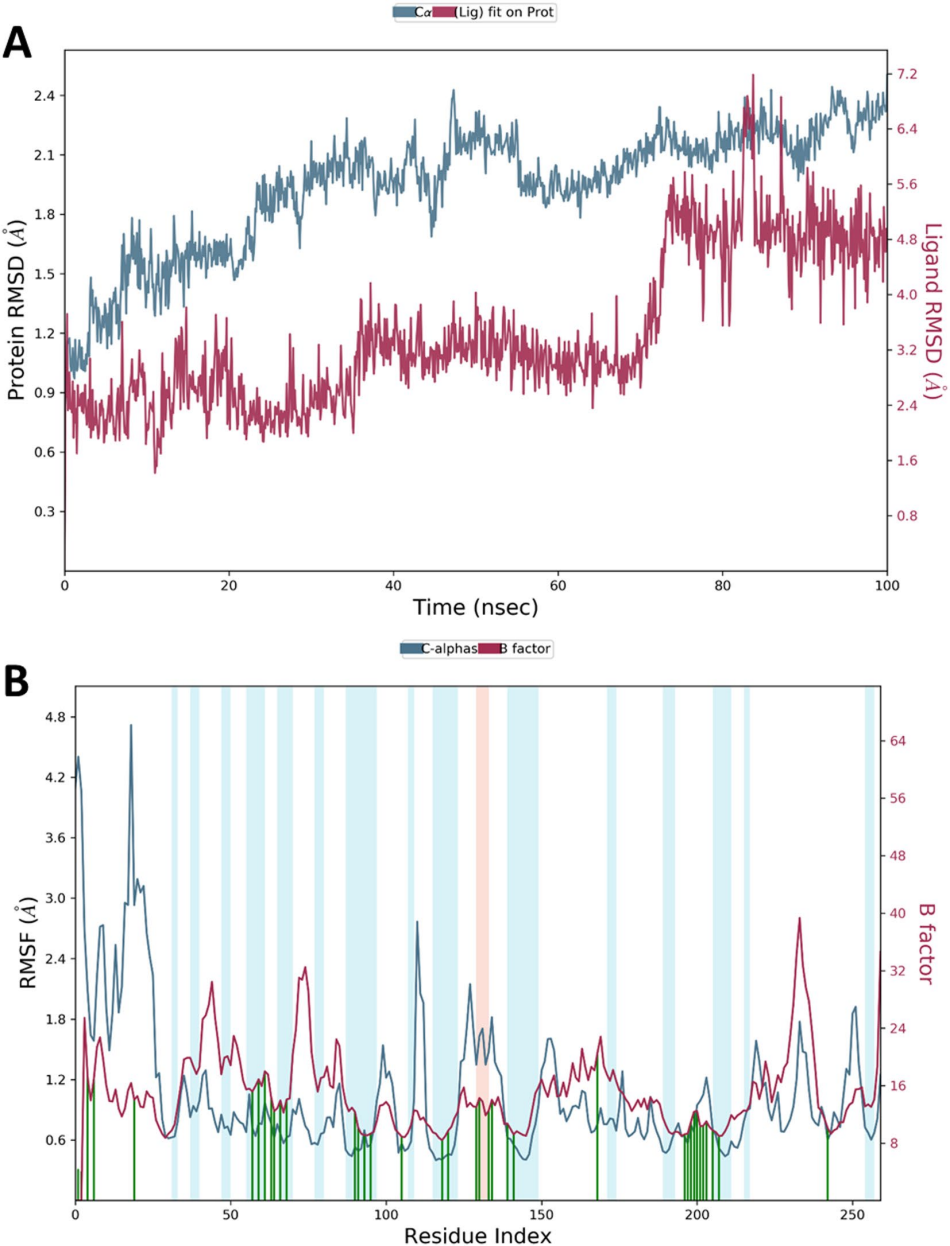
The RMSD and RMSF of Compound **S2**_**M2**_ against carbonic anhydrase IX (CAIX) over 100 ns.

In the present study, compound **S2**_**M2**_ was docked against the CAIX and EGFR-TK target proteins and showed a promising binding affinity among other tested compounds. Therefore, compound **S2**_**M2**_ was selected for further molecular dynamics (MD) simulation studies. The structural stability of the protein structures was monitored through the C atoms (blue line) of the protein concerning their initial position. To validate this interaction, the simulation to 100 ns was processed. At first, the complex of CAIX /Compound **S2**_**M2**_ was inserted into the simulation system. MD simulation analyses of compound **S2**_**M2**_ in complex with CAIX reveal a protein structure that remains predominantly stable over a 100 ns trajectory, as indicated by average protein backbone RMSD values fluctuating between 1.5 and 2.2 Å. The ligand RMSD, calculated by fitting to the protein, initially remains low (< 1.5 Å) and stable within 3.2 to 4.8 Å, but showed minor fluctuation after approximately 65–70 ns, suggesting an increase in ligand conformational mobility or a possible shift in binding orientation during the later stage of the simulation as shown in Fig. 7A. Additionally, Root mean square fluctuations (RMSF) assessed on a per-residue basis demonstrates that most CAIX residues display limited mobility (< 2.0 Å RMSF), except for certain terminal and loop regions, which are inherently more flexible. Compound **S2**_**M2**_ showed minor fluctuations and many movements inside the pocket of CAIX at 10–30, and 130–150 amino acids areas, which leads to some conformational changes in protein skeleton with minor effect on the protein target interactions (Fig. 7B).

**Fig. 8:**
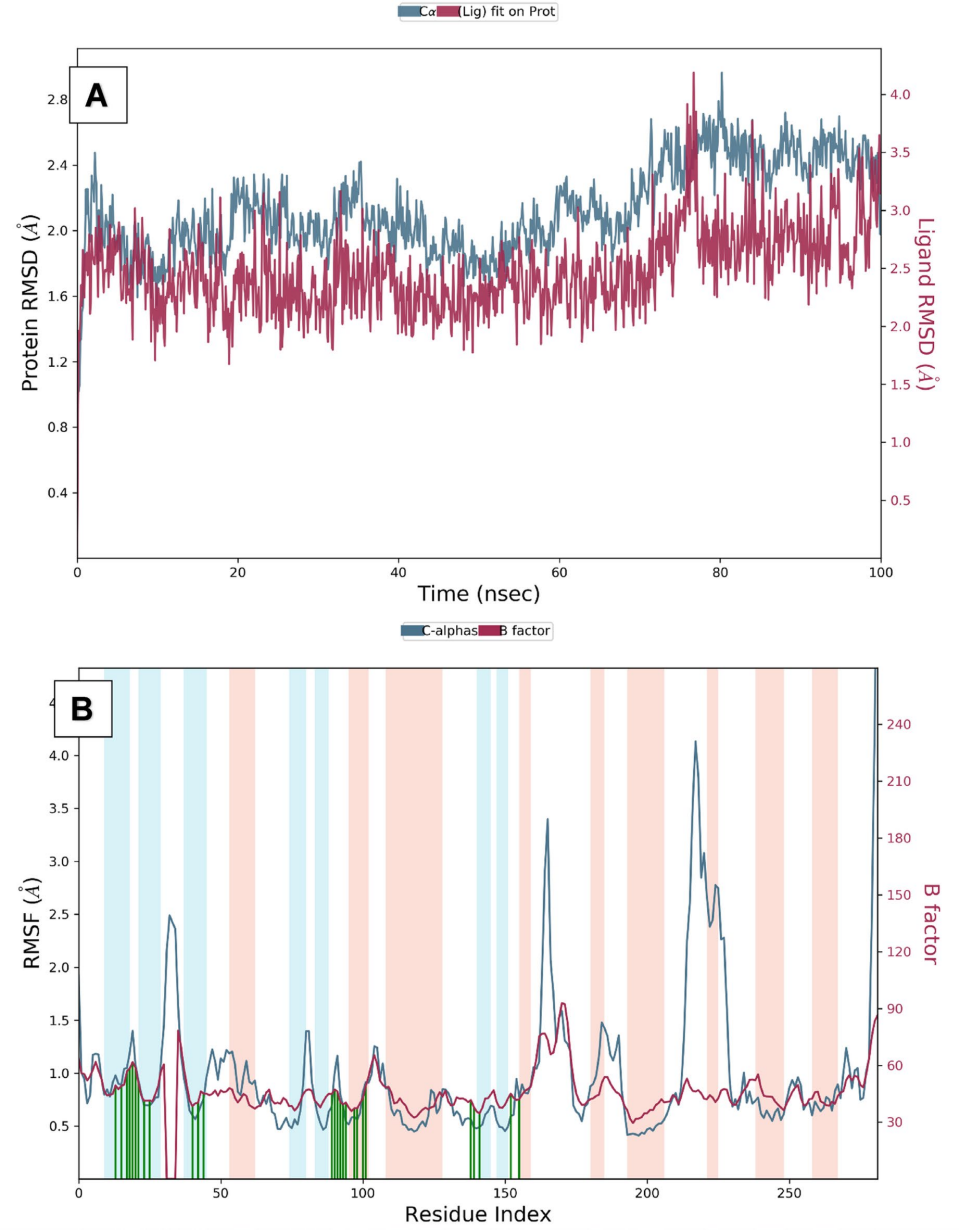
The RMSD and RMSF of Compound **S2**_**M2**_ against **EGFR-TK** over 100 ns.

On the other hand, simulation of compound **S2**_**M2**_ in complex with EGFR tyrosine kinase exhibited that the protein showed structural stability over the simulation time, and Cα RMSD values that ranged between 1.8 and 2.4 Å. The RMSD profile shows only moderate fluctuation, with a slight increase in the latter part of the simulation, but no indication of protein unfolding or major deviation from the starting structure. The ligand RMSD, measured after fitting on the protein, remains stable and comparable to the protein backbone, generally below 1.5 Å ( within 2.5 to 3.5 Å), indicating that compound **S**_**2M2**_ retains a consistent binding mode for most of the simulation time. A modest increase in ligand RMSD after ~ 75 ns suggests some degree of conformational flexibility or minor pose adjustments within the binding site, without any effect on ligand protein interactions (Fig. 8A).

Root mean square fluctuation (RMSF) analysis reveals that most protein residues have restricted mobility (< 1.5 Å), with elevated flexibility observed in selected loop regions and termini, as expected for EGFR TK (Fig. 8B).

#### Histogram of protein ligand interactions analysis

**Fig. 9:**
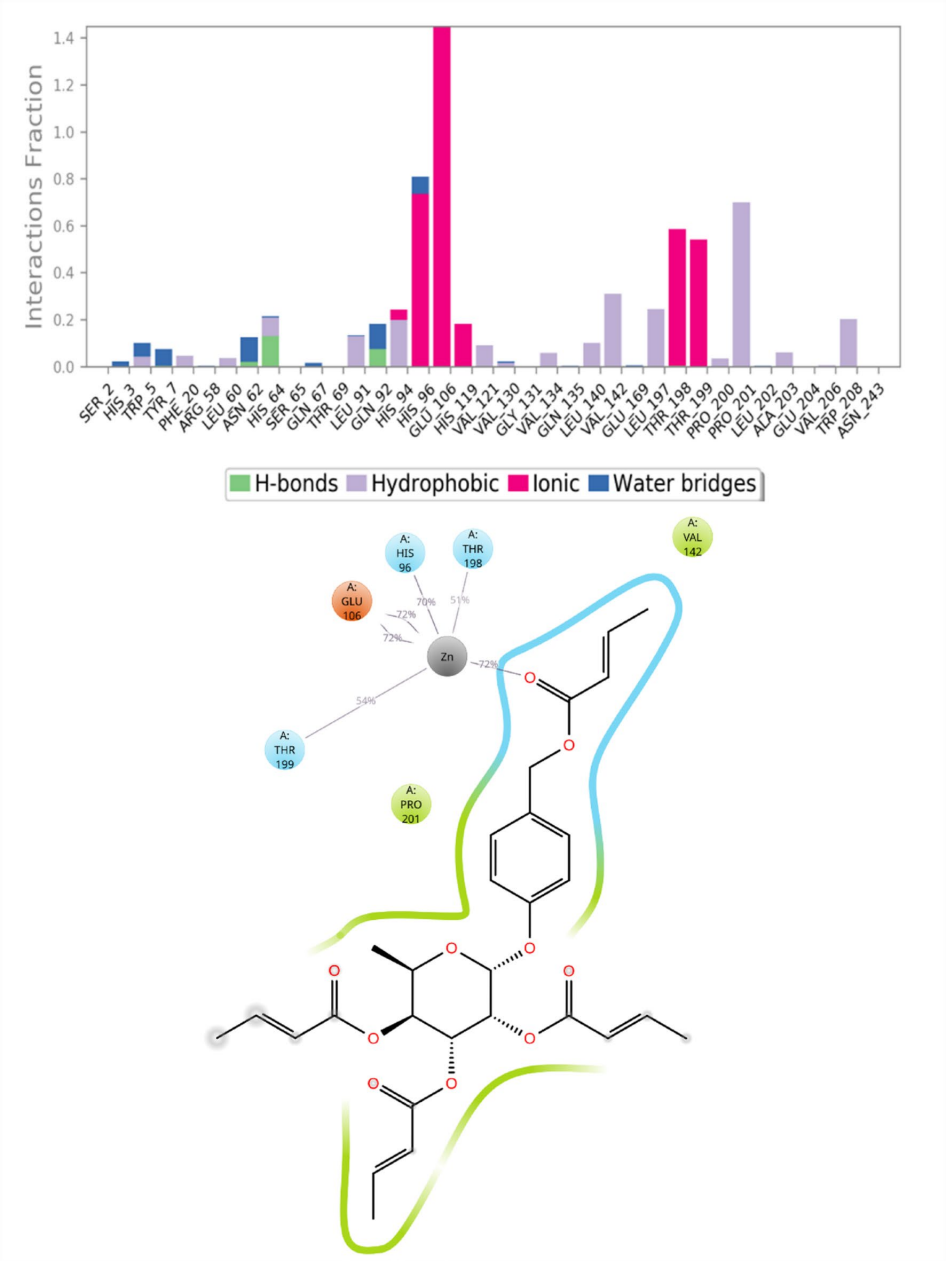
Histogram analysis describes the binding interactions of Compound **S2**_**M2**_ against carbonic anhydrase IX (CAIX) during the simulation time (100 ns), green column represents hydrogen bond, blue column represents polar water linkage and violet column represents hydrophobic π-interactions.

Ligand interaction analysis of compound **S2**_**M2**_ with carbonic anhydrase IX (CAIX) reveals a multifaceted and persistent interaction network within the active site. The most frequent and sustained contacts are ionic interactions with Glu106, evident from an interaction fraction exceeding 1.0, indicating that multiple ionic interactions are maintained for nearly the entirety of the simulation. Additionally, compound **S**_**2M2**_ forms persistent hydrogen bonds with His96 and Thr199, as well as hydrophobic contacts with Pro201 and Thr198. Water-mediated bridges further stabilize the complex, notably with Thr199 and His96 (Fig. 9).

**Fig. 10:**
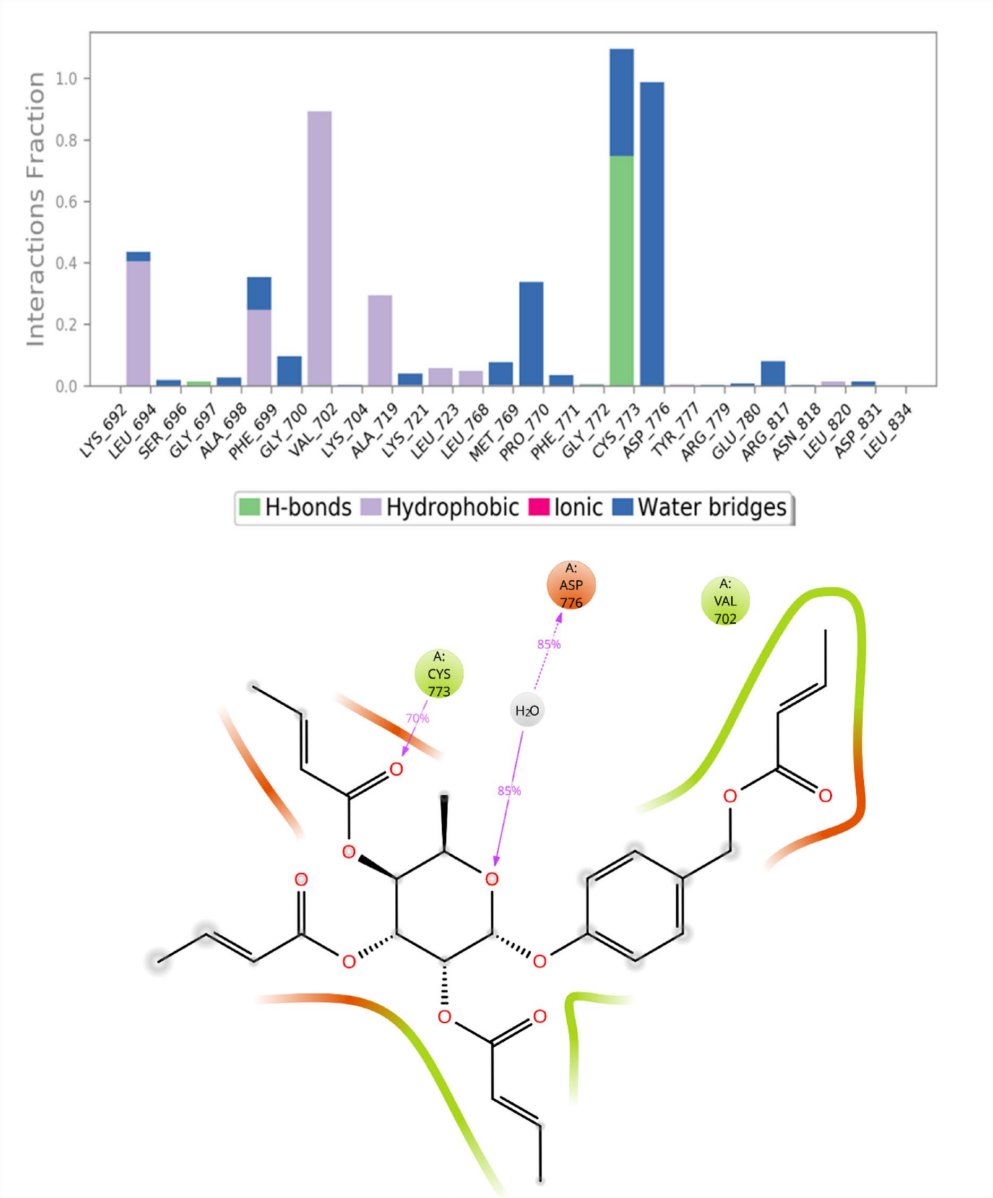
Histogram analysis describing the binding interactions of Compound **S2**_**M2**_ against **EGFR-TK** during the simulation time (100 ns), green column represents hydrogen bond, blue column represents polar water linkage and violet column represents hydrophobic π-interactions.

Additionally, MD simulation of **S2**_**M2**_/EGFR tyrosine kinase complex demonstrated sustained ATP-binding site engagement through a multivalent interaction network combining dominant hydrophobic contacts with water-mediated hydrogen bonding. Val702 emerged as the primary sharing residue (91% interaction fraction within the P-loop), supported by another hydrophobic interactions with Leu694 (43%), Phe699 (36%), and Ala719 (35%), establishing a gradient of Van der Waals complementarity across the adenine-binding cleft. Moreover, water molecules functioned as critical mediating agents in the protein-ligand interface, with Cys-773 forming water-bridge contacts (32% occupancy), hydrogen bonding (70% occupancy) and Asp-776 establishing a highly persistent water-mediated hydrogen bond (85% occupancy) in the hinge region. Collectively, these findings establish compound **S2**_**M2**_ as an ATP-competitive inhibitor achieving high-affinity EGFR binding through hydrophobic (Fig. 10).

**Fig. 11:**
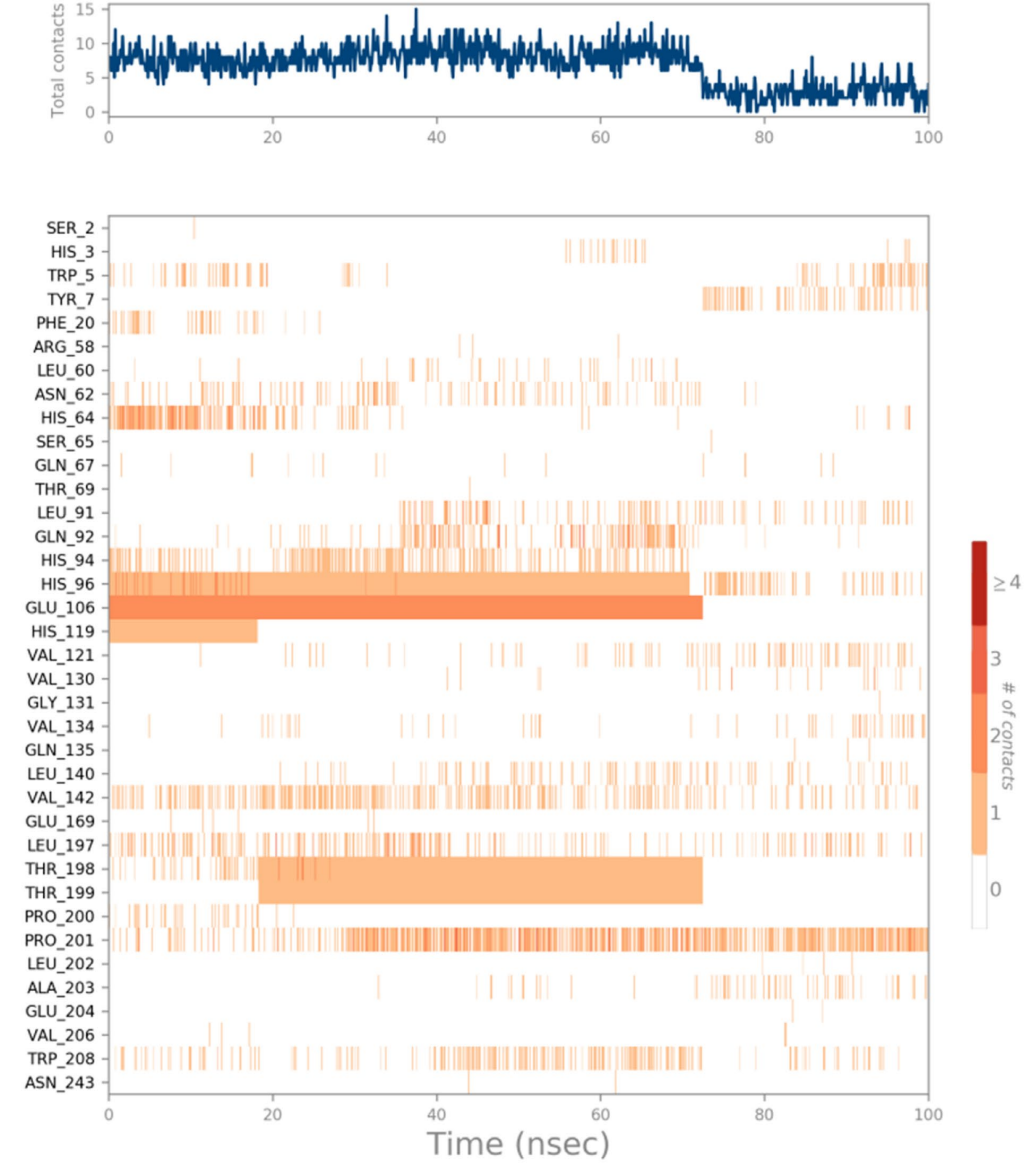
Heat map describing the total interactions within Compound **S2**_**M2**_ against carbonic anhydrase IX (CAIX) during the simulation time.

**Fig. 12:**
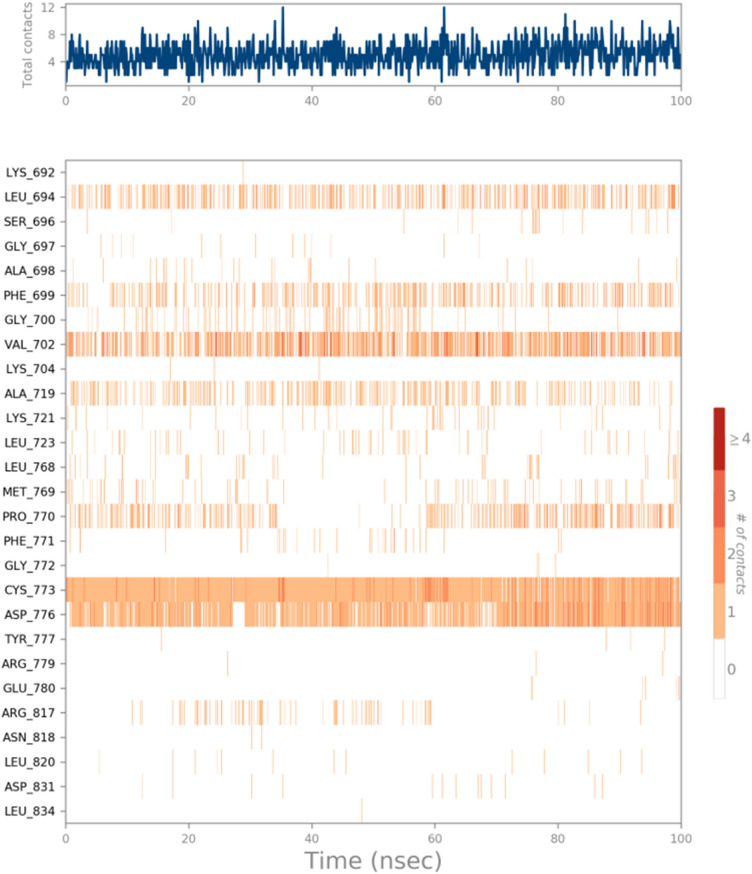
Heat map describing the total interactions within Compound **S2**_**M2**_ against **EGFR-TK** during the simulation time.

To analyze the frequency of the interactions, a heat map was used to monitor these interactions by plotting the number of interactions over time (Figs. 11 and 12), where the dark color indicates more interactions. From the heat map of Fig. 11, it was observed that the highest number of conformations of the protein of CAIX formed up to five interactions. The most interacted amino acids of CAIX with Compound **S2**_**M2**_ are His96, Glu106, His119, Val142, Thr198, Thr199, Pro201, and Trp208. Additionally, the heat map of compound **S**_**2M2**_ with EGFR TK showed persistent primary anchoring residues including Val702, Cys773, and Asp776 demonstrate continuous orange coloration, reflecting uninterrupted binding and validating their critical roles in stabilizing the ligand within the ATP pocket Fig. 12.

#### MM-GBSA calculations

The molecular mechanics, with a generalized Born and surface area solvation (MM–GBSA), were carried out to calculate both the ligand binding strain and free energies for the docked ligand over 100 ns. The ΔG binding energies, Coulombic energies, hydrogen bond energies, Van der Waals forces and lipophilic energies were recorded.

**Fig. 13:**
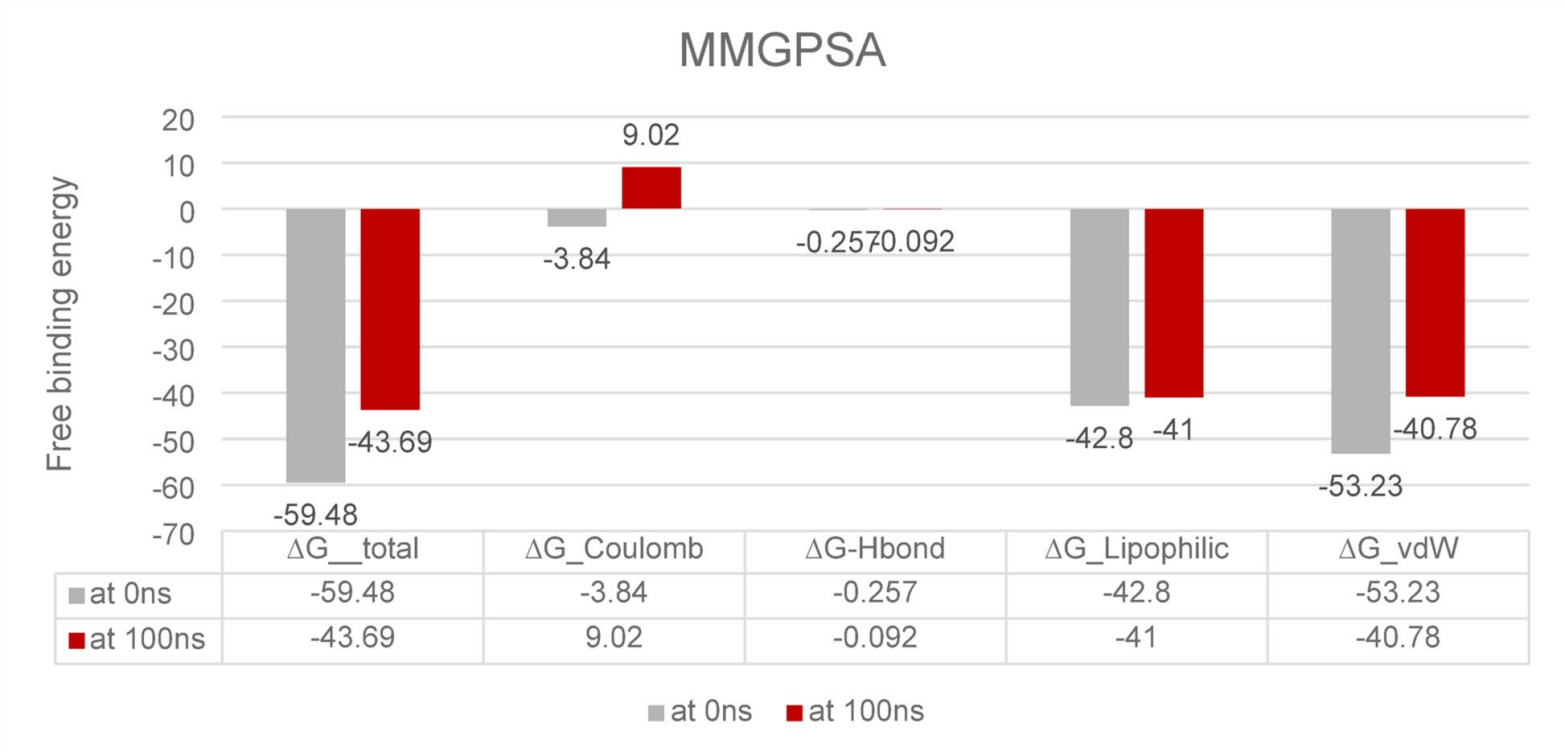
MM-GBSA energies for Compound **S2**_**M2**_ against carbonic anhydrase IX (kcal/mol).

**Fig. 14:**
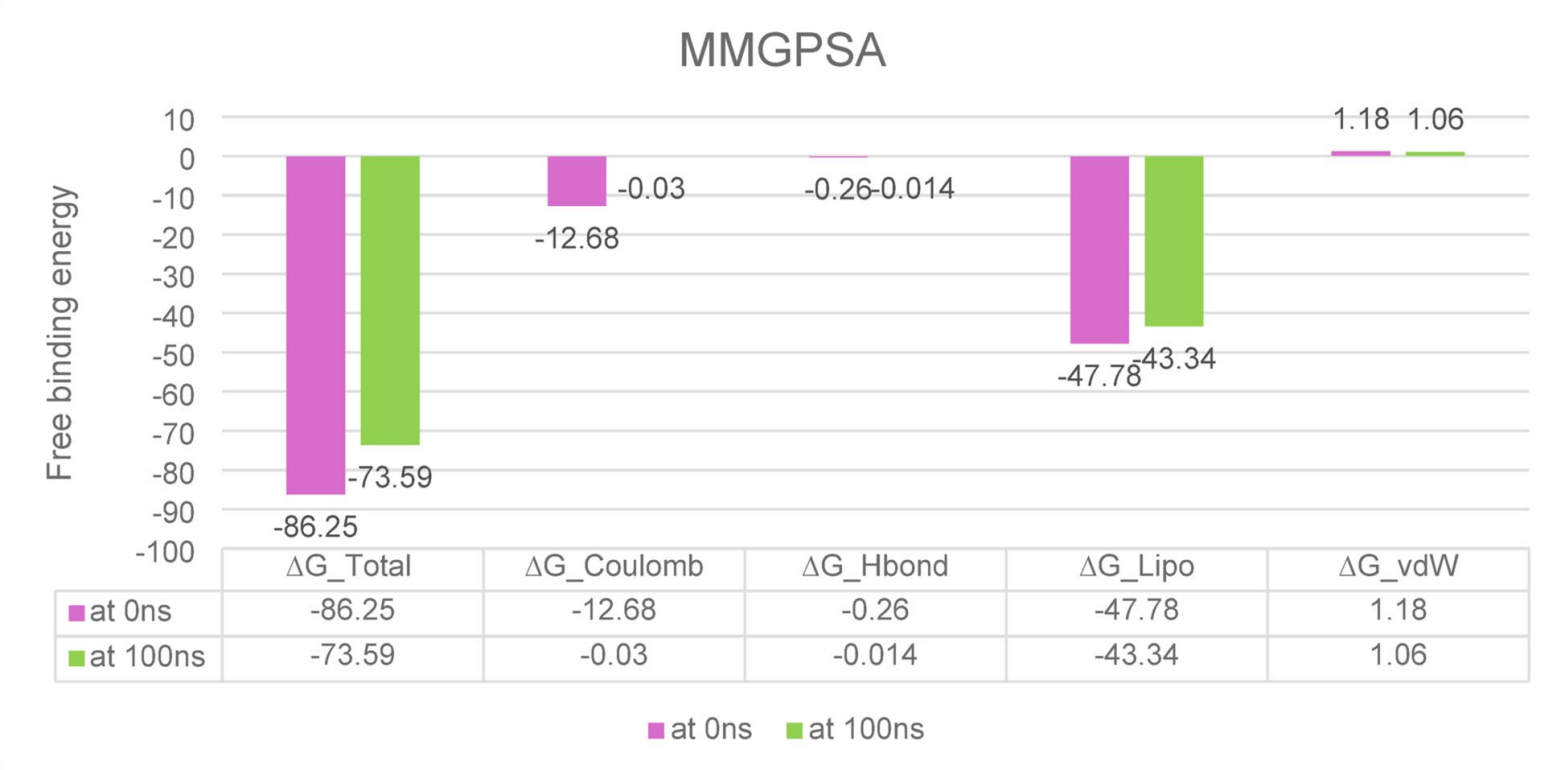
MM-GBSA energies for Compound **S2**_**M2**_ against **EGFR-TK** (kcal/mol).

The results observed in Figs. 13 and 14 provide an analysis of Compound **S2**_**M2**_ against CAIX and EGFR-TK. Throughout the simulation, Compound **S2**_**M2**_ maintained a consistent total binding free energy (∆G), with an initial value of − 59.48, and -86.25 kcal/mol at 0 ns, for CAIX and EGFR-TK, respectively, which remained stable at 100 ns. This persistence in binding energy demonstrates the sustained stability of the Compound **S2**_**M2**_ over the course of the simulation.

### Structure–activity relationship analysis

The semisynthetic modification of the parent glycoside **M**_**2**_ was designed to modulate the physicochemical and biological properties associated with its free hydroxyl groups. Polyhydroxylated glycosides are typically characterized by high polarity and extensive hydrogen-bonding capacity, which can limit membrane permeability and drug-like behavior^13,14^. To address these limitations, we employed a targeted esterification strategy that masks the hydroxyl functionalities and introduces an *α*,*β*-unsaturated carbonyl (enone) pharmacophore. This approach was intended to enhance lipophilicity and incorporate a Michael acceptor motif with reported anticancer relevance.

Our structure–activity relationship (SAR) analysis revealed that biological activity depended largely on scaffold modification and the incorporation of the *α*,*β*-unsaturated carbonyl group. Compared with the parent alcohol **M**_**2**_, both enone derivatives (**S1**_**M2**_ and **S2**_**M2**_) displayed improved cytotoxic activity, indicating that esterification significantly enhanced the biological profile. Notably, **S2**_**M2**_ was the most active compound, likely because the smaller crotonate enone fits more favorably within the EGFR-TK and CAIX active sites than the bulkier cinnamoyl group of **S1**_**M2**_. This structural advantage is consistent with the superior dual inhibitory activity observed for **S2**_**M2**_, which exhibited IC₅₀ values of 0.40 µM against EGFR-TK and 0.27 µM against CAIX, surpassing the reference inhibitor in the CAIX assay. While **M**_**3**_ also exhibited strong EGFR/CAIX inhibition, it showed only moderate cytotoxicity, suggesting that glycosylated phenolic scaffolds can retain target-specific activity without necessarily inducing high cell death. In addition, the glycosylated nature of these *Moringa*‑derived compounds may support prodrug behavior and improve aqueous solubility and selectivity, as documented for glycosylated anticancer agents^41^. These experimental observations are consistent with our docking and molecular dynamics results, which supported stable binding of **S2**_**M2**_ in both targets and confirmed its superior dual inhibitory profile. Collectively, these data validate our design strategy and identify the enone-modified **M**_**2**_ scaffold, particularly **S2**_**M2**_, as the most promising lead for further development.

## Conclusion

This study identifies *Moringa oleifera* pericarp as a promising source of bioactive compounds and suggests that **S2**_**M2**_ is the most active semi-synthetic sugar-based enone derivatives among the compounds investigated. The compound showed notable cytotoxicity, improved selectivity toward cancer cells, and dual inhibitory activity against EGFR and CAIX in vitro. These findings suggest that **S2**_**M2**_ may be a promising lead for further anticancer development, and underscore the value of sustainable plant-based approaches in anticancer lead discovery.

However, the present study is limited to in vitro cytotoxicity, enzyme inhibition assays, and computational analyses. Therefore, further investigations, including detailed mechanistic studies, expanded validation across diverse cancer models, and in vivo studies, are required to confirm therapeutic efficacy, selectivity, and safety.

## Experimental section

### Plant material

*Moringa oleifera* pericarp was collected as agro-waste from the Faculty of Pharmacy farm, Mansoura University, Dakahlia, Egypt, on 7 November 2021.

### Bio-guided isolation of active constituents from *M. oleifera* pericarp active fractions

The air-dried powdered pericarp of *M. oleifera* (5.0 kg) was extracted by maceration with methanol until exhaustion, concentrated to a syrupy consistency under reduced pressure, and dried to constant weight. The dried extract was fractionated in a separating funnel by successive extraction with petroleum ether, methylene chloride, and ethyl acetate. The ethyl acetate fraction was evaporated to dryness under reduced pressure and stored for further investigation.

The ethyl acetate fraction (12.8 g) was applied to the top of a silica gel column (75 × 3.5 cm, 300 g). The column was packed in methylene chloride and eluted with a gradient of methylene chloride–methanol; fractions were collected in 200 mL portions.

**Fractions 7–25** (1.1 g), eluted with methylene chloride–methanol (97.5:2.5), were collected and rechromatographed on a silica gel column (38 × 1.5 cm) using gradient elution with petroleum ether–ethyl acetate (100:0 → 70:30), collecting 15 mL fractions. Subfractions 29–55 were then obtained and further purified on a Sephadex LH-20 column (25 × 1.5 cm) using methanol under isocratic conditions. From subfractions 18–25, white interlacing crystals were obtained (Compound **M**_**1**_, 12 mg). The compound exhibited an *R*_*f*_ value of 0.28 on normal-phase TLC using petroleum ether–ethyl acetate (40:60) as the developing solvent system. The spot showed quenching under UV 254 nm and developed a light green coloration upon spraying with vanillin/H₂SO₄ reagent followed by heating at 105 °C for 1 min.

**Fractions 48–73** (1.5 g), eluted with methylene chloride–methanol (95:5), yielded a precipitate identified as Compound **M**_**2**_. TLC analysis revealed a major spot with an *R*_*f*_ value of 0.79 using methylene chloride–methanol (80:20). Compound **M**_**2**_ (280 mg) was obtained as colorless crystals. The spot showed quenching under UV 254 nm and developed a yellowish-green color upon spraying with vanillin/H₂SO₄ reagent followed by heating at 105 °C for 1 min.

**Fractions 86–102** (2 g) were rechromatographed over a silica gel column (38 × 1.5 cm) using gradient elution with ethyl acetate–methanol (100:0 → 60:40; fractions 1–120), collecting 15 mL fractions. The eluted fractions were monitored by TLC, and similar fractions were combined. Subfractions 36–55 (60 mg) showed a major spot and were further purified by preparative TLC to afford Compound **M**_**3**_ (8 mg). This spot showed quenching under UV 254 nm and developed a greyish-green color upon spraying with vanillin/H₂SO₄ reagent followed by heating at 105 °C for 1 min, with an *R*_*f*_ value of 0.36 (methylene chloride–methanol, 8:2).

### Chemistry

#### Isolated compounds

**4-(*α*-L-rhamnopyranosyloxy)-benzaldehyde (M**_**1**_**)** was isolated as colorless needles, m.p. (140.46 °C); *R*_*f*_ value of 0.28 (TLC, Pet.ether/EtOAc, 4:6); ^1^H- NMR (400 MHz, CDCl_3_) δ 9.93 (s), 7.88 (d, *J* = 8.56), 7.21 (d, *J* = 8.56), 5.65, 5.01, 4.3, 4.2, 3.7, 1.23 (d, *J* = 6.08); ^13^C-NMR (100 MHz, CDCl_3_) δ 191.0, 161.0, 155.7, 131.1, 116.4, 97.4, 73.1, 70.5, 69.1, 68.7, 17.5; IR (cm^− 1^) 3480 (OH stretching), 3333 (aldehydic O-H stretching), 2931 (C-H stretching), 1685 (carbonyl for aromatic aldehyde), and 1603 (aromatic C = C); GC-MS analysis appeared at Rt = 22 min. revealing a molecular ion peak at *m/z* 268.1 (M⁺), and the fragmentation pattern was characterized by prominent peaks at *m/z* 147 and *m/z* 121.02.

**4-(*α*-L-rhamnosyl) benzyl alcohol (M**_**2**_**)** was isolated as colorless needle crystals, m.p. ( 145.91 °C); *R*_*f*_ value of 0.79 TLC (methylene chloride/ methanol, 8:2); ^1^H- NMR (400 MHz, CD_3_OD) δ 7.30 (d, *J* = 8.56), 7.06 (d, *J* = 8.6), 4.56 (s), 5.44 (d, *J* = 1.08), ), 4.01(d, *J* = 1.24), 3.85 (dd, *J* = 3.24, 9.44), 3.63(m, ), 3.48(t, *J* = 9.48), 1.24(d, *J* = 6.2); ^13^C-NMR (100 MHz, CD_3_OD) δ 155.7, 135.0, 128,2, 116,0, 98.4, 72.5, 72.2, 70.8, 70.7, 63.4, 16.7; IR (cm^− 1^) 3327 (OH stretching), 2935(C-H stretching), 1623 (aromatic C = C); GC-MS analysis of compound **M**_**2**_ revealed a molecular ion peak at *m/z* 270.3 (M⁺), and the fragmentation pattern exhibited prominent peaks at *m/z* 146 and *m/z* 124.05.

**4-(hydroxymethyl) phenol-1-O-*β*-D-glucopyranosyl-(1''–3')-*O*-*α*-L-rhamnopyranosid (M**_**3**_**)** was isolated as amorphous powder, m.p ( 156.25 °C) ; *R*_*f*_ value of 0.36 TLC (methylene chloride/ methanol, 8:2); ^1^H- NMR (400 MHz, CDCl_3_) δ 6.94 (d, *J* = 8.6), 7.81 (d, *J* = 8.56), 5.43 (d, *J* = 1.08), 5.34, 4.44 (s), 4.2 (d, J = 1.24), 3.87 (dd, *J* = 3.24, 9.44), 3.65 (m), 3.48 (t, *J* = 9.48), 3.66, 3.53, δ 3.50, 3.32, 1.13(d, *J* = 6.2); ^13^C-NMR (100 MHz, CDCl_3_) δ 155.6, 135.2, 128.3, 116.1, 104, 98.2, 81.4, 76.3, 74.0, 72.4, 69.6, 63.3, 60.9, 16.7. The molecular formula of **M**_**3**_ was determined as C₁₉H₂₈O₁₁ based on HR-ESI-MS, which showed a peak at *m/z* 431.1559 [M–H]⁻ (calculated for [M–H]⁻: 431.15588). The MS² fragmentation of **M**_**3**_ exhibited characteristic ions at *m/z* 307.1040 [M – C₇H₈O₂ – H]⁻, 179.0561, and 145.0506.

### Semi-synthesis of enone derivatives from isolated compound M_2_

To a solution of alcohol (0.1 g, 0.2 mmol, 1 eq.), DMAP (0.048 g, 0.2 mmol, 1 eq.), and cinnamic acid or crotonic acid (1.2 mmol, 6 eq.) in anhydrous CH_2_Cl_2_ (8 mL), DCC (0.49 g, 1.2 mmol, 6 eq.) was added at 0 °C. The resulting mixture was stirred at room temperature until the starting material was not detected by TLC (12–24 h). Then, the reaction mixture was filtered, and the residue was washed with CH_2_Cl_2_ (2 × 10 mL). The CH_2_Cl_2_ solution was washed with 5% HCl (3 × 30 mL), saturated NaHCO_3_ (3 × 30 mL) and saturated NaCl (3 × 30 mL), respectively. The organic layer was dried over anhydrous Na_2_SO_4_ and concentrated to dryness under vacuum to give the pure crystals in good yield^37^.

**4-(α-L-rhamnosyloxy) benzyl tetracinnamate derivative (S1**_**M2**_**)** was isolated as colorless crystals, Yield = 75%, m.p (145.3 °C); *Rf* value of 0.75 TLC (pet.ether /EtOAc, 7:3); ^1^H- NMR (400 MHz, CDCl_3_) δ 7.77, 7.68, 7.60, 7.40, 7.33, 6.79–6.75 (H3, d, *J* = 16 Hz), 6.68–6.64 (H2, d, *J* = 16 Hz), 5.23, 4.14, 3.91, 3.78, 3.50, 1.32; ^13^C-NMR (100 MHz, CDCl_3_) δ 166.92, 166.05, 165.95, 165.9, 156.3, 146.5, 146.1, 146.0, 145.1, 135.2, 130.1, 128.6, 128.3, 128.2, 128.1, 127.9, 118, 117.04, 117.1, 116.5, 95.9, 71.2, 70.1, 69.1, 67.6, 66, 17.8; The IR **C = O stretching vibrations** at 1708 cm⁻¹, and the disappearance of hydroxyl **O–H stretches** typically observed at 3200–3500 cm⁻¹; The molecular formula of **S1**_**M2**_ was determined as **C₄₉H₄₂O₁₀** based on its HR-ESI-MS¹ peak at *m/z* [M + H]^+^ 791.4240.

**4-(α-L-rhamnosyl) benzyl tetra crotonate derivative (S2**_**M2**_**)** was isolated as feathery crystals, Yield = 65%, m.p (158.5 °C); R*f* value of 0.81 (TLC, pet.ether /EtOAc, 7:3); ^1^H- NMR (400 MHz, CDCl_3_) δ 7.084–7.106 ( *J* = 8.8), 7.319–7.340 (*J* = 8.4), 6.9–7.1, 6.9–7.1, δ 5.1 (s), 5.517, δ 4.014–4.070, 4.233–4.215 (*J* = 7.2), 3.267–3.273 (*J* = 2.4), 3,454-3.546, 3.686–3.766, 3.953–3.999, 1.269 (s), 1.76 (s), 1.9, δ 7.34 (s), 5.9–6.0 (*J* = 14.9 Hz);^13^C-NMR (100 MHz, CDCl_3_) δ 166.38,165.46, 165.31, 165.28, 155.9, 146.6, 146.2, 146.1, 145, 129.9, 130.3, 129.9, 124.0, 122.5, 121.9, 121.8, 116.4, 95.7, 70,7, 68.4, 65.6, 18.3, 18.2, 18.1, 18.0, 17.5. The molecular formula of **S2**_**M2**_ was determined as **C₂₉H₃₄O₁₀** with exact mass *m/z* 542.21520 based on its peak at *m/z* 565.3 [M + Na]⁺.

## Biological evaluation

### Antioxidant screening (ABTS assay)

The experiment was performed in triplicate according to Lissi et al.^42^. The absorbance of the control (ABTS radical solution), which exhibited a green-blue color, was recorded at λmax = 734 nm.

### Antimicrobial screening

The procedure was performed according to Abdel Rahman et al.^43^. The activity index was determined by comparing the inhibition zone of the extract with that of a reference antibiotic using the following equation:

Activity index = (the inhibition zone of the extract/ the inhibition zone of the standard) x 100.

### Cytotoxic activity (MTT assay)

#### Cell culture

Human cancer cell lines (HepG2, and HCT116) and normal human lung fibroblast cells (WI-38) were obtained from the American Type Culture Collection (ATCC). Cells were cultured in Dulbecco’s Modified Eagle Medium (DMEM; Invitrogen/Life Technologies) supplemented with 10% fetal bovine serum (FBS; Hyclone), 10 µg/mL insulin (Sigma-Aldrich), and 1% penicillin–streptomycin. Cells were maintained at 37 °C in a humidified atmosphere containing 5% CO₂.

#### MTT cytotoxicity assay

The cytotoxic activity of the tested compounds was evaluated using the MTT assay commercial kit (Sigma-Aldrich, TOX-1). Cells were seeded in 96-well plates at a density of 1.2–1.8 × 10⁴ cells per well in 100 µL of complete growth medium and allowed to attach for 24 h. Subsequently, cells were treated with serial concentrations of the tested compounds (100, 25, 6.3, 1.6, and 0.4 µg/mL) and incubated for 48 h at 37 °C. Following treatment, MTT solution was prepared according to the manufacturer’s instructions and added to each well at a final volume corresponding to 10% of the culture medium. Cells were incubated for 2–4 h to allow the formation of insoluble purple formazan crystals. The medium was then carefully removed, and the formazan crystals were dissolved using solubilization solution (10% Triton X-100 in 0.1 N HCl in isopropanol).

Absorbance was measured at 570 nm with a reference wavelength of 690 nm using a microplate reader. Cell viability was expressed as a percentage relative to untreated control cells. All experiments were performed in triplicate.

#### Determination of IC₅₀ and selectivity index

The half-maximal inhibitory concentration (IC₅₀) values were determined from dose–response curves by plotting the percentage of cell viability against the logarithm of compound concentrations. The selectivity index (SI) was calculated via the equation below: SI equals IC_50_ normal / IC_50_ cancer, where IC₅₀ (normal) represents the concentration required to reduce the viability of normal cells by 50%, and IC₅₀ (cancer) represents the concentration required to reduce the viability of cancer cells by 50%^44,45^.

### CAIX activity

Carbonic anhydrases are zinc enzymes present in both prokaryotes and eukaryotes. They efficiently catalyze the reversible hydration of CO_2_ to bicarbonate. Carbonic anhydrase (CA) activity was determined by the spectrophotometric method. It utilizes the esterase activity of an active CA on an ester substrate, which releases a chromogenic product. The released product can be easily quantified using an absorbance microplate reader. Enzyme assay was performed by preparing specific reaction mixtures for experimental conditions, including Background Control (BC), Enzyme Control (EC), Test Samples (S), Solvent Control (SC), and Inhibitor Control (IC). For each well, CAIX Assay Buffer and CAIX Enzyme were added as follows: 85 µL of buffer to BC, 90 µL of buffer and 5 µL of enzyme to EC, and 80 µL of buffer with 5 µL of enzyme to S, SC, and IC. All solutions were mixed well before use. Candidate inhibitors were prepared at ten times the final concentration using a suitable solvent, such as DMSO. Then, 10 µL of each test inhibitor was added to the corresponding test wells. Solvent-only controls received 10 µL of the inhibitor solvent, while IC and BC wells were given 10 µL of a known CA inhibitor (2 mM Acetazolamide). The plate was incubated at room temperature for 10 min. To reduce the chance of false-negative results from compound interference, each test compound was evaluated alongside its background control. After incubation, 5 µL of CAIX substrate was added to each well. Absorbance was measured at 405 nm in kinetic mode for 60 min at room temperature. Enzymatic activity was quantified by selecting two time points (t₁ and t₂) within the linear range of the kinetic curve and calculating the rate of change in absorbance^46^.

### EGFR-TK activity

The epidermal growth factor receptor is the cell-surface receptor for members of the epidermal growth factor family. Overexpression and/or hyperactivation of EGFR-TK are associated with several human cancers such as lung, glioblastoma, and epithelial tumors of the neck and head, leading to the development of anticancer therapeutics targeting EGFR. Kinase assay reagents, including 5× Kinase Assay Buffer, ATP, and 50× PTK substrate, were thawed to room temperature before setting up the assay. A reaction master mix was prepared, with each well requiring 25 µL. Recombinant EGFR enzyme was thawed on ice and briefly centrifuged to collect its contents. The enzyme was then diluted to a final working concentration of 1 ng/µL using 1× Kinase Assay Buffer, depending on the necessary number of reactions. To start the kinase reaction, 20 µL of the diluted EGFR enzyme was added to the wells. The plate was incubated at 30 °C for 40 min. After incubation, Kinase-Glo Max reagent was thawed to room temperature. At the end of the reaction, 50 µL of the reagent was added to each well. The plate was then covered with aluminum foil to shield it from light and incubated at room temperature for 15 min to allow the luminescent signal to develop completely. Luminescence was then measured using a microplate reader based on the manufacturer’s recommended settings^47^.

### Molecular docking

The molecular docking was processed to evaluate the possible affinity of the tested compounds against CAIX and EGFR TK. The target protein (code: 6s03 and 4hjo) was obtained from the protein data bank^48^.^49^. At first, water molecules were removed from the complexes. Next, preparation options were used to prepare, correct crystallographic disorders and unfilled valence atoms. Protein structure energy was minimized by applying CHARMM force fields. Using Chem-Bio Draw Ultra17.0, 2D structures of tested compounds were drawn and saved as SDF files, the saved files were opened, 3D structures were protonated, and 0.1 RMSD kcal/mole energy was minimized by the MMFF94 force field. Then, the minimized structures were prepared for docking via the ligand preparation tools. The docking process was carried out through the docking option using Autodock Vina 1.5.7 software^50^. The receptor was held rigid while the ligands were allowed to be flexible. During the refinement, each molecule was allowed to produce twenty different poses with the proteins. Then docking scores (affinity energy) of the best-fitted poses with the active sites were recorded and 3D figures were generated by the Discovery Studio 2016 visualizer^51^.

### Molecular dynamics (MD) simulation

Molecular dynamics simulations were conducted utilizing the Desmond software suite (Schrödinger LLC)^52^. All simulations were carried out in the isothermal-isobaric (NPT) ensemble, maintaining a temperature of 300 K and a pressure of 1 bar. Each simulation was run for a total of 100 ns, with a relaxation time of 1 ps applied to the investigated ligands. The OPLS_2005 force field was used throughout to parameterize the systems. Long-range electrostatic interactions were treated with the particle mesh Ewald method, employing a 9.0 Å cut off for Coulombic interactions^53^.

Water molecules were modeled explicitly using the simple point charge (SPC) model. Pressure and temperature were regulated using the Martyna–Tuckerman–Klein chain coupling scheme (coupling constant of 2.0 ps) and the Nosé–Hoover chain coupling scheme, respectively. The r-RESPA multiple time-step integrator was employed to compute nonbonded interactions, updating short-range forces at every step and long-range forces every three steps. Trajectories were recorded at 4.8 ps intervals for subsequent analysis.

The binding pattern and interactions between the ligand and target protein were analyzed using the Simulation Interaction Diagram tool available in the Desmond MD package. To evaluate the stability of the molecular dynamics simulations, the root mean square deviation (RMSD) of the ligand and protein atomic positions was monitored throughout the simulation time^54^.

### Statistical analysis

Statistical analyses were performed using GraphPad Prism (version 10.3.1). Data are presented as mean ± standard deviation (SD). All experiments were conducted in triplicate, and the reported values represent the average of three independent determinations.

## Supplementary Information

Below is the link to the electronic supplementary material.

Supplementary Material 1

## Data Availability

Included in the paper or Supplementary Information.
