# Supplementary material for: Valorization of Moringa oleifera pericarp via semi-synthetic sugar-based enone derivatives with anticancer potential: phytochemical isolation, cytotoxic evaluation, and dual EGFR/CAIX targeting
Source: Sci Rep. 2026 Jul 4;16:20557. doi: 10.1038/s41598-026-59686-2 (PMC13333019; doi:10.1038/s41598-026-59686-2)
Supplement: Supplementary file 1 — Supplementary Material 1 [file 41598_2026_59686_MOESM1_ESM.docx]

**Valorization of *Moringa oleifera* Pericarp via Semi-Synthetic Sugar-Based Enone Derivatives with Anticancer Potential: Phytochemical Isolation, Cytotoxic Evaluation, and Dual EGFR/CAIX Targeting**

Mayye Majed ^1^, Amal A. Galala ^1,2^, Dina I. A. Othman^3^, Mohamed M. Amer ^1^, and Sara Abouzeid^1^*

^1^Pharmacognosy Department, Faculty of Pharmacy, Mansoura University, Mansoura 35516, Egyp.

^2^Pharmacognosy Department, Faculty of Pharmacy, Horus University in Egypt (HUE),
New Damietta 34517, Egypt

^3^Department of Pharmaceutical Organic Chemistry, Faculty of Pharmacy, Mansoura University, Mansoura 35516, Egypt.

*****Correspondence: [sara86@mans.edu.eg](mailto:sara86@mans.edu.eg) (S.A.)

**List of tables:**

|  |  | **Page No.** |
| --- | --- | --- |
| **S1** | Results of the antioxidant activity screening of the selected waste fractions using ABTS assay | **4** |
| **S2** | Results of antimicrobial activity screening of fractions from *M. oleifera fractions* | **4** |
| **S3** | ^1^H-NMR and APT spectral data of compound **M_1_** compared to published data of compound M_1_ | **5** |
| **S4** | ^1 1^H-NMR and APT spectral data of compound **M_2_** compared to published data | **6** |
| **S5** | ^1^H-NMR and APT spectral data of compound **M_3_** compared to published data | **7** |
| **S6** | ^1^H-NMR and APT spectral data of semisynthetic compound **S1_M2_** | **8** |
| **S7** | ^1^H-NMR and APT spectral data of semisynthetic compound **S2_M2_** | **9** |
| **S8** | H-NMR and APT spectral data of semisynthetic compound **S2_M2_**  The chemical shifts δ are expressed in ppm and coupling constants *J* in Hz. | **10** |
| **S9** | HMBC correlations of compound **S2_M2_** deduced from HMBC spectrum | **11** |
| **S10** | Absorbance of total methanolic extract and different fractions of *Moringa oleifera* in MTT assay against HCT116 cancer cell lines. | **12** |
| **S11** | % viability and IC_50_ of total methanolic extract and fractions of *Moringa oleifera* in MTT assay against HCT116 cancer cell line. | **13** |
| **S12** | Absorbance of total methanolic extract and different fractions of *Moringa oleifera* in MTT assay against HepG2 cell lines. | **14** |
| **S13** | % viability and IC_50_ of total methanolic extract and fractions of *Moringa oleifera* in MTT assay against HepG2 cancer cell line. | **15** |
| **S14** | Absorbance of isolated and semisynthetic compound of *Moringa oleifera* in MTT assay against HCT116 cancer cell lines | **16** |
| **S15** | viability and IC_50_ of isolated and semisynthetic compound of *Moringa oleifera* in MTT assay against HCT116 cancer cell line | **17** |
| **S16** | Absorbance of isolated and semisynthetic compound of Moringa *oleifera* in MTT assay against HepG2 cancer cell lines | **18** |
| **S17** | % viability and IC_50_ of isolated and semisynthetic compound of *Moringa oleifera* in MTT assay against HepG2 cancer cell lines | **19** |
| **S18** | Absorbance of isolated and semisynthetic compound of *Moringa oleifera* in MTT assay against WI38 cancer cell lines | **20** |
| **S19** | % viability and IC_50_ of isolated and semisynthetic compound of *Moringa oleifera* in MTT assay against WI38 cancer cell lines | **21** |
| **S20** | IC50 and selectivity index of isolated compound M_2_ and semisynthetic compounds | **21** |
| **S21** | % inhibition of isolated and semi-synthesized compounds against EGFR TK enzyme inhibition. All data are presented as mean value ± SD for three independent experiments | **22** |
| **S22** | % inhibition of isolated and semi-synthesized compounds against CAIX enzyme inhibition. All data are presented as mean value ± SD for three independent experiments | **23** |
| **S23** | IC_50_ values of isolated and semi-synthesized compounds for EGFR TK and CAIX enzymes inhibition with µg/mL and µM | **24** |

**List of figures:**

| **Fig. No.** | |  | **Page** |
| --- | --- | --- | --- |
| **S1** | GC-MS spectrum and fragmentation of compound **M_1_** | | **24** |
| **S2** | IR spectrum of compound **M_1_** | | **25** |
| **S3** | ^1^H spectrum (CDCl_3_, 400 MHz) of compound **M_1_** | | **25** |
| **S4** | APT of compound **M_1_**, (CDCl_3_ ( | | **26** |
| **S5** | GC-MS spectrum and fragmentation of compound | | **26** |
| **S6** | IR spectrum of compound **M_2_** | | **27** |
| **S7** | ^1^H-NMR spectrum of compound **M_2_** | | **27** |
| **S8** | APT-NMR spectrum of compound **M_2_** | | **28** |
| **S9** | HR-ESI-MS chromatogram of compound **M_3_** in negative ion mode.  A) TIC chromatogram of compound **M_3_** at R_t_ 6-9  B) MS^2^ fragment of compound **M_3_** | | **28** |
| **S10** | ^1^H-NMR spectrum of compound **M_3_** | | **29** |
| **S11** | APT-NMR spectrum of compound **M_3_** | | **29** |
| **S12** | HR-ESI mass of compound **S1_M2_** | | **30** |
| **S13** | IR spectrum of compound **S**1**_M_**_2_ | | **31** |
| **S14** | ^1^H-NMR spectrum of compound **S**1**_M_**_2_ | | **31** |
| **S15** | APT spectrum of compound **S**1**_M_**_2_ | | **32** |
| **S16** | HMBC-NMR spectrum of compound **S**1**_M_**_2_ | | **32** |
| **S17** | LC MS of compound **S2_M2_** | | **33** |
| **S18** | ^1^H-NMR spectrum of compound **S2_M2_** | | **34** |
| **S19** | APT-NMR of compound **S2_M2_** spectrum | | **34** |
| **S20** | HMBC spectrum of compound **S2_M2_** | | **35** |

**Table S1: Antioxidant activity screening of *Moringa oleifera* Pericarp fractions using ABTS assay (1mg/ml). All data are presented as mean value ± SD for three independent experiments**

| **Fractions** | **% inhibition** |
| --- | --- |
| **Petroleum ether extract** | 23.5± 0.01% |
| **Methylene chloride extract** | 42.9± 0.03% |
| **Ethyl acetate extract** | 68.7±0.04% |
| **Ascorbic-acid** | 88.1±0.1% |

**Table S2: Antimicrobial activity screening of fractions of *Moringa oleifera* Pericarp (1mg/ml)**

| Fractions | *E. coli* | | *S. aureus* | | | *C. Albicans* | |
| --- | --- | --- | --- | --- | --- | --- | --- |
|  | Diameter of inhibition zone  (mm) | % Activity index | Diameter of inhibition zone  (mm) | | % Activity index | Diameter of  inhibition zone  (mm) | % Activity index |
| *M. oleifera* methylene chloride extract | 8.17±0.15 | 33.3 | 10.17±0.15 | | 43.5 | 11.1±0.1 | 42.3 |
| *M. oleifera* ethyl acetate extract | **20.2**±0.2 | **83.3** | **18.17**±0.15 | | **78.3** | **15.1**±0.1 | **57.7** |
| *M. oleifera* petroleum ether extract | NA | ---- | NA | | ---- | 3.06±0.05 | 11.5 |
| Ampicillin | 24.2±0.2 | 100 | 23±0.2 | 100 | | NA | ---- |
| Colitrimazole | NA | ---- | NA | ---- | | 26.17±0.15 | 100 |

*NA: no activity, *E. coli: Escherichia coli, P. aeuruginosa: Pseudomonas aeuruginosa, S. aureus*: *Staphylococcus aureus, C. albicans: Candida albicans.* All data are presented as mean value ± SD for three independent experiments

**Chemistry**

**Compound M_1_**

4-(α-L-rhamnopyranosyloxy)- benzaldehyde

**Table S3: ^1^H-NMR and APT spectral data of compound M_1_ compared to published data of compound M_1_**

| **C/H**  **No.** | **Compound M_1_** | | | **^4-(α-L-rhamnopyranosyloxy)- benzaldehyde^** | |
| --- | --- | --- | --- | --- | --- |
|  | **^1^H-NMR*** | **APT** |  | **^1^H*** | **^13^C*** |
| 1 | - | 155.7 | C | - | 160.4 |
| 2 | 7.21 (d, *J*=8.56) | 116.4 | CH | 7.16 | 116.5 |
| 3 | 7.88 (d, *J*=8.56) | 131.1 | CH | 7.81 | 131.8 |
| 4 | - | 161.0 | C | - | 131.6 |
| 5 | 7.88(d, *J*=8.56) | 131.1 | CH | 7.81 | 131.8 |
| 6 | 7.21 (d, *J*=8.56) | 116.4 | CH | 7.16 | 116.5 |
| 7 | 9.93 (s) | 191.0 | CHO | 9.86(s) | 191.5 |
| Sugar moiety (Rhamnose) (O- glycosidic linkage) | | | | |  |
| 1´ | 5.65 | 97.4 | CH | 5.53 d | 95.5 |
| 2´ | 4.2 | 73.1 | CH | 5.39, dd | 70.7 |
| 3´ | 4.3 | 70.5 | CH | 5.45, dd | 69.4 |
| 4´ | 5.01 | 69.1 | CH | 5.12, t | 68.7 |
| 5´ | 3.7 | 68.7 | CH | 3.88, dq | 67.6 |
| 6’ | 1.23(d, *J*=6.08) | 17.5 | CH3 | 1.15, d | 17.1 |

*The chemical shifts δ are expressed in ppm and coupling constants *J* in Hz.

^13^C and ^1^H NMR are measured in CDCl3 at 400 MHz.

**Published data,^13^C and ^1^H-NMR are measured in CDCl3- at MHz**(Leuck, M., & Kunz, H. (1998).)**

**Compound M_2_**

**4-(α-L-rhamnosyl) benzyl alcohol**

**Table. S4 : ^1^H-NMR and APT spectral data of compound M2 compared to published data**

| **C/H**  **No.** | **Compound M2** | | | ****4-(α-L-rhamnosyl) benzyl alcohol** | |
| --- | --- | --- | --- | --- | --- |
|  | **^1^H*** | **13C*** | **APT** | **^1^H**** | **^13^C**** |
| 1 | - | 155.7 | C | - | 155.3 |
| 2 | 7.30 (d, *J*=8.56) | 116,0 | CH | 7.22 (*J*=8.20) | 116.6 |
| 3 | 7.06 (d, *J*=8.6) | 128,2 | CH | 6.96 (*J*=8,20) | 128.4 |
| 4 | - | 135.0 | C | - | 136.2 |
| 5 | 7.06 (d, *J*=8.6) | 128.2 | CH | 6.96 (*J*=8,20) | 129.7 |
| 6 | 7.30 (d, *J*=8.56) | 116.0 | CH | 7.22 (*J*=8.20) | 117.9 |
| 7 | 4.56 (s) | 63.4 | CH_2_ | 4.73(d, *J*=4.73) | 62.9 |
| Sugar moiety (Rhamnose) (O- glycosidic linkage) | | | | | |
| 1^’^´ | 5.44 (d, *J*=1.08) | 98.4 | CH | 5.32(d, *J*=1.32) | 98.8 |
| 2´ | 4.01  (d, *J*=1.24) | 72.5 | CH | 3.61 | 70.8 |
| 3´ | 3.85  (dd, *J*=3.24, 9.44) | 70.8 | CH | 3.43 | 70.6 |
| 4´ | 3.48 (t, *J*=9.48) | 72.2 | CH | 3.62 | 72.2 |
| 5´ | 3.63(m) | 70.7 | CH | 3.25 | 69.7 |
| 6´ | 1.24(d, *J*=6.2) | 16.7 | CH3 | 1.07 (d, *J*=6.14) | 18.2 |

*The chemical shifts δ are expressed in ppm and coupling constants *J* in Hz.

^13^C and ^1^H NMR are measured in CD3OD at 400 MHz.

**Published data (Yadessa *et al*,.2017 ) compound M 2,^13^C and ^1^H-NMR are measured in DMSO-d6 at 400 MHz

**Table S5 :^1^H-NMR and APT spectral data of compound M3 compared to published data**

| **C/H No.** | **13C*** | **^1^H*** | **APT** | **^13^C**** | **^1^H**** |
| --- | --- | --- | --- | --- | --- |
| 1 | 155.6 | - | C | 157.1 | - |
| 2,6 | 116,1 | 7.81 (d, *J*=8.56) | CH | 118.8 | 7.36 (*J*=8.20) |
| 3,5 | 128,3 | 6.94 (d, *J*=8.6) | CH | 130.6 | 6.96 (*J*=8,20) |
| 4 | 135.2 | - | C | 130.6 | - |
| 7 | 60.9 | 4.44 (s) | CH2 | 44.0 | 4.23 |
| Sugar moiety (Rhamnose) (O- glycosidic linkage) | | | | | |
| 1´ | 98.2 | 5.43 (d, *J*=1.08) | CH | 93.7 | 5.44(d, *J*=3.2) |

| 2´ | 81.4 | 4.2  (d, *J*=1.24) | CH | 79.4 | 4.14 |
| --- | --- | --- | --- | --- | --- |
| 3´ | 71.2 | 3.87  (dd, *J*=3.24, 9.44) | CH | 71.3 | 4.11 |
| 4´ | 74.0 | 3.48  (t, *J*=9.48) | CH | 75.4 | 4.60 |
| 5’ | 69,6 | 3.65(m) | CH | 69.1 | 3.76(m) |
| 6’ | 16.7 | 1.13(d, *J*=6.2) | CH3 | 18.2 | 1.26  (d, *J*=6.00) |
| Sugar Glucose | | | | | |
| 1´´ | 104 | 5.34 | CH | 99.8 | 5.18  (*d*, 4.00 Hz) |
| 2´´ | 81.4 | 3.66 | CH | 82.5 | 3.62(m) |
| 3´´ | 76.3 | 3.50 | CH | 78.0 | 3.48-3.50 |
| 4´´ | 69.6 | 3.32 | CH | 69.5 | 3.33-3.47 |
| 5´´ | 72.4 | 3.53 | CH | 72.8 | 3.56-3.60 |
| 6´´ | 63.3 | 3.50 | CH3 | 62.0 | 3.49-3.51 |

*The chemical shifts δ are expressed in ppm and coupling constants *J* in Hz.

^13^C and ^1^H NMR are measured in CDCl3 at 400 MHz. **Published data (Xiong *et al.,* 2022)) ,^13^C and ^1^H-

NMR are measured in CDCl3- at MHz **(Xiong *et al.,* 2022).**

**Table S6 : ^1^H-NMR and APT spectral data of semisynthetic compound S1_M2_**

|  | **Product S1M_1_** | | |
| --- | --- | --- | --- |
| **C/H No.** | **^1^H** | **APT** | |
| 1 | - | 156.3 | C |
| 2 | 7.68 | 116.5 | CH |
| 3 | 7.77 | 128.1 | CH |
| 4 | - | 130.1 | C |
| 5 | 7.77 | 128.3 | CH |
| 6 | 7.68 | 116.5 | CH |
| 7 | - | 66 | CH_2_ |
| Sugar moiety (Rhamnose) (O- glycosidic linkage) | | | |
| 1´ | 5.23 | 95.9 | CH |
| 2´ | 4.14 | 71.2 | CH |
| 3´ | 3.91 | 70.1 | CH |
| 4´ | 3.78 | 69.1 | CH |
| 5´ | 3.50 | 67.6 | CH |
| 6´ | 1.32 | 17.8 | CH_3_ |
| Cinnamic ester moiety | | | |
| 1´´ | - | 165.9, 165.95, 166.05 and 166.92 | C |
| 2´´ | δ 6.68–6.64(H2, d, *J* = 16 Hz) | 116.5, 117.04, 117.1 and 118 | CH |
| 3´´ | δ 6.79–6.75 (H3, d, *J* = 16 Hz) | 145.1, 146.0, 146.1 and 146.5 | CH |
| 4´´ | - | 135.2 | CH |
| 5´´ | 7.60 | 128.2 | CH |
| 6´´ | 7.40 | 128.6 | CH |
| 7´´ | 7.33 | 127.9 | CH |
| 8´´ | 7.40 | 128.6 | CH |
| 9´´ | 7.60 | 128.2 | CH |

The chemical shifts δ are expressed in ppm and coupling constants J in Hz.13C and 1H NMR are measured in CDCl3 at 100 and 400 MHz respictively

**Table S7: HMBC correlations of compound S2_M2_ deduced from HMBC spectrum**

| **Proton** | **Proton**  **(Values in ppm)** | **HMBC Correlated Carbon (s) (**δ**Values in ppm)** |
| --- | --- | --- |
| H-1´ | 5.23 | 95.9 (C-1´), 128.6 (C- 6´´,8´´) |
| H-6´ | 1.33 | 67.6 (C-5´), 70.1 (C-3´) |
| H-2, 6 | 7.68 | 116.5 (C-2,6) |
| H-3, 5 | 7.77 | 156.3 (C-1), 95.9 (C-1´) |

|  |
| --- |

**Table** S8 **:** H-NMR and APT spectral data of semisynthetic compound **S2_M2_**

| **C/H No.** | **Product S2M_2_** | | |
| --- | --- | --- | --- |
|  | **^1^H** | **APT** | |
| 1 | - | 155.9 | C |
| 2 | 6.9-7.1 | 116.4 | CH |
| 3 | 7.084-7.106 *J*=8.8 | 129.9 | CH |
| 4 | 7.319-7.340 *J*=8.4 | 130.3 | C |
| 5 | - | 129.9 | CH |
| 6 | 6.9-7.1 | 116.4 | CH |
| 7 | 5.1 singlet | 65.6 | CH_2_ |
| Sugar moiety (Rhamnose) (O- glycosidic linkage) | | | |
| 1´ | 5.517 | **95.7** | CH |
| 2´ | 4.014-4.070 Range of doublets  4.233-4.215 *J*=7.2 | 65.6 | CH |
| 3´ | 3.267-3.273 doublet *J*=2.4 | 68.4 | CH |
| 4´ | 3,454-3.546 Range of multiplate | 69.6 | CH |
| 5´ | 3.686-3.766 multiplate  3.953-3.999 multiplate | 70,7 | CH |
| 6´ | 1.269 single | 17.5 | CH_3_ |
| Crotonate ester moiety | | | |
| 1´´ | 1.76 singlet  1.9 range of singlets | 18.0, 18.1, 18.2 and 18.3 | CH_3_ |
| 2´´ | 7.34  singlets | 145, 146.1, 146.2 and146.6 | CH_2_ |
| 3´´ | δ 5.9–6.0 range of doublets (*J*=14.9 Hz) | 121.8, 121.9, 122.5 and 124.0 | CH_2_ |
| 4´´ | - | 165.28, 165.31, 165.46 and 166.38 | C |

The chemical shifts δ are expressed in ppm and coupling constants *J* in Hz.

^13^C and ^1^H NMR are measured in CDCl_3_ at 100 and 400 MHz respictively

**Table S9: HMBC correlations of compound S2_M2_ deduced from HMBC spectrum**

| **Proton** | **Proton**  **(Values in ppm)** | **HMBC Correlated Carbon (s) (**δ**Values in ppm)** |
| --- | --- | --- |
| H-1´ | 5.517 | 116.4 (C-2,6) 166.4 (C- 4´´) |
| H-3´´ | 5.7-6.0 | 155.9 (C-1)166.4 (C-4´´) |
| H-3,5 | 7.084-7.106 | 116.4 (C-2,6) , 155.9 (C-1) |
| H-2, 6 | 6.9-7.1 | 155.9 (C-1), 95.7 (C-1´),116.4 (C-2,6) |

**Table S10**: Absorbance of total methanolic extract and different fractions of *Moringa oleifera* in MTT assay against HCT116 cancer cell lines.

| **HCT116** | | | | | | | | |
| --- | --- | --- | --- | --- | --- | --- | --- | --- |
| **Staurosporine** | Absorbance | 100ug | 25ug | 6.3ug | 1.6ug | 0.4ug | Blank | cc |
|  | A | 0.161 | 0.231 | 0.276 | 0.351 | 0.434 | 0.001 | 0.571 |
|  | B | 0.147 | 0.228 | 0.283 | 0.337 | 0.428 | 0.002 | 0.544 |
|  | C | 0.161 | 0.215 | 0.281 | 0.349 | 0.441 | 0.001 | 0.551 |
|  | mean | 0.15633 | 0.22467 | 0.28 | 0.34567 | 0.43433 | 0.001 | 0.555 |
|  | % viability | 28.1513 | 40.4562 | 50.4202 | 62.2449 | 78.2113 |  | |
| **Total methanolic extract** | A | 0.222 | 0.291 | 0.331 | 0.394 | 0.451 | 0.003 | 0.618 |
|  | B | 0.231 | 0.269 | 0.324 | 0.404 | 0.465 | 0.001 | 0.595 |
|  | C | 0.225 | 0.274 | 0.326 | 0.388 | 0.438 | 0.001 | 0.627 |
|  | mean | 0.226 | 0.278 | 0.327 | 0.39533 | 0.45133 | 0.002 | 0.613 |
|  | % viability | 36.8478 | 45.3261 | 53.3152 | 64.4565 | 73.587 |  | |
| **Ethyl acetate fraction** | A | 0.185 | 0.241 | 0.267 | 0.331 | 0.385 | 0.001 | 0.606 |
|  | B | 0.173 | 0.238 | 0.285 | 0.328 | 0.401 | 0.003 | 0.587 |
|  | C | 0.179 | 0.229 | 0.269 | 0.341 | 0.387 | 0.001 | 0.601 |
|  | mean | 0.179 | 0.236 | 0.2737 | 0.3333 | 0.391 | 0.002 | 0.598 |
|  | % viability | 29.9331 | 39.465 | 45.764 | 55.741 | 65.385 |  | |
| **Methylene chloride fraction** | A | 0.183 | 0.231 | 0.279 | 0.344 | 0.422 | 0.001 | 0.571 |
|  | B | 0.179 | 0.225 | 0.283 | 0.361 | 0.439 | 0.002 | 0.544 |
|  | C | 0.157 | 0.241 | 0.302 | 0.358 | 0.442 | 0.001 | 0.551 |
|  | mean | 0.173 | 0.232 | 0.288 | 0.354 | 0.434 | 0.001 | 0.555 |
|  | % viability | 31.152 | 41.84 | 51.86 | 63.81 | 78.21 |  | |
| **Petroleum ether fraction** | A | 0.222 | 0.269 | 0.343 | 0.435 | 0.462 | 0.001 | 0.606 |
|  | B | 0.213 | 0.274 | 0.361 | 0.441 | 0.471 | 0.003 | 0.587 |
|  | C | 0.218 | 0.266 | 0.337 | 0.425 | 0.466 | 0.001 | 0.601 |
|  | mean | 0.2177 | 0.26967 | 0.347 | 0.434 | 0.466 | 0.002 | 0.598 |
|  | % viability | 36.399 | 45.0948 | 58.027 | 72.52 | 77.98 |  | |

**Table S11:** % viability and IC_50_ of total methanolic extract and fractions of *Moringa oleifera* in MTT assay against HCT116 cancer cell line.

| **Dose-response curve (X axis: log conc. and Y axis: % viability)** | **log conc.** | **% viability** | **IC_50_** |
| --- | --- | --- | --- |
| **Staurosporine**  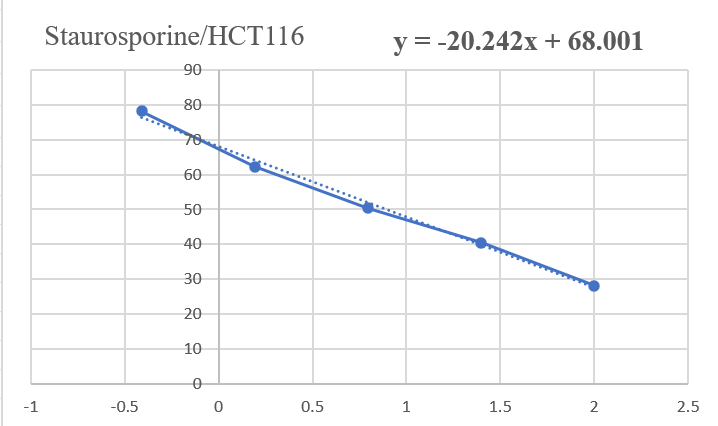 | **2** | 28.1513 | **7.75±0.38** |
|  | **1.39794** | 40.4562 |  |
|  | **0.79588** | 50.4202 |  |
|  | **0.19312** | 62.2449 |  |
|  | **-0.40894** | 78.2113 |  |
| **Total methanolic extract**  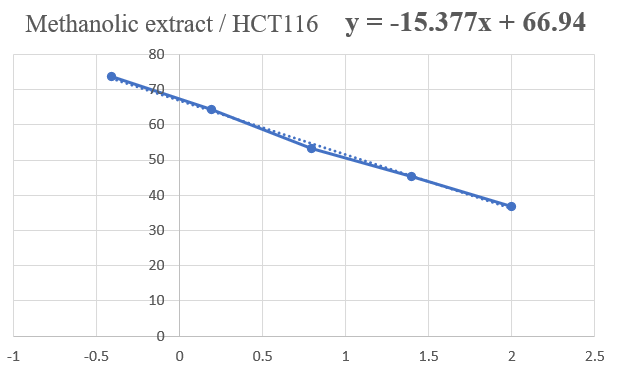 | **2** | 36.8478 | **12.64±0.74** |
|  | **1.39794** | 45.3261 |  |
|  | **0.79588** | 53.3152 |  |
|  | **0.19312** | 64.4565 |  |
|  | **-0.40894** | 73.587 |  |
| **Ethyl acetate fraction**  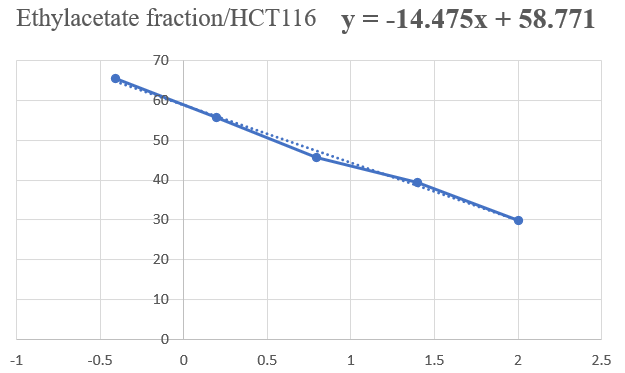 | **2** | 29.93 | **4.038**±0.13 |
|  | **1.39794** | 39.46 |  |
|  | **0.79588** | 45.76 |  |
|  | **0.19312** | 55.74 |  |
|  | **-0.40894** | 65.38 |  |
| Methylene chloride fraction  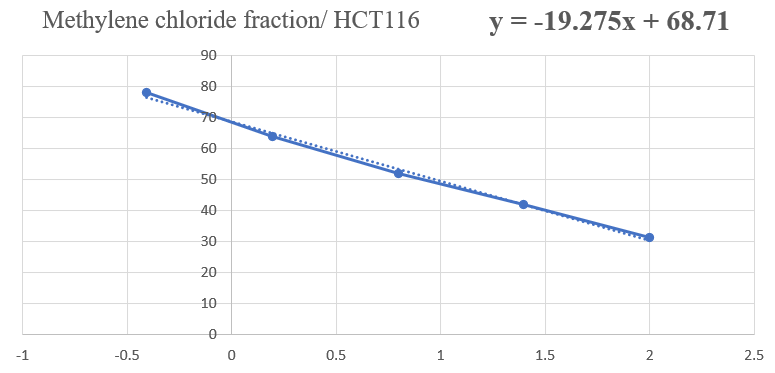 | **2** | 31.15 | **9.346±**0.55 |
|  | **1.39794** | 41.84 |  |
|  | **0.79588** | 51.86 |  |
|  | **0.19312** | 63.81 |  |
|  | **-0.40894** | 78.21 |  |
| **Petroleum ether fraction**  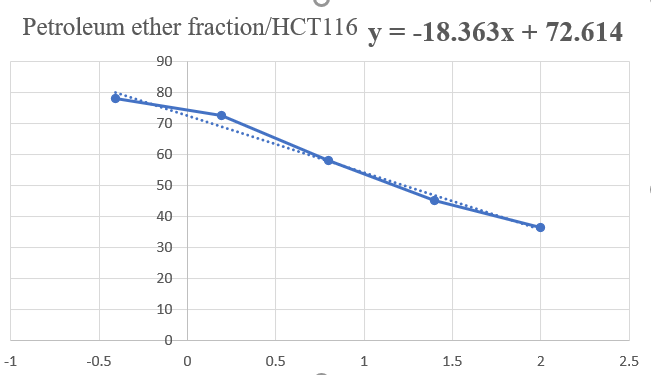 | **2** | 36.3991 | **17.04**±0.55 |
|  | **1.39794** | 45.0948 |  |
|  | **0.79588** | 58.0268 |  |
|  | **0.19312** | 72.5195 |  |
|  | **-0.40894** | 77.9822 |  |

**Table S12:** Absorbance of total methanolic extract and different fractions of *Moringa oleifera* in MTT assay against HepG2 cell lines.

| **HepG2** | | | | | | | | |
| --- | --- | --- | --- | --- | --- | --- | --- | --- |
| **Staurosporine** | Absorbance | 100ug | 25ug | 6.3ug | 1.6ug | 0.4ug | Blank | cc |
|  | A | 0.189 | 0.245 | 0.287 | 0.342 | 0.422 | 0.001 | 0.572 |
|  | B | 0.185 | 0.238 | 0.297 | 0.339 | 0.419 | 0.003 | 0.569 |
|  | C | 0.181 | 0.246 | 0.271 | 0.328 | 0.435 | 0.001 | 0.555 |
|  | mean | 0.185 | 0.243 | 0.285 | 0.33633 | 0.42533 | 0.002 | 0.565 |
|  | % viability | 32.7241 | 42.9835 | 50.4127 | 59.4929 | 75.2358 |  | |
| **Total methanolic extract** | A | 0.213 | 0.283 | 0.366 | 0.422 | 0.505 | 0.001 | 0.583 |
|  | B | 0.209 | 0.275 | 0.372 | 0.431 | 0.516 | 0.001 | 0.572 |
|  | C | 0.221 | 0.292 | 0.354 | 0.418 | 0.497 | 0.001 | 0.591 |
|  | mean | 0.21433 | 0.28333 | 0.364 | 0.42367 | 0.506 | 0.001 | 0.582 |
|  | % viability | 36.827 | 48.6827 | 62.543 | 72.795 | 86.9416 |  | |
| **Ethyl acetate fraction** | A | 0.142 | 0.191 | 0.251 | 0.321 | 0.367 | 0.001 | 0.575 |
|  | B | 0.133 | 0.187 | 0.233 | 0.303 | 0.382 | 0.001 | 0.564 |
|  | C | 0.157 | 0.193 | 0.237 | 0.297 | 0.364 | 0.001 | 0.588 |
|  | mean | 0.144 | 0.1903 | 0.2403 | 0.307 | 0.371 | 0.0004 | 0.576 |
|  | % viability | 25.0145 | 33.063 | 41.749 | 53.329 | 64.447 |  | |
| **Methylene chloride fraction** | A | 0.203 | 0.279 | 0.352 | 0.441 | 0.505 | 0.001 | 0.572 |
|  | B | 0.217 | 0.265 | 0.359 | 0.439 | 0.493 | 0.003 | 0.569 |
|  | C | 0.233 | 0.281 | 0.367 | 0.442 | 0.487 | 0.001 | 0.555 |
|  | mean | 0.2177 | 0.275 | 0.359 | 0.441 | 0.495 | 0.002 | 0.565 |
|  | % viability | 38.502 | 48.64 | 63.56 | 77.95 | 87.56 |  | |
| **Petroleum ether fraction** | A | 0.213 | 0.253 | 0.296 | 0.344 | 0.395 | 0.001 | 0.575 |
|  | B | 0.191 | 0.269 | 0.313 | 0.361 | 0.416 | 0.001 | 0.564 |
|  | C | 0.221 | 0.272 | 0.307 | 0.355 | 0.422 | 0.001 | 0.588 |
|  | mean | 0.2083 | 0.26467 | 0.3053 | 0.353 | 0.411 | 0.0004 | 0.576 |
|  | % viability | 36.19 | 45.9757 | 53.04 | 61.38 | 71.4 |  | |

**Table S13:** % viability and IC_50_ of total methanolic extract and fractions of *Moringa oleifera* in MTT assay against HepG2 cancer cell line.

| **Dose-response curve (X axis: log conc. and Y axis: % viability)** | **log conc.** | **% viability** | **IC_50_** |
| --- | --- | --- | --- |
| **Staurosporine**  **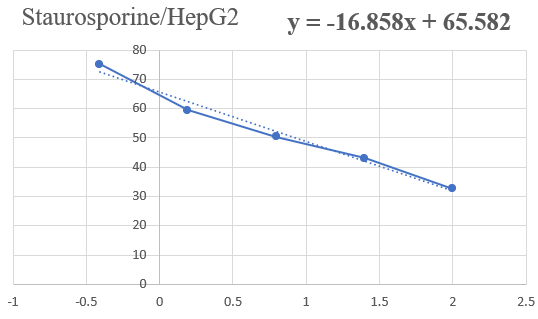** | **2** | 32.7241 | **8.401±0.41** |
|  | **1.39794** | 42.9835 |  |
|  | **0.79588** | 50.4127 |  |
|  | **0.19312** | 59.4929 |  |
|  | **-0.40894** | 75.2358 |  |
| **Total methanolic extract**  **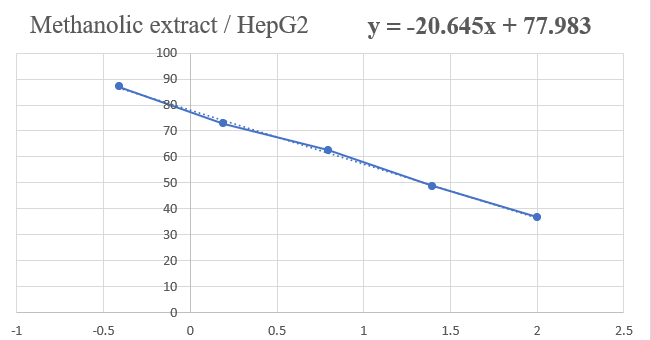** | **2** | 36.827 | **22.67±1.33** |
|  | **1.39794** | 48.6827 |  |
|  | **0.79588** | 62.543 |  |
|  | **0.19312** | 72.795 |  |
|  | **-0.40894** | 86.9416 |  |
| Ethyl acetate fraction  **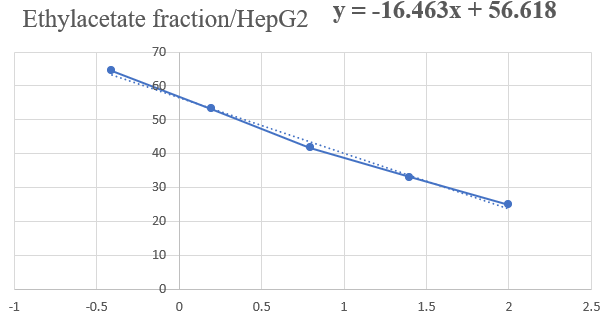** | **2** | 25.01 | **2.523±0.08** |
|  | **1.39794** | 33.06 |  |
|  | **0.79588** | 41.75 |  |
|  | **0.19312** | 53.33 |  |
|  | **-0.40894** | 64.45 |  |
| Methylene chloride fraction  **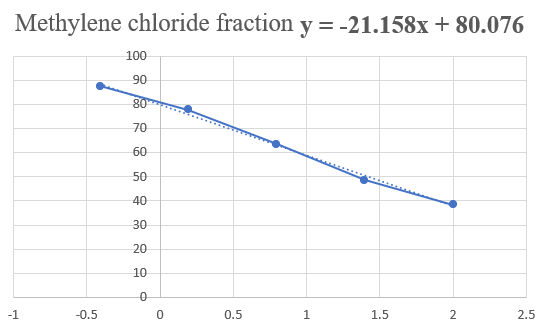** | **2** | 38.5 | **26.4±1.55** |
|  | **1.39794** | 48.64 |  |
|  | **0.79588** | 63.56 |  |
|  | **0.19312** | 77.95 |  |
|  | **-0.40894** | 87.56 |  |
| Petroleum ether fraction  **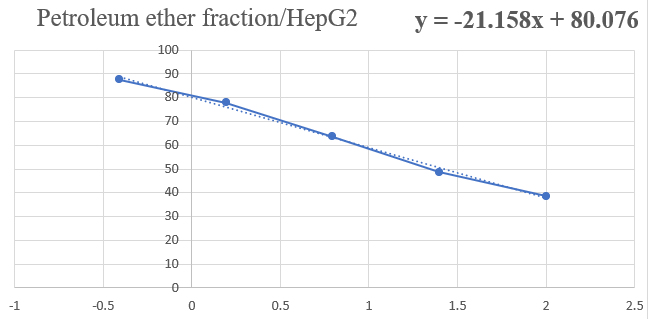** | **2** | 36.1899 | **11.17**±0.36 |
|  | **1.39794** | 45.9757 |  |
|  | **0.79588** | 53.04 |  |
|  | **0.19312** | 61.3781 |  |
|  | **-0.40894** | 71.3955 |  |

**Table S14:** Absorbance of isolated and semisynthetic compound of *Moringa oleifera* in MTT assay against HCT116 cancer cell lines

| **HCT116** | | | | | | | | |
| --- | --- | --- | --- | --- | --- | --- | --- | --- |
| **Staurosporine** | Absorbance | 100ug | 25ug | 6.3ug | 1.6ug | 0.4ug | Blank | cc |
|  | A | 0.161 | 0.231 | 0.276 | 0.351 | 0.434 | 0.001 | 0.572 |
|  | B | 0.147 | 0.228 | 0.283 | 0.337 | 0.428 | 0.003 | 0.569 |
|  | C | 0.161 | 0.215 | 0.281 | 0.349 | 0.441 | 0.001 | 0.555 |
|  | mean | 0.15633 | 0.22467 | 0.28 | 0.34567 | 0.43433 | 0.002 | 0.565 |
|  | % viability | 28.1513 | 40.4562 | 50.4202 | 62.2449 | 78.2113 |  |  |
| **M_1_** | A | 0.227 | 0.311 | 0.364 | 0.424 | 0.465 | 0.001 | 0.566 |
|  | B | 0.231 | 0.307 | 0.381 | 0.418 | 0.481 | 0.002 | 0.581 |
|  | C | 0.196 | 0.294 | 0.377 | 0.437 | 0.469 | 0.001 | 0.549 |
|  | mean | 0.218 | 0.304 | 0.374 | 0.426 | 0.472 | 0.0013 | 0.5653 |
|  | % viability | 38.561 | 53.77 | 66.156 | 75.41 | 83.43 |  |  |
| **M_2_** | A | 0.221 | 0.294 | 0.353 | 0.418 | 0.464 | 0.001 | 0.622 |
|  | B | 0.237 | 0.288 | 0.364 | 0.404 | 0.483 | 0.002 | 0.643 |
|  | C | 0.225 | 0.291 | 0.359 | 0.421 | 0.475 | 0.001 | 0.619 |
|  | mean | 0.22767 | 0.291 | 0.35867 | 0.41433 | 0.474 | 0.001 | 0.628 |
|  | % viability | 36.2527 | 46.3376 | 57.1125 | 65.9766 | 75.4777 |  |  |
| **M_3_** | A | 0.194 | 0.262 | 0.343 | 0.422 | 0.466 | 0.001 | 0.566 |
|  | B | 0.185 | 0.267 | 0.358 | 0.419 | 0.452 | 0.002 | 0.581 |
|  | C | 0.188 | 0.263 | 0.351 | 0.439 | 0.471 | 0.001 | 0.549 |
|  | mean | 0.189 | 0.264 | 0.3507 | 0.4267 | 0.463 | 0.0013 | 0.5653 |
|  | % viability | 33.432 | 46.698 | 62.028 | 75.472 | 81.899 |  |  |
| **S1_M2_** | A | 0.245 | 0.331 | 0.387 | 0.437 | 0.505 | 0.001 | 0.622 |
|  | B | 0.237 | 0.329 | 0.393 | 0.444 | 0.513 | 0.002 | 0.643 |
|  | C | 0.241 | 0.307 | 0.375 | 0.429 | 0.486 | 0.001 | 0.619 |
|  | mean | 0.241 | 0.322 | 0.385 | 0.437 | 0.501 | 0.001 | 0.628 |
|  | % viability | 38.376 | 51.33 | 61.31 | 69.53 | 79.83 |  |  |
| **S2_M2_** | A | 0.192 | 0.247 | 0.287 | 0.342 | 0.442 | 0.003 | 0.618 |
|  | B | 0.187 | 0.255 | 0.313 | 0.339 | 0.471 | 0.001 | 0.595 |
|  | C | 0.212 | 0.232 | 0.295 | 0.347 | 0.439 | 0.001 | 0.627 |
|  | mean | 0.197 | 0.245 | 0.298 | 0.343 | 0.451 | 0.002 | 0.613 |
|  | % viability | 32.12 | 39.89 | 48.64 | 55.87 | 73.48 |  |  |

**Table S15:** % viability and IC_50_ of isolated and semisynthetic compound of *Moringa oleifera* in MTT assay against HCT116 cancer cell line

| **Dose-response curve (X axis: log conc. and Y axis: % viability)** | **log conc.** | **% viability** | **IC_50_** |
| --- | --- | --- | --- |
| **Staurosporine**  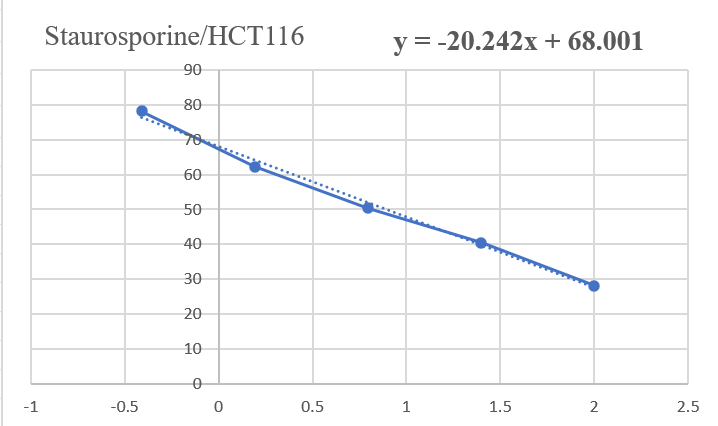 | **2** | 28.1513 | **7.75**±0.38 µg/mL  (16.62µM) |
|  | **1.39794** | 40.4562 |  |
|  | **0.79588** | 50.4202 |  |
|  | **0.19312** | 62.2449 |  |
|  | **-0.40894** | 78.2113 |  |
| **M_1_**  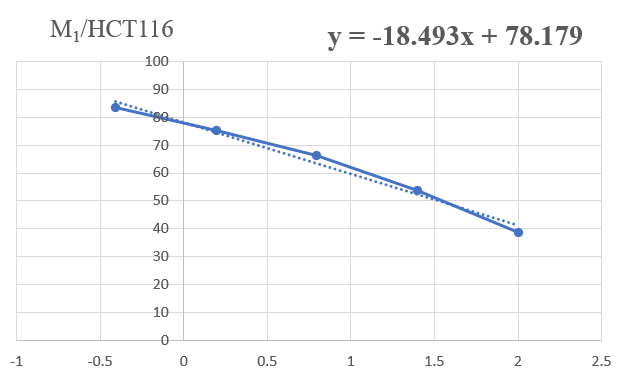 | **2** | 38.56 | **33.41±1.42** µg/mL  **(**124.54µM) |
|  | **1.39794** | 53.77 |  |
|  | **0.79588** | 66.16 |  |
|  | **0.19312** | 75.41 |  |
|  | **-0.40894** | 83.43 |  |
| **M_2_**  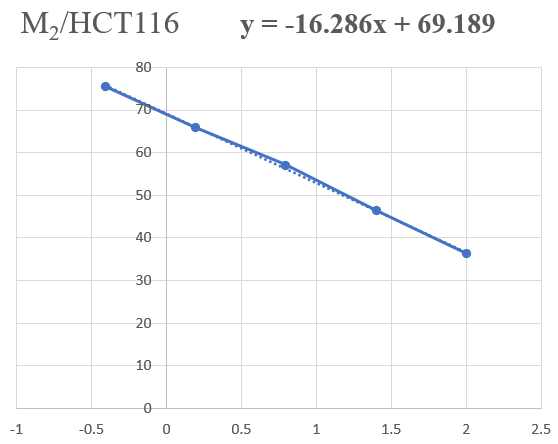 | **2** | 36.2527 | **15.07±**0.88 µg/mL  **(**55.75 µM) |
|  | **1.39794** | 46.3376 |  |
|  | **0.79588** | 57.1125 |  |
|  | **0.19312** | 65.9766 |  |
|  | **-0.40894** | 75.4777 |  |
| **M_3_**  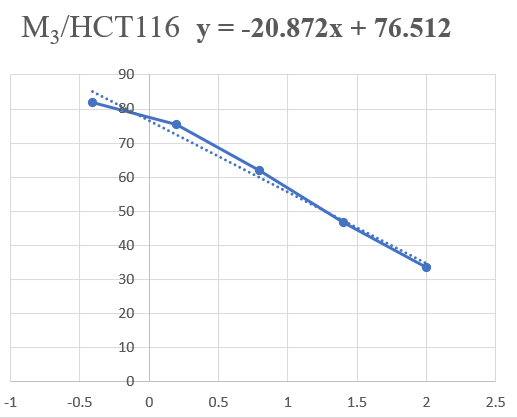 | **2** | 33.432 | **18.63**±0.79 µg/mL  **(**43.08µM) |
|  | **1.39794** | 46.698 |  |
|  | **0.79588** | 62.028 |  |
|  | **0.19312** | 75.472 |  |
|  | **-0.40894** | 81.899 |  |
| **S1_M2_**  **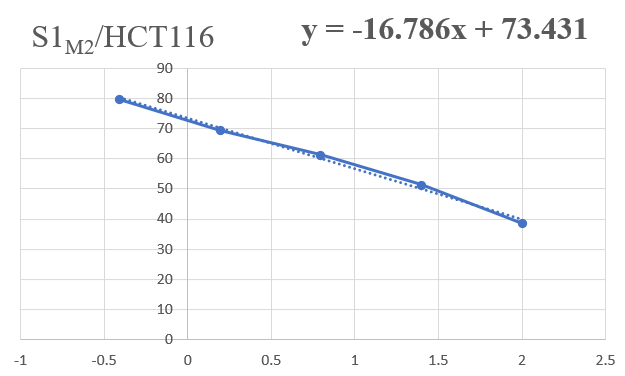** | **2** | 38.38 | **24.87±**1.46 µg/mL  31.45µM) |
|  | **1.39794** | 51.33 |  |
|  | **0.79588** | 61.31 |  |
|  | **0.19312** | 69.53 |  |
|  | **-0.40894** | 79.83 |  |
| **S2_M2_**  **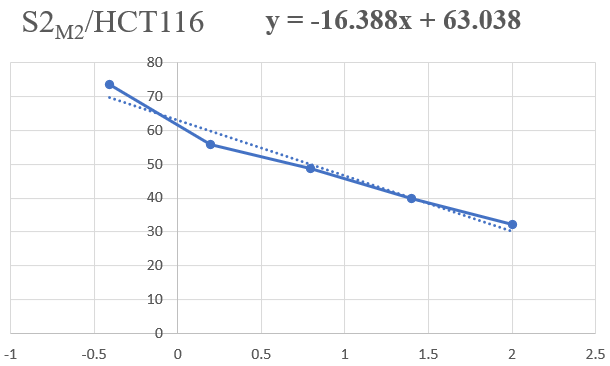** | **2** | 32.12 | **6.246±**0.37 µg/mL  **(**11.52 µM) |
|  | **1.39794** | 39.89 |  |
|  | **0.79588** | 48.64 |  |
|  | **0.19312** | 55.87 |  |
|  | **-0.40894** | 73.48 |  |

**Table S16:** Absorbance of isolated and semisynthetic compound of Moringa *oleifera* in MTT assay against HepG2 cancer cell lines

| **HepG2** | | | | | | | | |
| --- | --- | --- | --- | --- | --- | --- | --- | --- |
| **Staurosporine** | Absorbance | 100ug | 25ug | 6.3ug | 1.6ug | 0.4ug | Blank | cc |
|  | A | 0.189 | 0.245 | 0.287 | 0.342 | 0.422 | 0.001 | 0.572 |
|  | B | 0.185 | 0.238 | 0.297 | 0.339 | 0.419 | 0.003 | 0.569 |
|  | C | 0.181 | 0.246 | 0.271 | 0.328 | 0.435 | 0.001 | 0.555 |
|  | mean | 0.185 | 0.243 | 0.285 | 0.33633 | 0.42533 | 0.002 | 0.565 |
|  | % viability | 32.7241 | 42.9835 | 50.4127 | 59.4929 | 75.2358 |  | |
| M_1_ | A | 0.215 | 0.243 | 0.328 | 0.376 | 0.446 | 0.001 | 0.565 |
|  | B | 0.222 | 0.257 | 0.341 | 0.383 | 0.427 | 0.003 | 0.581 |
|  | C | 0.193 | 0.229 | 0.311 | 0.391 | 0.451 | 0.001 | 0.559 |
|  | mean | 0.21 | 0.243 | 0.3267 | 0.383 | 0.441 | 0.0017 | 0.5683 |
|  | % viability | 36.95 | 42.76 | 57.478 | 67.45 | 77.65 |  | |
| M_2_ | A | 0.161 | 0.221 | 0.299 | 0.359 | 0.424 | 0.001 | 0.549 |
|  | B | 0.149 | 0.218 | 0.273 | 0.372 | 0.413 | 0.001 | 0.561 |
|  | C | 0.155 | 0.228 | 0.303 | 0.361 | 0.395 | 0.001 | 0.554 |
|  | mean | 0.155 | 0.22233 | 0.29167 | 0.364 | 0.41067 | 0.0004 | 0.555 |
|  | % viability | 27.9447 | 40.0841 | 52.5841 | 65.625 | 74.0385 |  | |
| M_3_ | A | 0.138 | 0.218 | 0.265 | 0.342 | 0.397 | 0.001 | 0.565 |
|  | B | 0.144 | 0.221 | 0.272 | 0.339 | 0.412 | 0.003 | 0.581 |
|  | C | 0.146 | 0.193 | 0.284 | 0.327 | 0.443 | 0.001 | 0.559 |
|  | mean | 0.1427 | 0.2107 | 0.2737 | 0.336 | 0.4173 | 0.0017 | 0.5683 |
|  | % viability | 25.103 | 37.067 | 48.152 | 59.12 | 73.431 |  | |
| S1_M2_ | A | 0.208 | 0.265 | 0.322 | 0.364 | 0.428 | 0.001 | 0.549 |
|  | B | 0.216 | 0.261 | 0.304 | 0.357 | 0.419 | 0.001 | 0.561 |
|  | C | 0.194 | 0.249 | 0.333 | 0.366 | 0.441 | 0.001 | 0.554 |
|  | mean | 0.206 | 0.258 | 0.32 | 0.362 | 0.429 | 0.0004 | 0.555 |
|  | % viability | 37.139 | 46.57 | 57.63 | 65.32 | 77.4 |  | |
| S2_M2_ | A | 0.144 | 0.211 | 0.265 | 0.331 | 0.376 | 0.001 | 0.583 |
|  | B | 0.165 | 0.208 | 0.257 | 0.304 | 0.384 | 0.001 | 0.572 |
|  | C | 0.147 | 0.216 | 0.272 | 0.323 | 0.368 | 0.001 | 0.591 |
|  | mean | 0.152 | 0.212 | 0.265 | 0.319 | 0.376 | 0.001 | 0.582 |
|  | % viability | 26.117 | 36.37 | 45.48 | 54.87 | 64.6 |  | |

**Table S17:** % viability and IC_50_ of isolated and semisynthetic compound of *Moringa oleifera* in MTT assay against HepG2 cancer cell lines

| **Dose-response curve (X axis: log conc. and Y axis: % viability)** | **log conc.** | **% viability** | **IC_50_** |
| --- | --- | --- | --- |
| **Staurosporine**  **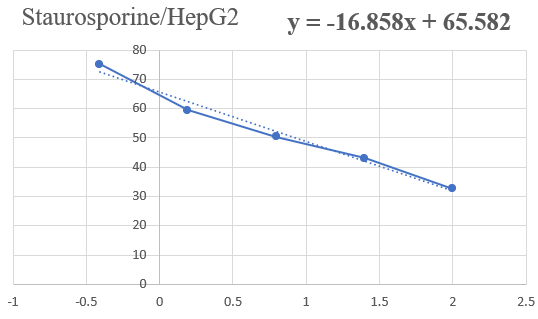** | **2** | 32.7241 | **8.401±**0.41 µg/mL  **(**18.00µM) |
|  | **1.39794** | 42.9835 |  |
|  | **0.79588** | 50.4127 |  |
|  | **0.19312** | 59.4929 |  |
|  | **-0.40894** | 75.2358 |  |
| **M_1_**  **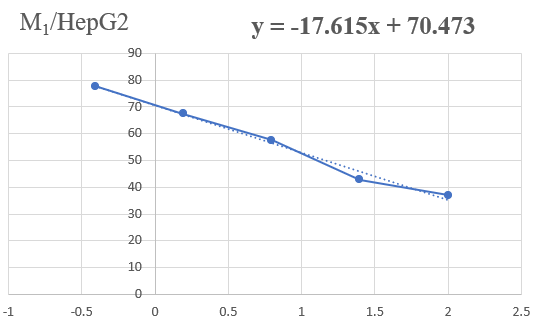** | **2** | 36.95 | **14.52±0.53** µg/mL  (54.13µM) |
|  | **1.39794** | 42.76 |  |
|  | **0.79588** | 57.48 |  |
|  | **0.19312** | 67.45 |  |
|  | **-0.40894** | 77.65 |  |
| **M_2_**  **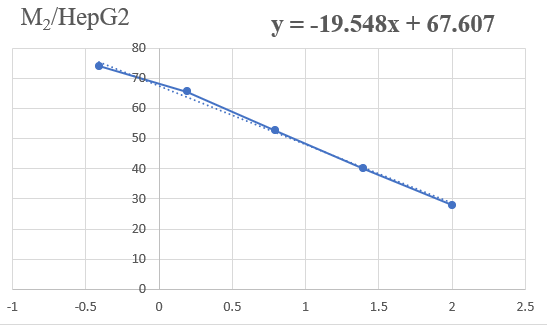** | **2** | 27.9447 | **7.956±**0.47 µg/mL  **(**29.45 µM) |
|  | **1.39794** | 40.0841 |  |
|  | **0.79588** | 52.5841 |  |
|  | **0.19312** | 65.625 |  |
|  | **-0.40894** | 74.0385 |  |
| **M_3_**  **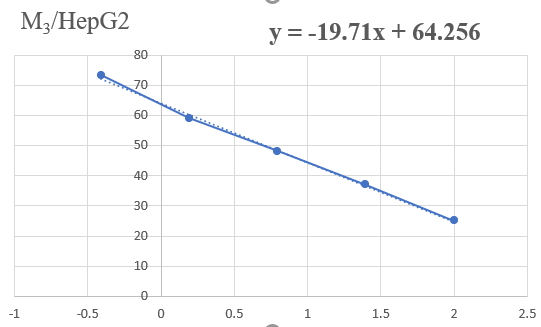** | **2** | 25.103 | **5.28±0.19** µg/mL  **(**12.21 µM) |
|  | **1.39794** | 37.067 |  |
|  | **0.79588** | 48.152 |  |
|  | **0.19312** | 59.12 |  |
|  | **-0.40894** | 73.431 |  |
| **S1_M2_**  **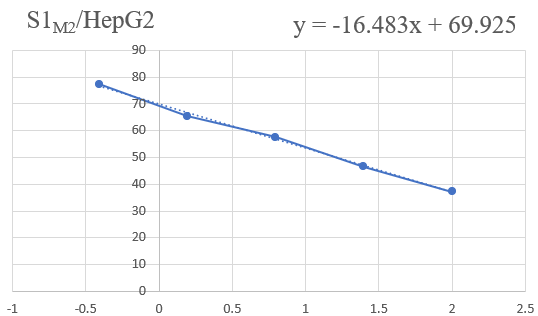** | **2** | 37.14 | **16.18±**0.95 µg/mL  **(**20.46 µM) |
|  | **1.39794** | 46.57 |  |
|  | **0.79588** | 57.63 |  |
|  | **0.19312** | 65.32 |  |
|  | **-0.40894** | 77.4 |  |
| **S2_M2_**  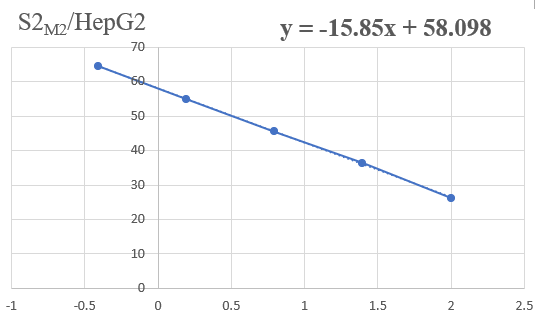 | **2** | 26.12 | **3.243±**0.19 µg/mL  (5.97µM) |
|  | **1.39794** | 36.37 |  |
|  | **0.79588** | 45.48 |  |
|  | **0.19312** | 54.87 |  |
|  | **-0.40894** | 64.6 |  |

| **WI38** | | | | | | | | |
| --- | --- | --- | --- | --- | --- | --- | --- | --- |
| **Staurosporine** | Absorbance | 100ug | 25ug | 6.3ug | 1.6ug | 0.4ug | Blank | cc |
|  | A | 0.164 | 0.237 | 0.309 | 0.361 | 0.415 | 0.001 | 0.484 |
|  | B | 0.159 | 0.242 | 0.292 | 0.349 | 0.393 | 0.001 | 0.475 |
|  | C | 0.173 | 0.251 | 0.321 | 0.353 | 0.387 | 0.001 | 0.462 |
|  | mean | 0.16533 | 0.24333 | 0.30733 | 0.35433 | 0.39833 | 0.001 | 0.474 |
|  | % viability | 34.905 | 51.3723 | 64.8839 | 74.8065 | 84.0957 |  | |
| M_2_ | A | 0.179 | 0.233 | 0.289 | 0.331 | 0.396 | 0.001 | 0.449 |
|  | B | 0.183 | 0.225 | 0.281 | 0.329 | 0.375 | 0.001 | 0.439 |
|  | C | 0.172 | 0.241 | 0.276 | 0.331 | 0.404 | 0.001 | 0.447 |
|  | mean | 0.178 | 0.233 | 0.282 | 0.33033 | 0.39167 | 0.0004 | 0.445 |
|  | % viability | 40 | 52.3596 | 63.3708 | 74.2322 | 88.015 |  | |
| S1_M2_ | A | 0.186 | 0.251 | 0.308 | 0.359 | 0.404 | 0.001 | 0.449 |
|  | B | 0.204 | 0.263 | 0.327 | 0.371 | 0.418 | 0.001 | 0.439 |
|  | C | 0.193 | 0.259 | 0.311 | 0.373 | 0.429 | 0.001 | 0.447 |
|  | mean | 0.1943 | 0.258 | 0.315 | 0.368 | 0.417 | 0.0004 | 0.445 |
|  | % viability | 43.67 | 57.9 | 70.86 | 82.62 | 93.71 |  | |
| S2_M2_ | A | 0.196 | 0.246 | 0.296 | 0.377 | 0.441 | 0.001 | 0.484 |
|  | B | 0.215 | 0.259 | 0.318 | 0.362 | 0.439 | 0.001 | 0.475 |
|  | C | 0.181 | 0.261 | 0.307 | 0.359 | 0.428 | 0.001 | 0.462 |
|  | mean | 0.1973 | 0.255 | 0.307 | 0.366 | 0.436 | 0.001 | 0.474 |
|  | % viability | 41.661 | 53.91 | 64.81 | 77.27 | 92.05 |  | |

**Table S18:** Absorbance of isolated and semisynthetic compound of *Moringa oleifera* in MTT assay against **WI38** cancer cell lines

**Table S19:** % viability and IC_50_ of isolated and semisynthetic compound of *Moringa oleifera* in MTT assay against **WI38** cancer cell lines

| **Dose-response curve (X axis: log conc. and Y axis: % viability)** | **log conc.** | **% viability** | **IC_50_** |
| --- | --- | --- | --- |
| **Staurosporine**  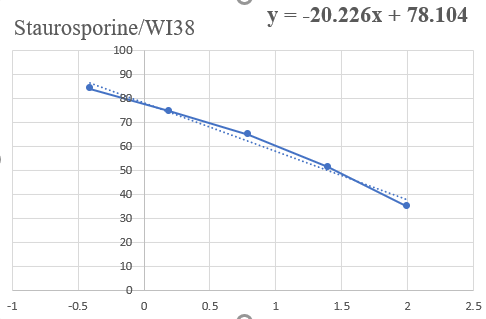 | **2** | 34.905 | **24.52±**1.27 µg/mL  **(**52.56µM) |
|  | **1.39794** | 51.3723 |  |
|  | **0.79588** | 64.8839 |  |
|  | **0.19312** | 74.8065 |  |
|  | **-0.40894** | 84.0957 |  |
| M2  **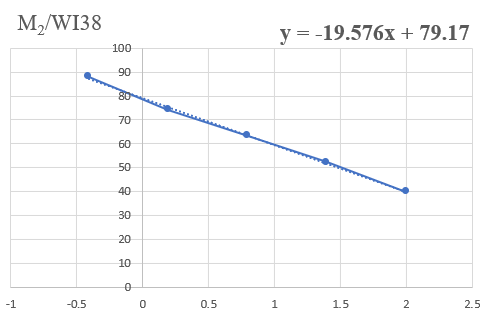** | **2** | 40 | **30.91±**1.88 µg/mL  **(**114.36 µM) |
|  | **1.39794** | 52.3596 |  |
|  | **0.79588** | 63.3708 |  |
|  | **0.19312** | 74.2322 |  |
|  | **-0.40894** | 88.015 |  |
| **S1_M2_**  **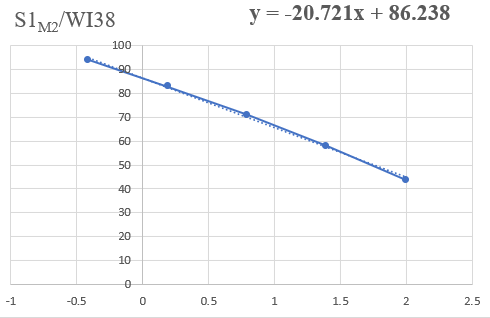** | **2** | 43.67 | **56.09±**3.04 µg/mL  **(**70.92 µM) |
|  | **1.39794** | 57.9 |  |
|  | **0.79588** | 70.86 |  |
|  | **0.19312** | 82.62 |  |
|  | **-0.40894** | 93.71 |  |
| **S2_M2_**  **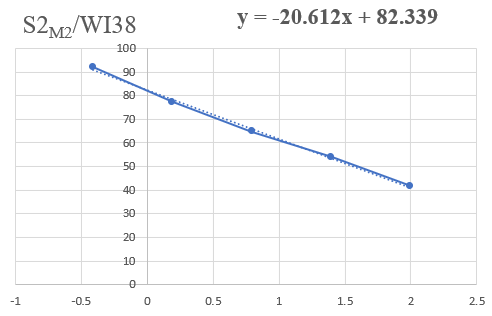** | **2** | 41.66 | **37.06±**2.31 µg/mL  **(**68.30 µM) |
|  | **1.39794** | 53.91 |  |
|  | **0.79588** | 64.81 |  |
|  | **0.19312** | 77.27 |  |
|  | **-0.40894** | 92.05 |  |

**Table S20:** IC50 and selectivity index of isolated compound M_2_ and semisynthetic compounds

| Compound | HepG2  IC_50_  µg/mL | HepG2  IC_50_  µM | HCT116  IC_50_  µg/mL | HCT116  IC_50_  µM | WI38  IC_50_  µg/mL | WI38  IC_50_  µM | Selectivity index  HepG2 | Selectivity index  HCT116 |
| --- | --- | --- | --- | --- | --- | --- | --- | --- |
| M_2_ | **7.956±**0.47 | 29.45 | **15.07±**0.88 | 55.75 | **30.91±**1.88 | 114.36 | 3.89 | 2.05 |
| S1_M2_ | **16.18±**0.95 | 20.46 | **24.87±**1.46 | 31.45 | **56.09±**3.04 | 70.92 | 3.47 | 2.25 |
| S2_M2_ | **3.243±**0.19 | 5.97 | **6.246±**0.37 | 11.52 | **37.06±**2.31 | 68.30 | 11.43 | 5.93 |
| **Staurosporine** | **8.401±**0.41 | 18.00 | **7.75**±0.38 | 16.62 | **24.52±**1.27 | 52.56 | 2.92 | 3.16 |

**Table S21**. % inhibition of isolated and semi-synthesized compounds against EGFR TK enzyme inhibition. All data are presented as mean value ± SD for three independent experiments

| EGFR Tk | | | | | | |
| --- | --- | --- | --- | --- | --- | --- |
| M_1_  _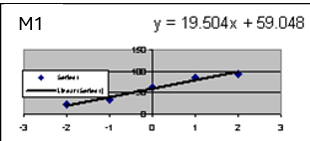_ | conc | log | %inhibition | ∆T | ∆RFU | K.Activity |
|  | 100 | 2 | 94.2 | 30 | 5.75 | 6.9 |
|  | 10 | 1 | 83.6 | 30 | 16.35 | 19.62 |
|  | 1 | 0 | 62 | 30 | 38.02 | 45.624 |
|  | 0.1 | -1 | 33.6 | 30 | 66.39 | 79.668 |
|  | 0.01 | -2 | 21.7 | 30 | 78.25 | 93.9 |
|  |  |  | 0 | 30 | 100 | 120 |
| M2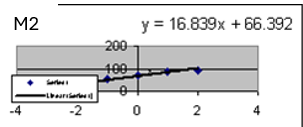 | 100 | 2 | 94.1 | 30 | 5.91 | 7.092 |
|  | 10 | 1 | 86.6 | 30 | 13.37 | 16.044 |
|  | 1 | 0 | 72.3 | 30 | 27.66 | 33.192 |
|  | 0.1 | -1 | 51.4 | 30 | 48.62 | 58.344 |
|  | 0.01 | -2 | 27.5 | 30 | 72.48 | 86.976 |
|  |  |  | 0 | 30 | 100 | 120 |
| M_3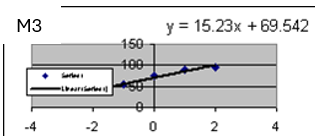_ | 100 | 2 | 95.2 | 30 | 4.77 | 5.724 |
|  | 10 | 1 | 88.4 | 30 | 11.61 | 13.932 |
|  | 1 | 0 | 74.1 | 30 | 25.88 | 31.056 |
|  | 0.1 | -1 | 53.4 | 30 | 46.61 | 55.932 |
|  | 0.01 | -2 | 36.6 | 30 | 63.42 | 76.104 |
|  |  |  | 0 | 30 | 100 | 120 |
| S_1M2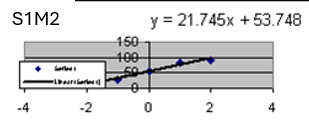_ | 100 | 2 | 92.7 | 30 | 7.26 | 8.712 |
|  | 10 | 1 | 81 | 30 | 19.02 | 22.824 |
|  | 1 | 0 | 56.7 | 30 | 43.26 | 51.912 |
|  | 0.1 | -1 | 27.5 | 30 | 72.45 | 86.94 |
|  | 0.01 | -2 | 10.7 | 30 | 89.27 | 107.12 |
|  |  |  | 0 | 30 | 100 | 120 |
| S_2M2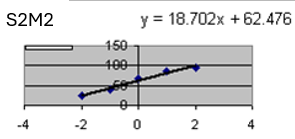_ | 100 | 2 | 93.7 | 30 | 9654 | 7.5392 |
|  | 10 | 1 | 87.1 | 30 | 19876 | 15.522 |
|  | 1 | 0 | 67.6 | 30 | 49772 | 38.869 |
|  | 0.1 | -1 | 40.5 | 30 | 91431 | 71.403 |
|  | 0.01 | -2 | 23.5 | 30 | 117564 | 91.811 |
| Erlotinib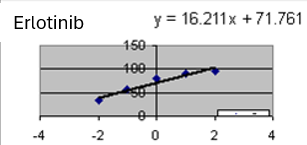 | 100 | 2 | 96.9 | 30 | 4832 | 3.7735 |
|  | 10 | 1 | 91.4 | 30 | 13229 | 10.331 |
|  | 1 | 0 | 80.8 | 30 | 29461 | 23.007 |
|  | 0.1 | -1 | 56.5 | 30 | 66884 | 52.233 |
|  | 0.01 | -2 | 33.3 | 30 | 102553 | 80.088 |
|  |  |  | 0 | 30 | 153667 | 120 |

**Table S22**. % inhibition of isolated and semi-synthesized compounds against CAIX enzyme inhibition. All data are presented as mean value ± SD for three independent experiments

| CAIX | | | | | | |
| --- | --- | --- | --- | --- | --- | --- |
| 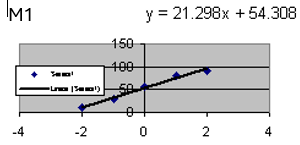M_1_ | conc | log | %inh | ∆T | ∆RFU | K.Activity |
|  | 100 | 2 | 92.3 | 30 | 7.72 | 9.264 |
|  | 10 | 1 | 81.1 | 30 | 18.91 | 22.692 |
|  | 1 | 0 | 57.3 | 30 | 42.65 | 51.18 |
|  | 0.1 | -1 | 29 | 30 | 71.03 | 85.236 |
|  | 0.01 | -2 | 11.8 | 30 | 88.15 | 105.78 |
|  |  |  | 0 | 30 | 100 | 120 |
| M_2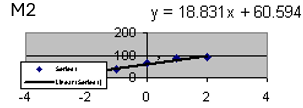_ | 100 | 2 | 94 | 30 | 6.03 | 7.236 |
|  | 10 | 1 | 84.2 | 30 | 15.75 | 18.9 |
|  | 1 | 0 | 62.7 | 30 | 37.26 | 44.712 |
|  | 0.1 | -1 | 40.1 | 30 | 59.86 | 71.832 |
|  | 0.01 | -2 | 21.9 | 30 | 78.13 | 93.756 |
|  |  |  | 0 | 30 | 100 | 120 |
| M_3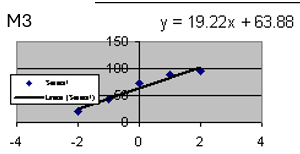_ | 100 | 2 | 94.8 | 30 | 5.15 | 6.18 |
|  | 10 | 1 | 87.7 | 30 | 12.31 | 14.772 |
|  | 1 | 0 | 73.1 | 30 | 26.94 | 32.328 |
|  | 0.1 | -1 | 42.4 | 30 | 57.59 | 69.108 |
|  | 0.01 | -2 | 21.4 | 30 | 78.61 | 94.332 |
|  |  |  | 0 | 30 | 100 | 120 |
| S_1M2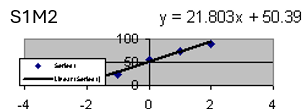_ | 100 | 2 | 90.6 | 30 | 9.44 | 11.328 |
|  | 10 | 1 | 74.9 | 30 | 25.11 | 30.132 |
|  | 1 | 0 | 56.1 | 30 | 43.85 | 52.62 |
|  | 0.1 | -1 | 22.7 | 30 | 77.28 | 92.736 |
|  | 0.01 | -2 | 7.63 | 30 | 92.37 | 110.84 |
|  |  |  | 0 | 30 | 100 | 120 |
| S_2M2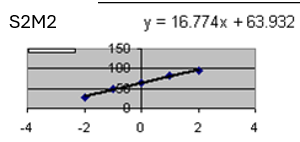_ | 100 | 2 | 94.6 | 30 | 0.126 | 6.4615 |
|  | 10 | 1 | 84.1 | 30 | 0.373 | 19.128 |
|  | 1 | 0 | 63.2 | 30 | 0.862 | 44.205 |
|  | 0.1 | -1 | 50.1 | 30 | 1.168 | 59.897 |
|  | 0.01 | -2 | 27.7 | 30 | 1.691 | 86.718 |
|  |  |  | 0 | 30 | 2.344 | 120 |
| Acetazolamide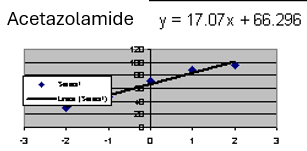 | 100 | 2 | 96.2 | 30 | 0.088 | 4.5128 |
|  | 10 | 1 | 83.2 | 30 | 0.392 | 20.103 |
|  | 1 | 0 | 62.2 | 30 | 0.885 | 45.385 |
|  | 0.1 | -1 | 48.9 | 30 | 1.196 | 61.333 |
|  | 0.01 | -2 | 40.7 | 30 | 1.388 | 71.179 |
|  |  |  | 0 | 30 | 2.344 | 120 |

| **Compound** | **IC_50_** | | | | | |
| --- | --- | --- | --- | --- | --- | --- |
|  | **EGFR TK** | |  | | **CAIX** | |
|  | **µg/mL** | **(µM)** | **µg/mL** | | | **(µM)** |
| **M_1_** | 0.344**±**0.014 | **1.28±0.014** | 0.628**±**0.026 | | | **2.34±0.03** |
| **M_2_** | 0.106**±**0.004 | **0.39±0.004** | 0.274**±**0.013 | | | **1.01±0.01** |
| **M_3_** | 0.052**±**0.002 | **0.12±0.002** | 0.19**±**0.007 | | | **0.44±0.01** |
| **S1_M2_** | 0.672**±**0.026 | **0.85±0.026** | 0.96**±**0.042 | | | **1.21±0.04** |
| **S2_M2_** | 0.215**±**0.008 | **0.40±0.008** | 0.148**±**0.005 | | | **0.27±0.01** |
| **Erlotinib** | 0.045**±0.002** | **0.11 ±0.01** | ………….. |  | | ………… |
| **Acetazolamide** | ………….. | ………… | 0.076**±**0.003 |  | | **0.49 ±0.01** |

**Table S23: IC_50_ values of isolated and semi-synthesized compounds for EGFR TK and CAIX enzymes inhibition with µg/mL and µM**


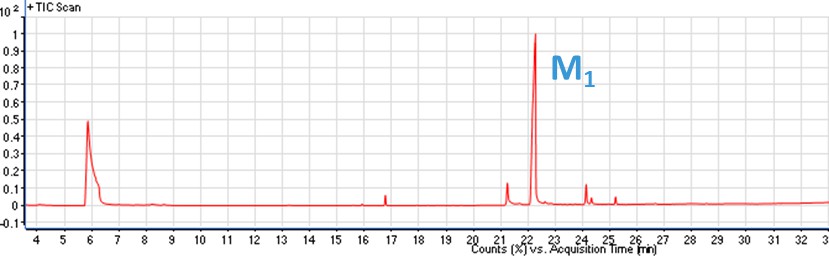


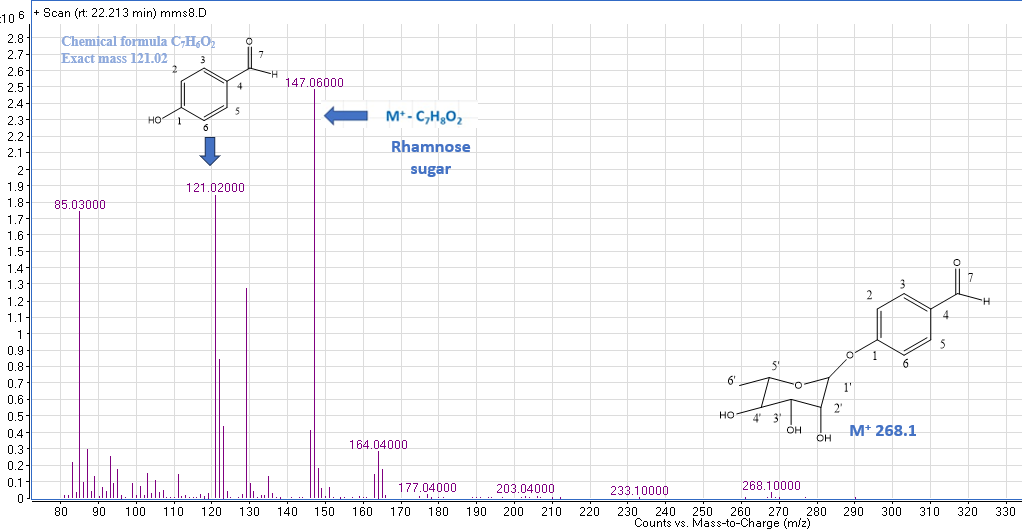


**Fig. S1.GC-MS spectrum and fragmentation of compound M_1_**


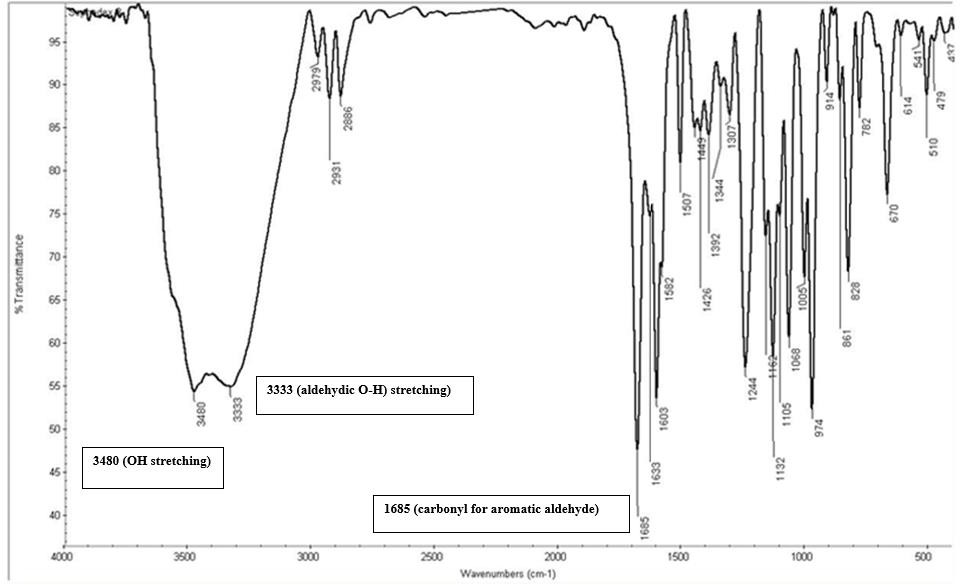


**Fig. S2 IR spectrum of compound M_1_**


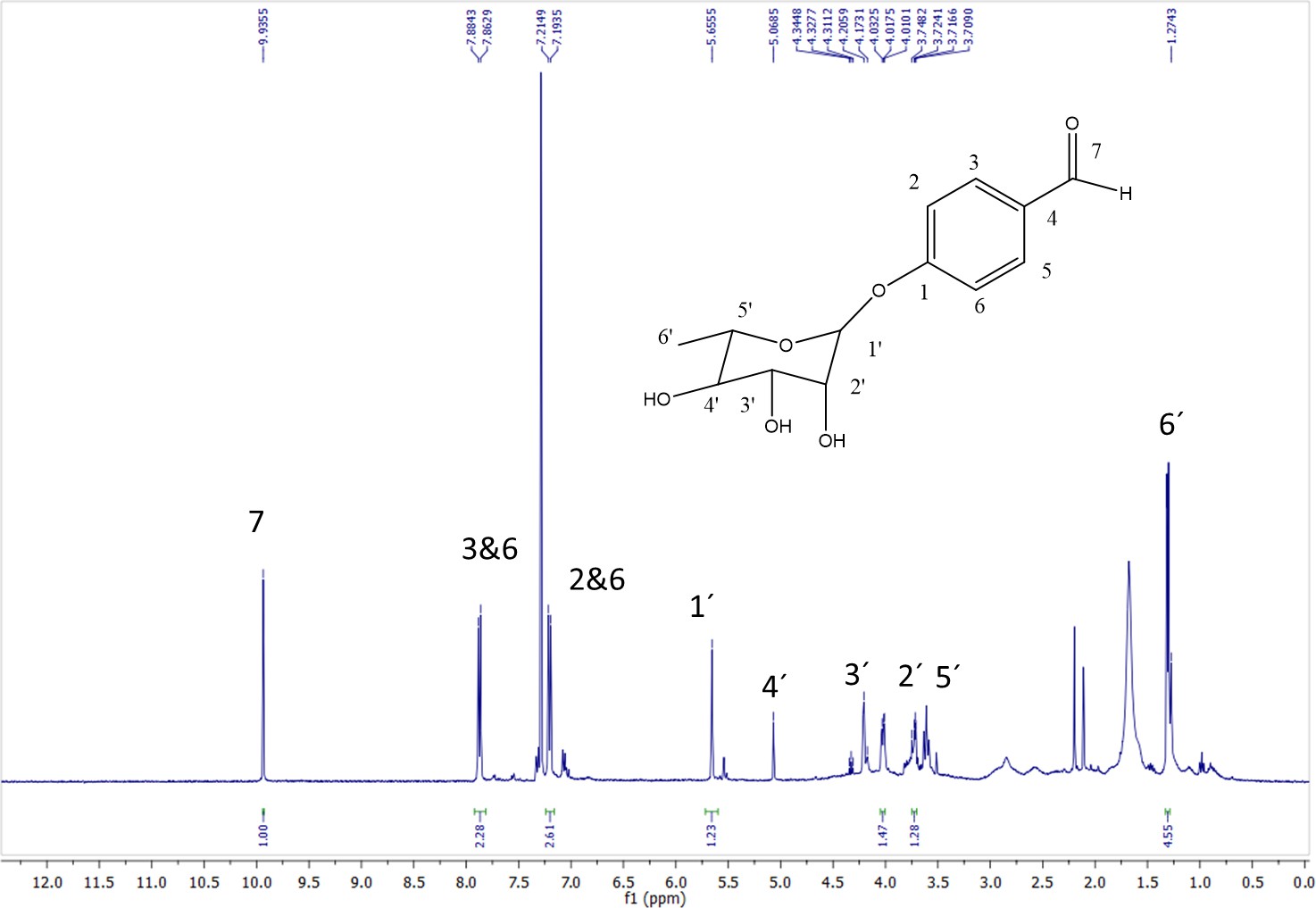


**Fig.S3: ^1^H spectrum (CDCl_3_, 400 MHz) of compound M_1_**


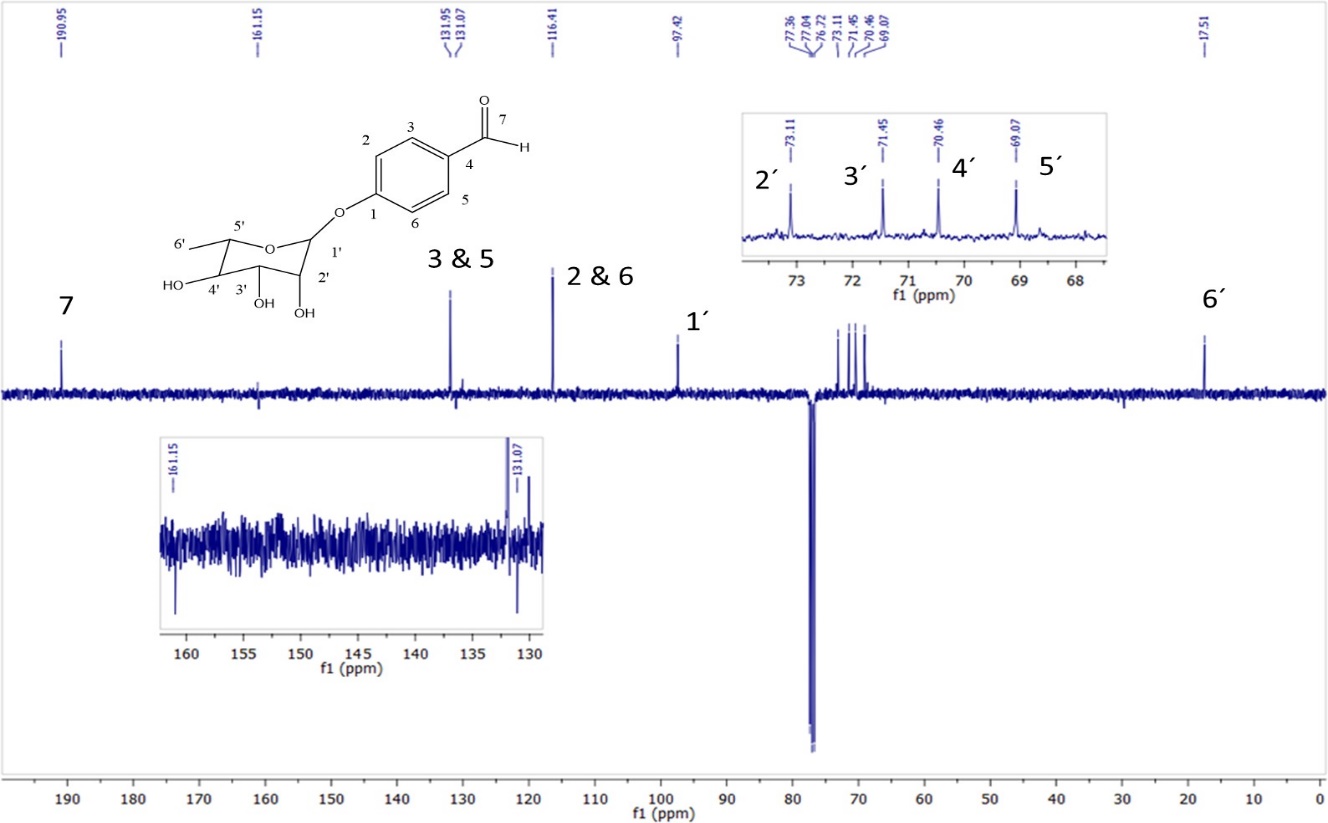


**Fig S4: APT of compound M_1_, (CDCl_3_ (**


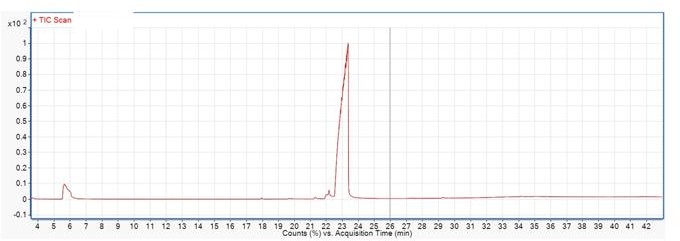


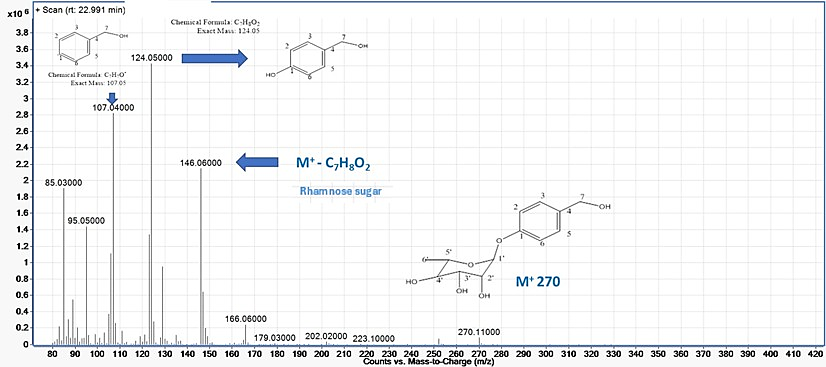

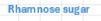


**Figure S5: GC-MS spectrum and fragmentation of compound M_2_**


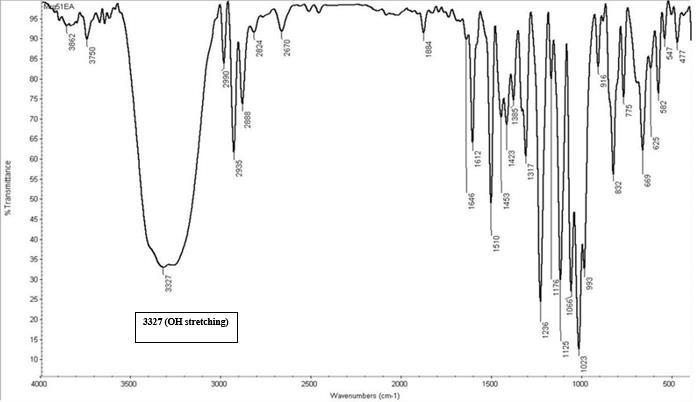


**Fig S6: IR spectrum of compound M_2_**


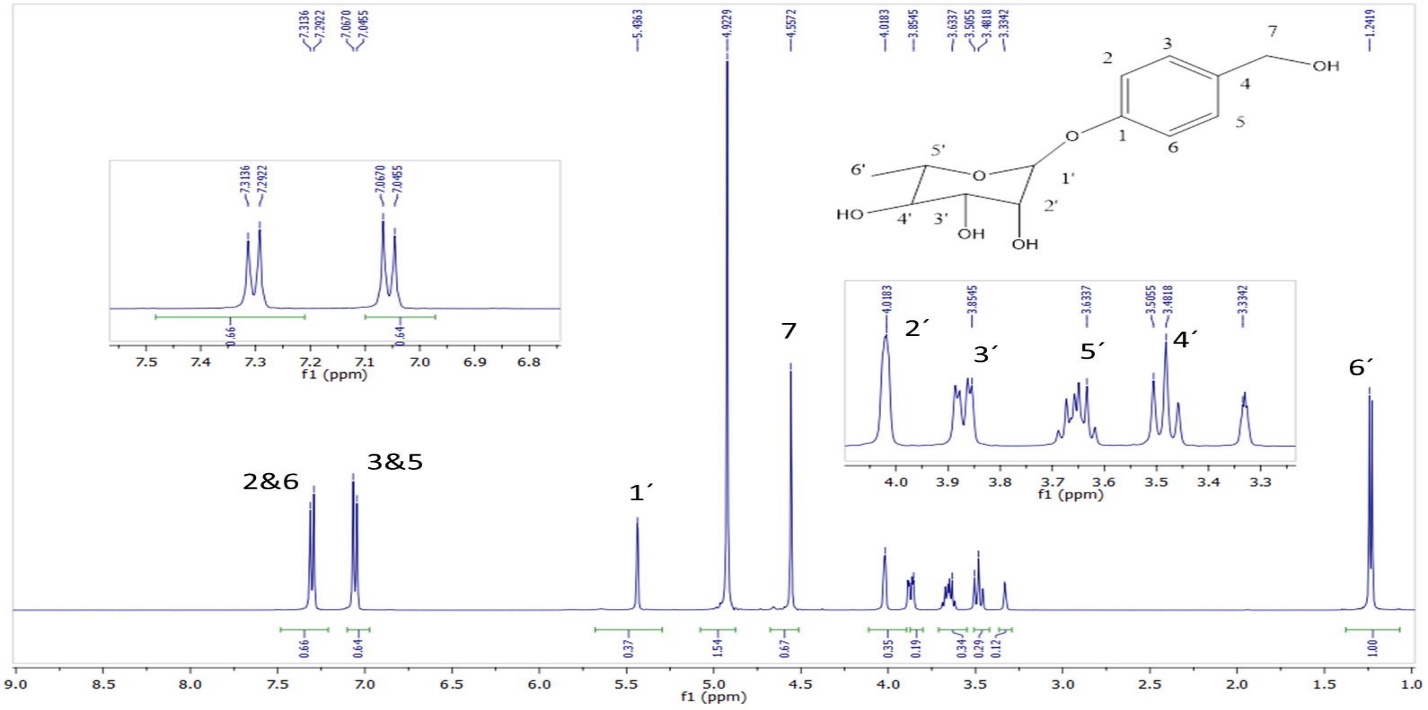


**Fig. S7: ^1^H-NMR spectrum of compound M_2_**


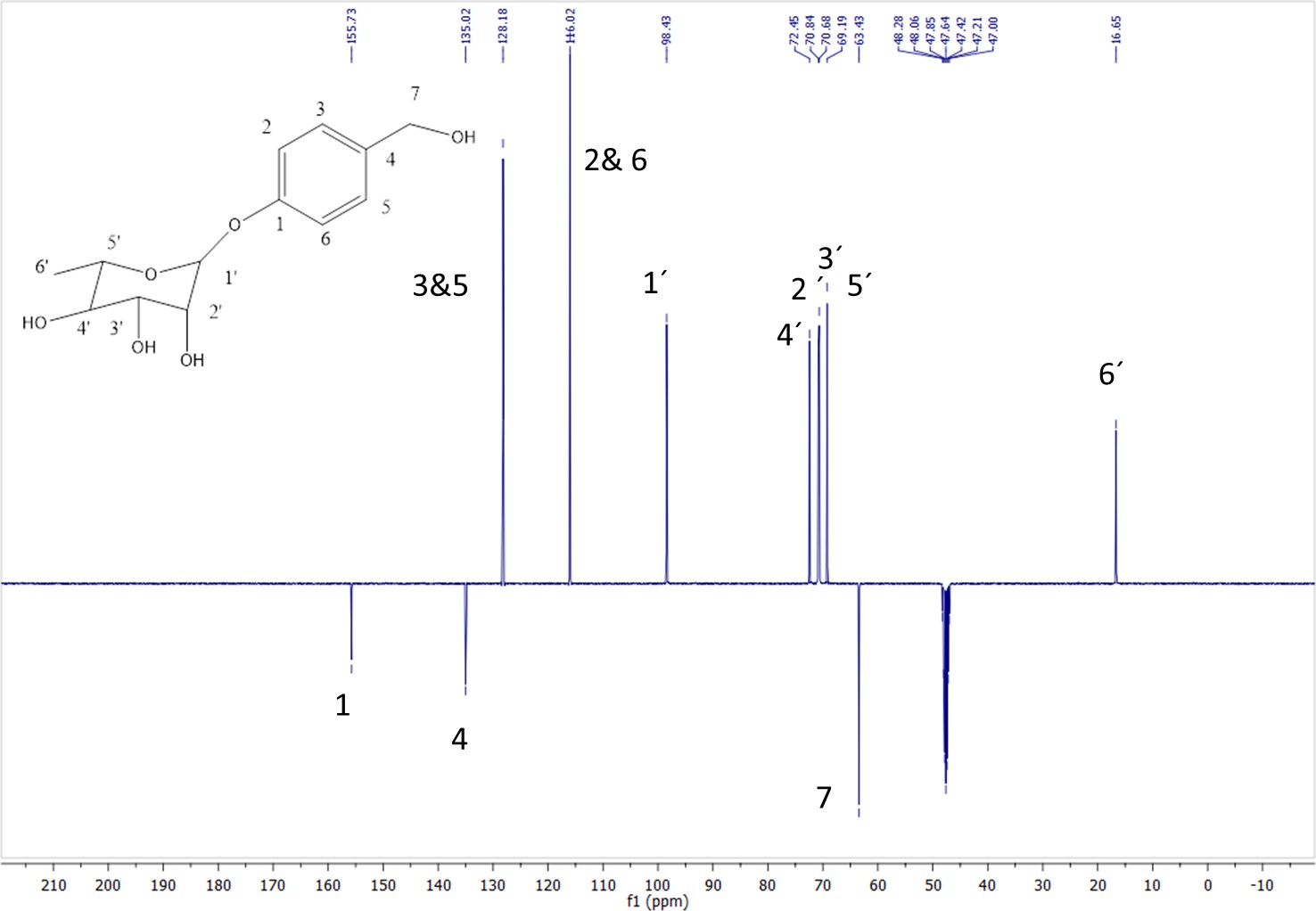


**Fig. S8: APT-NMR spectrum of compound M_2_**


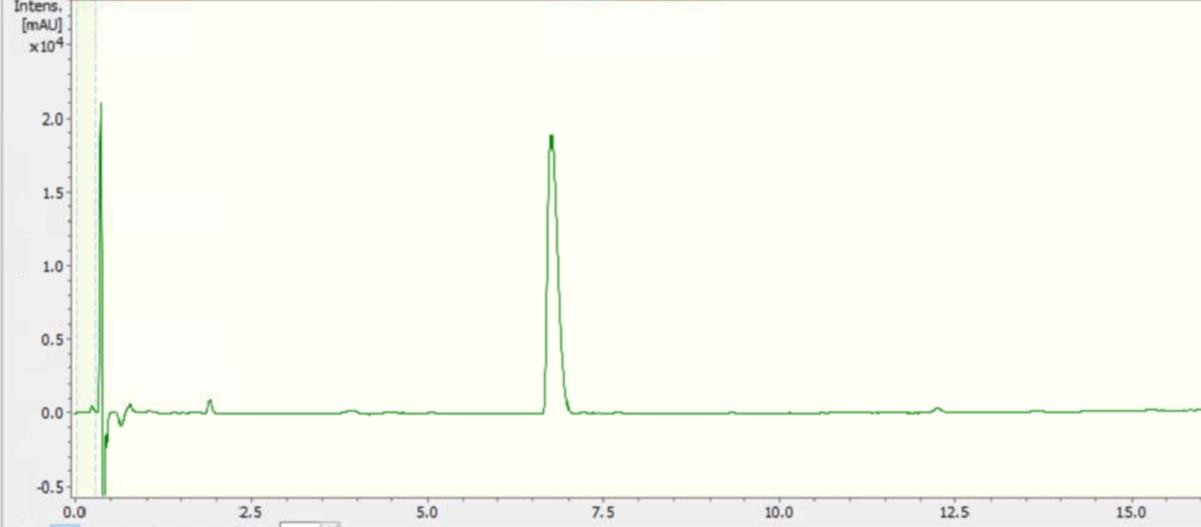


**(A)**

**
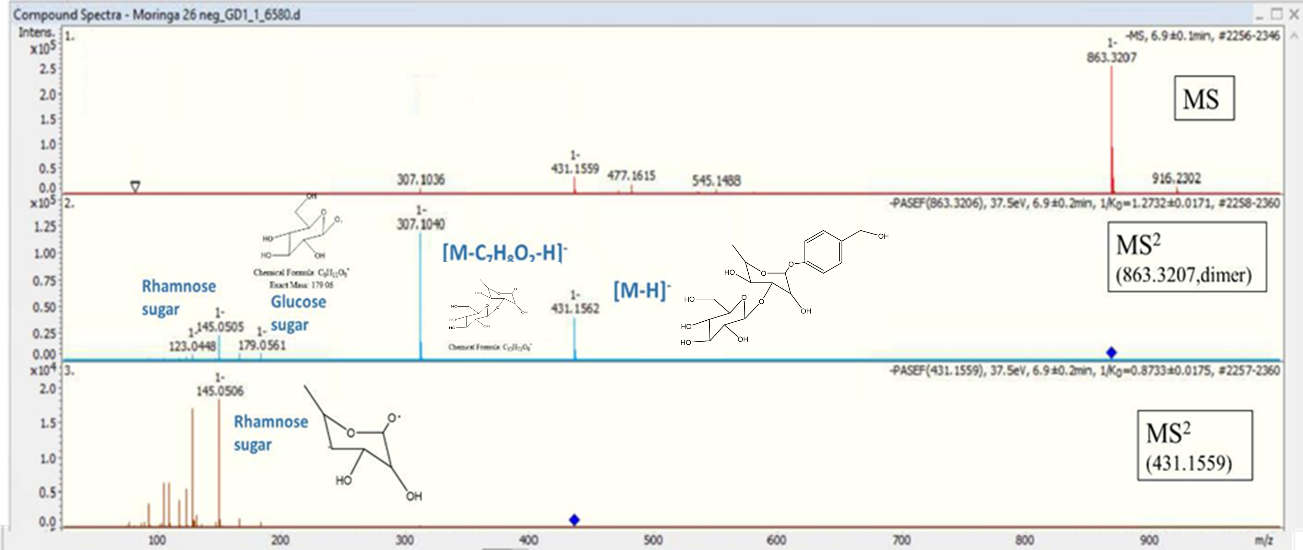
**

**(B)**

**Fig. S9:** HR-ESI-MS chromatogram of compound **M_3_** in negative ion mode. A) TIC chromatogram of compound M3 at Rt 6-9. B) MS^2^ fragment of compound **M_3_**


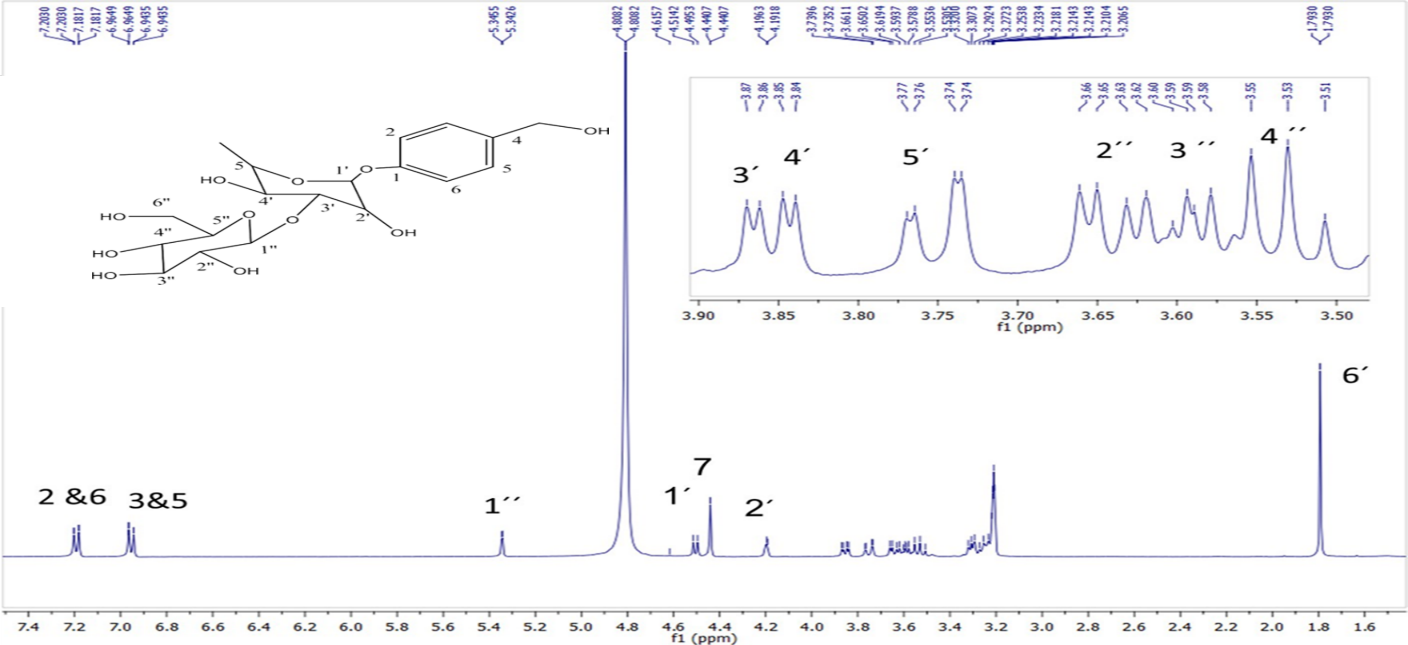

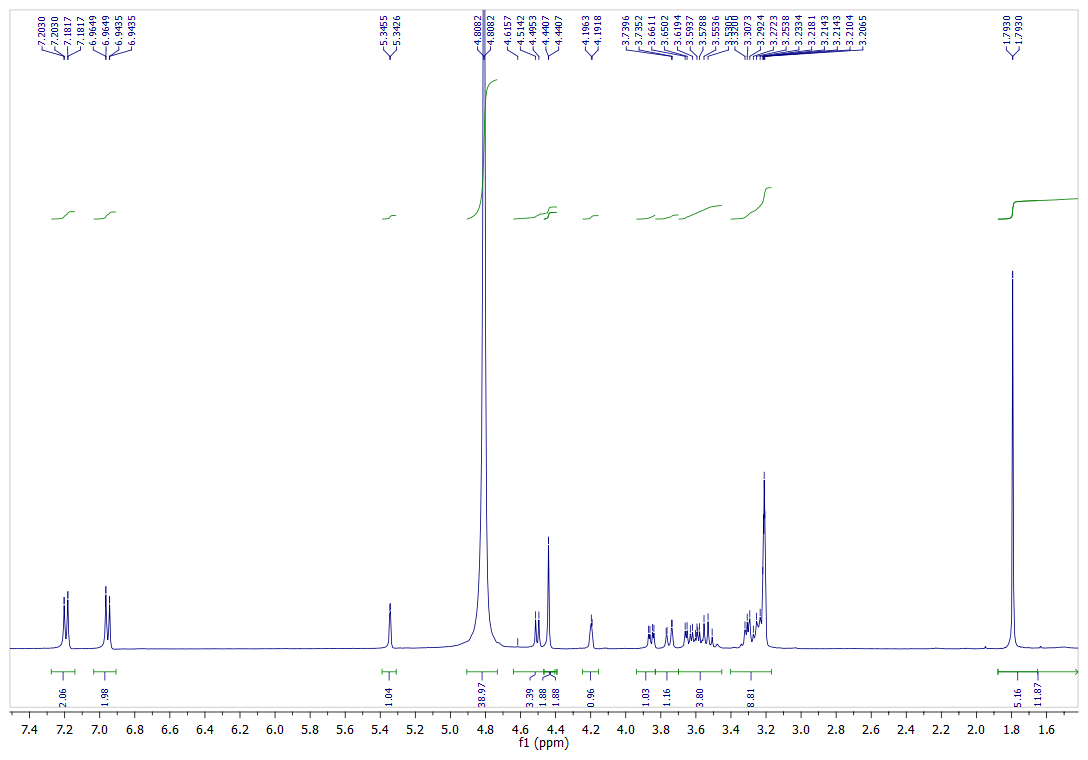


**Figure (40):1H-NMR spectrum of M3**

**Fig S10: ^1^H-NMR spectrum of compound M_3_**


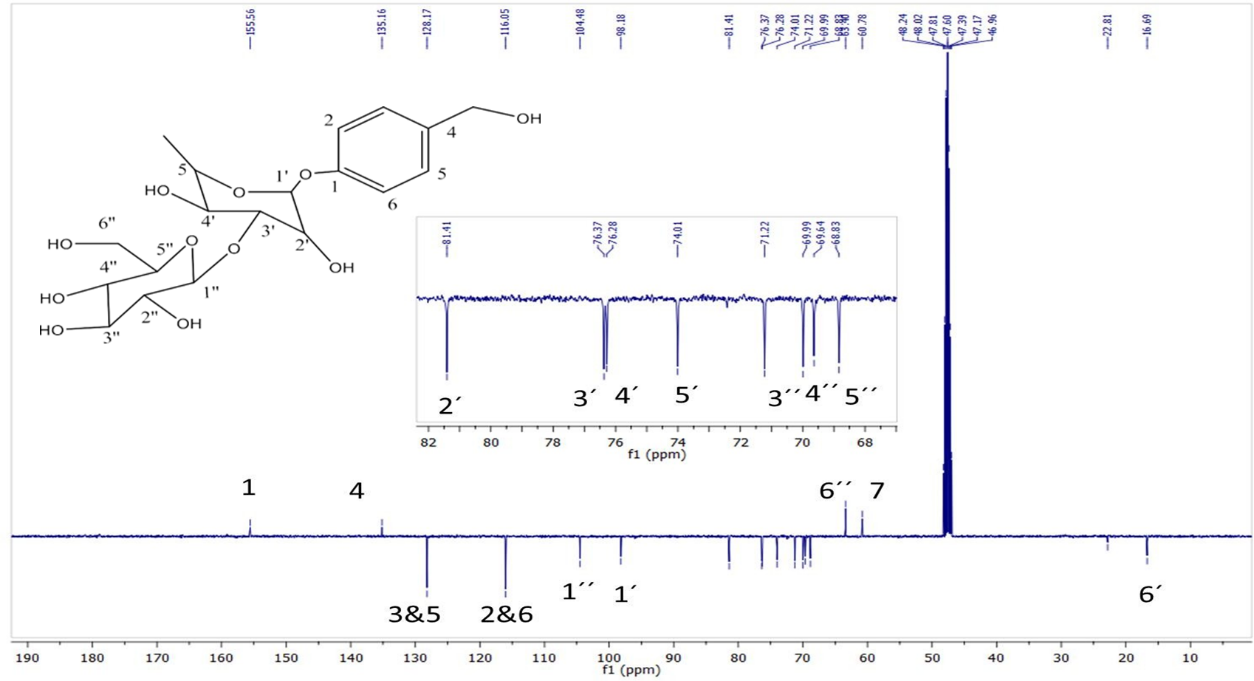


**Fig S11: APT-NMR spectrum of compound M_3_**

HMBC of compound **S1_M2_**


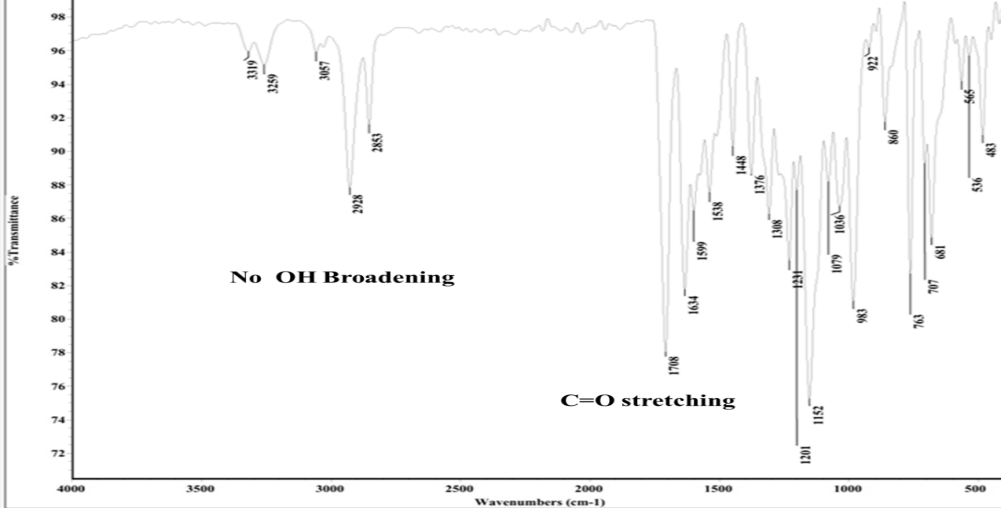


**Figure S12: IR spectrum of compound S1_M2_**


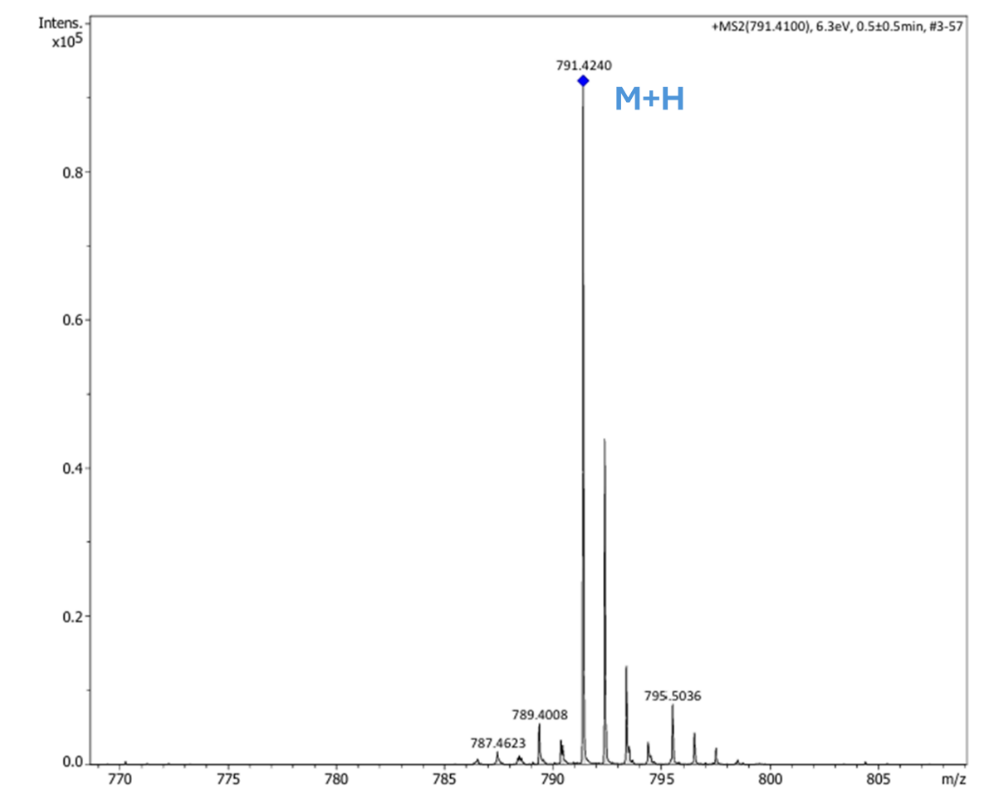


**Fig. S13: HR-ESI mass of compound S1_M2_**
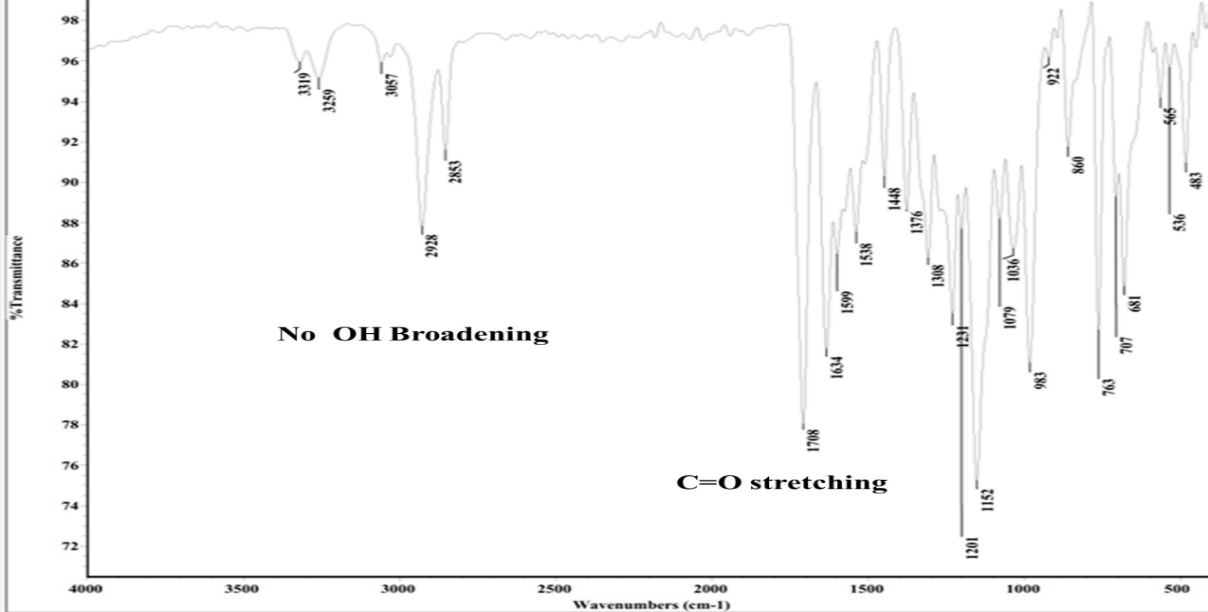


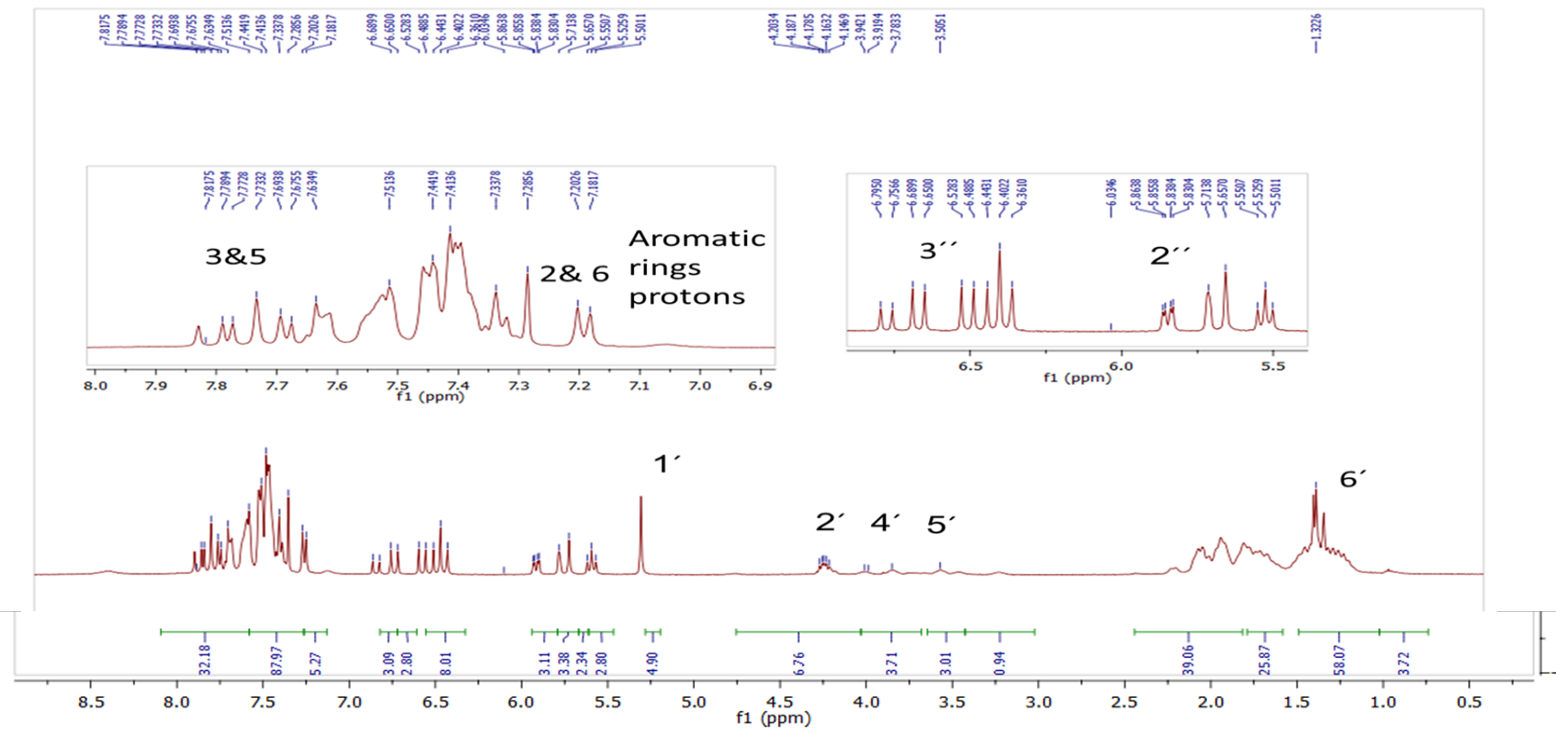


**Fig. S14 :^1^H-NMR spectrum of compound S1_M2_**

**
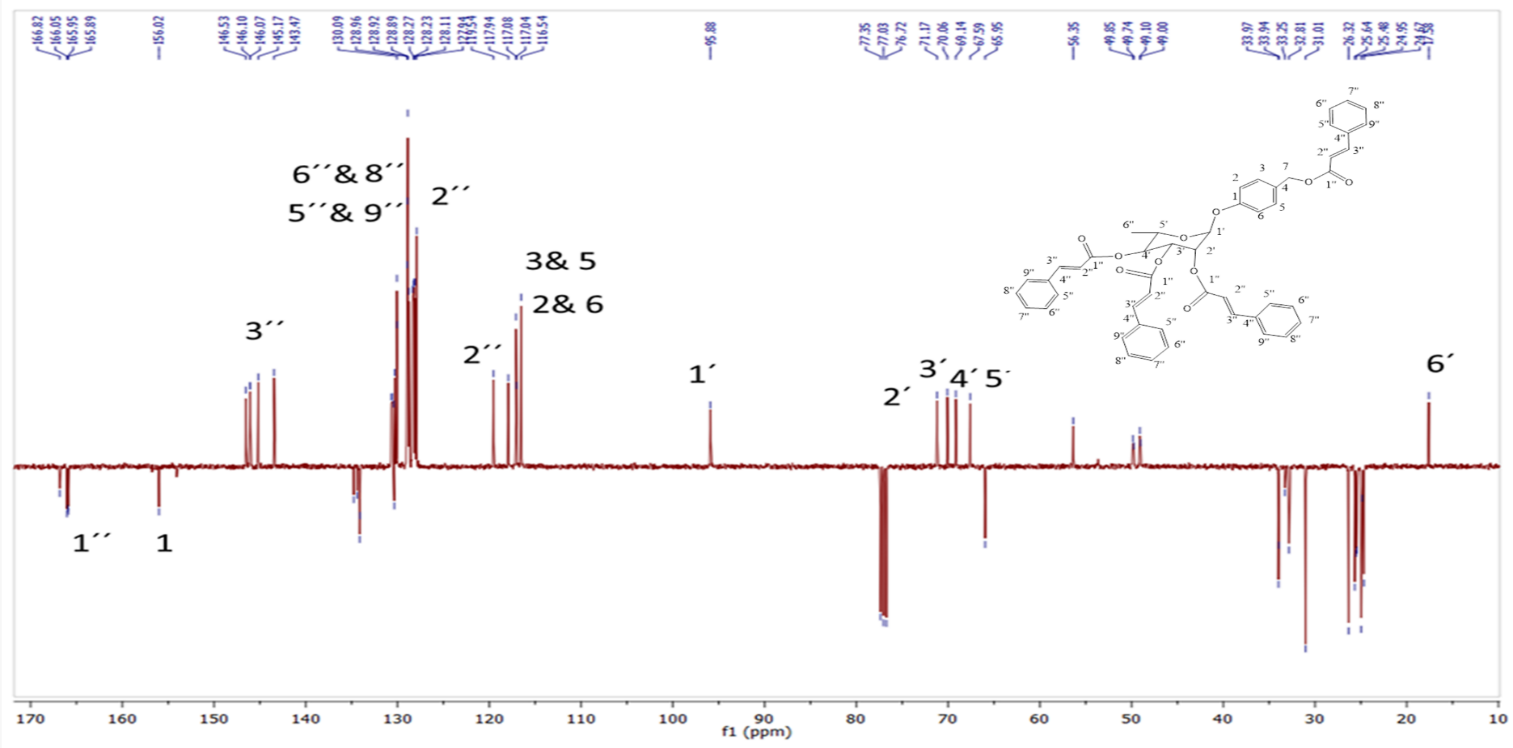
**

**Figure S15: APT-NMR spectrum of compound S1_M2_**

**
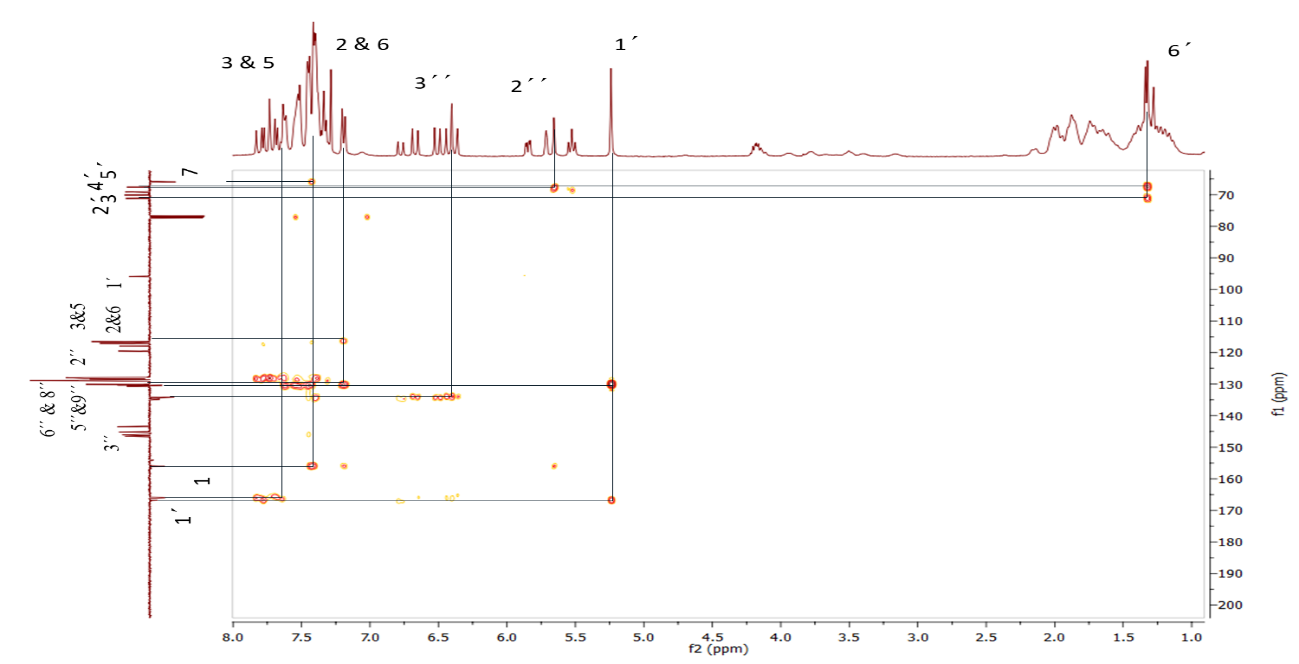
**

**Fig S16: HMBC-NMR spectrum of compound S1_M2_**

HMBC of compound S2_M2_


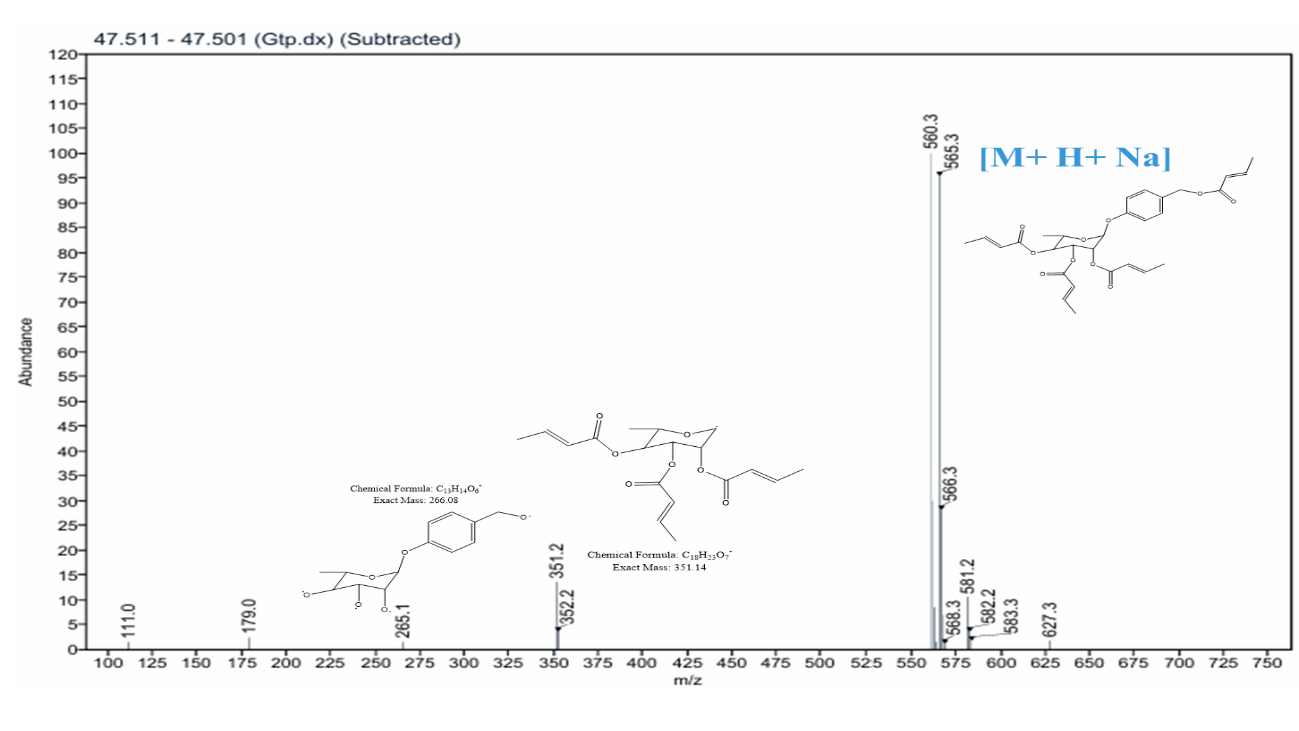


[M+Na]^+^

**Fig. S17: LC MS of compound S2_M2_**


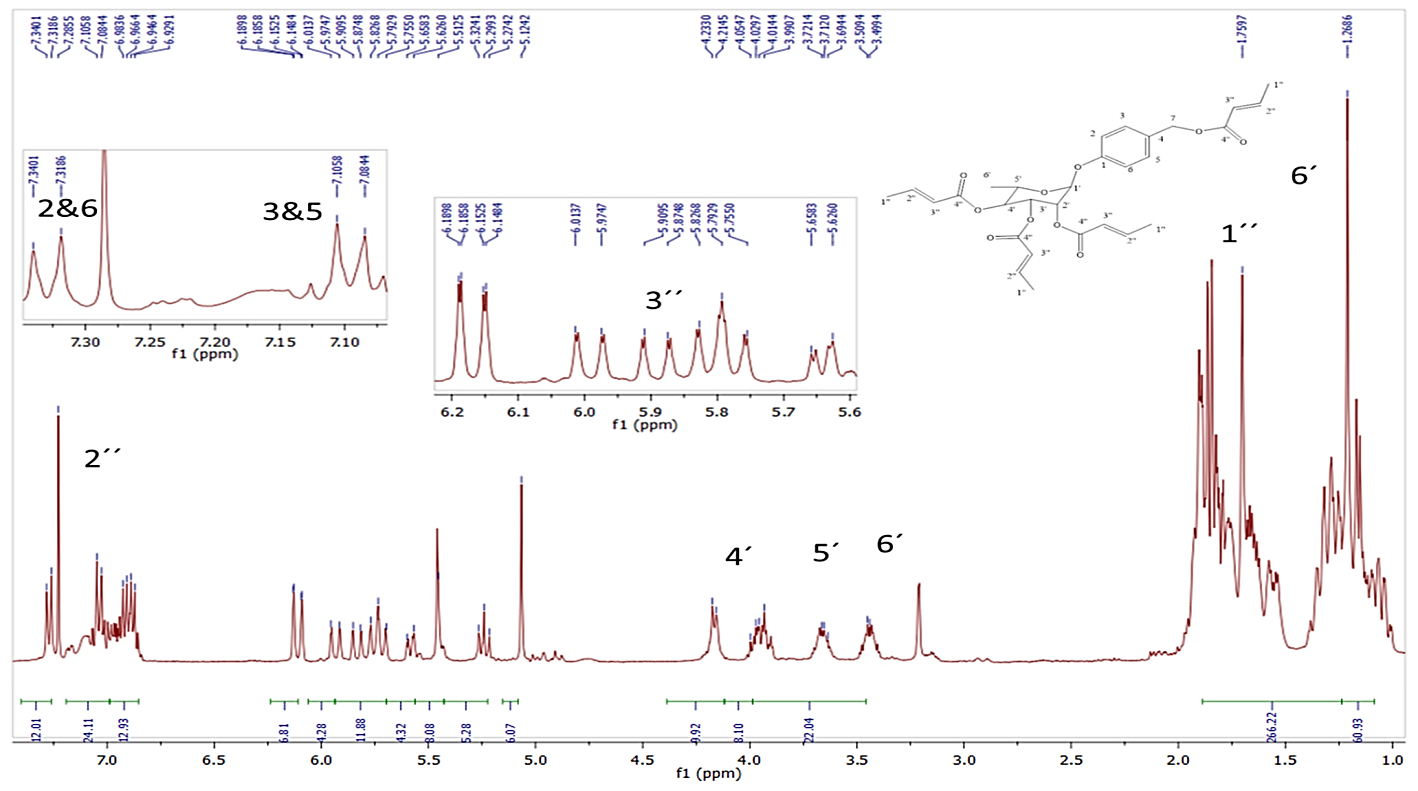


**Fig S18: ^1^H-NMR spectrum of compound S2_M2_**


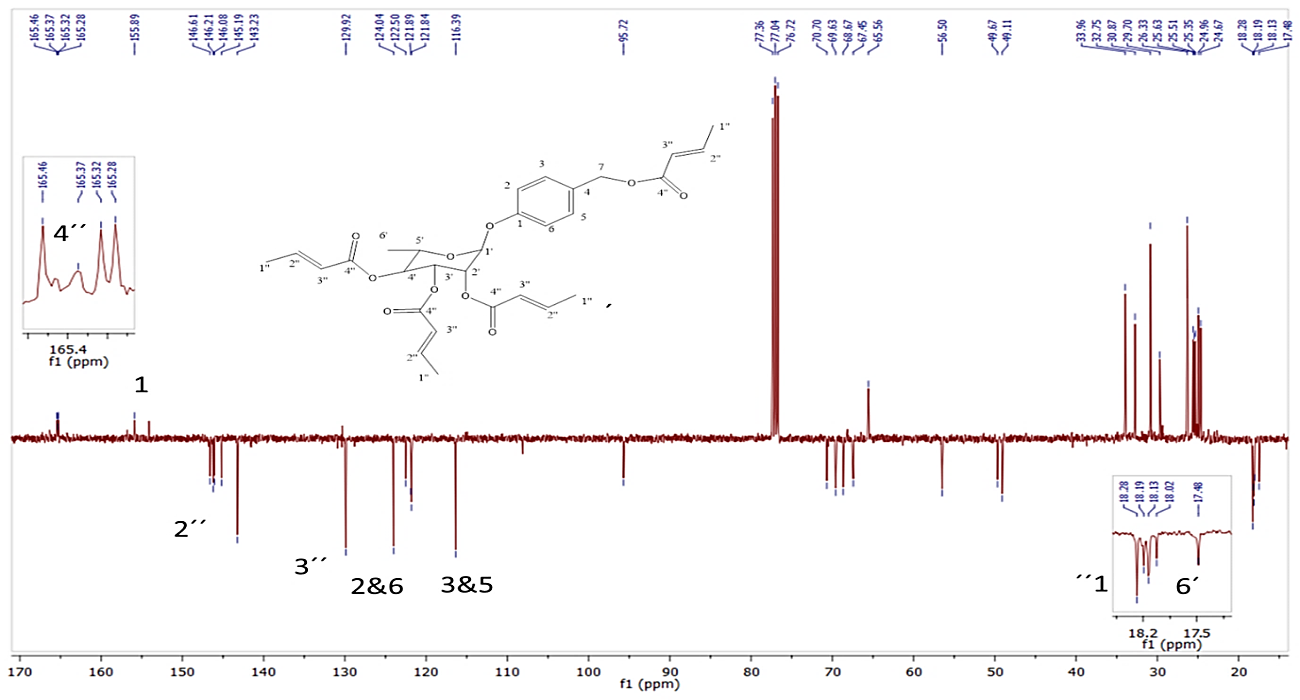


**Fig S19: APT-NMR of compound S_2M2_ spectrum**

**
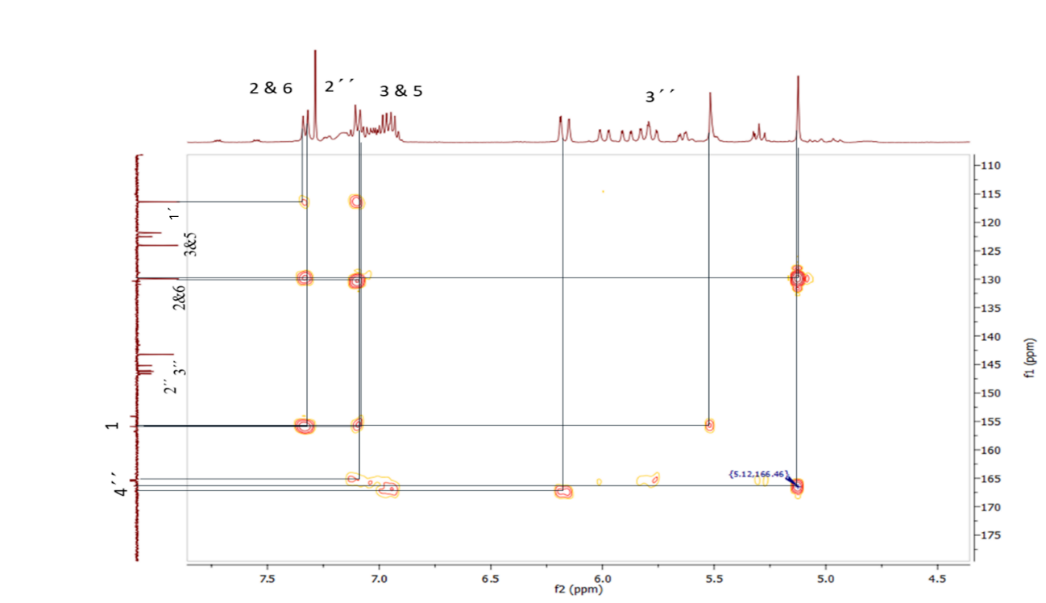
**

**Fig. S20: HMBC spectrum of compound S2_M2_**
